# Structural modelling of the DNAJB6 oligomeric chaperone shows a peptide-binding cleft lined with conserved S/T-residues at the dimer interface

**DOI:** 10.1038/s41598-018-23035-9

**Published:** 2018-03-26

**Authors:** Christopher A. G. Söderberg, Cecilia Månsson, Katja Bernfur, Gudrun Rutsdottir, Johan Härmark, Sreekanth Rajan, Salam Al-Karadaghi, Morten Rasmussen, Peter Höjrup, Hans Hebert, Cecilia Emanuelsson

**Affiliations:** 1 MAX IV Laboratory, Lund University, PO Box 118, SE-221 00 Lund, Sweden; 2 Department of Biochemistry and Structural Biology, Center for Molecular Protein Science, Lund University, PO Box 124, SE-221 00 Lund, Sweden; 3 School of Technology and Health, KTH Royal Institute of Technology and Department of Biosciences and Nutrition, Karolinska Institute, Stockholm, Sweden; 4 School of Biological Sciences, Nanyang Technological University, Singapore, 637551 Singapore; 5 Department of Biochemistry and Molecular Biology, University of Southern Denmark, Odense, Denmark

**Keywords:** Peptides, Chaperones, Molecular neuroscience, Molecular modelling, SAXS

## Abstract

The remarkably efficient suppression of amyloid fibril formation by the DNAJB6 chaperone is dependent on a set of conserved S/T-residues and an oligomeric structure, features unusual among DNAJ chaperones. We explored the structure of DNAJB6 using a combination of structural methods. Lysine-specific crosslinking mass spectrometry provided distance constraints to select a homology model of the DNAJB6 monomer, which was subsequently used in crosslink-assisted docking to generate a dimer model. A peptide-binding cleft lined with S/T-residues is formed at the monomer-monomer interface. Mixed isotope crosslinking showed that the oligomers are dynamic entities that exchange subunits. The purified protein is well folded, soluble and composed of oligomers with a varying number of subunits according to small-angle X-ray scattering (SAXS). Elongated particles (160 × 120 Å) were detected by electron microscopy and single particle reconstruction resulted in a density map of 20 Å resolution into which the DNAJB6 dimers fit. The structure of the oligomer and the S/T-rich region is of great importance for the understanding of the function of DNAJB6 and how it can bind aggregation-prone peptides and prevent amyloid diseases.

## Introduction

The human molecular chaperone DNAJB6 is a member of the highly diverse family of DNAJ chaperones^1,2^ and a remarkably efficient suppressor of amyloid fibril formation, with a potential for disease targeting. The DNAJ-proteins, first recognized as co-chaperones in *E. coli*^3^, form the largest and functionally most diverse chaperone family^4,5^. The N-terminal domain (NTD) is a conserved so called J domain, folded into four α-helices. This domain is the defining domain for this class of chaperones and through a conserved HPD-motif it is essential for the functional interaction with Hsp70^6^. There are 49 human DNAJ homologues divided into classes DNAJA, DNAJB and DNAJC, with large structural and functional diversities both within and between the classes. One of the most studied DNAJ-proteins is the heat-induced DNAJB1, the human homologue is known as Hdj1^7,8^ and the yeast homologue as Sis1^9^. DNAJB1 forms dimers through the peptide-binding C-terminal domain, which contains two β-barrel domains.

Human DNAJB6 was cloned in 1999, at that time named Mrj (mammalian relative to DNAJ), and found to be distributed in various tissues throughout the body^10^. It was also identified by gene trapping as essential for murine placental development^11^. Using yeast-two-hybrid screening, DNAJB6 was identified as a specific binder to keratin-18^12^. In addition, it was found to be brain-enriched^13^ and to inhibit polyQ aggregation, in *Drosophila*^14,15^ and in human cell lines^13,16^. The importance of DNAJB6 in placental development, and the failure in chorioallantoic attachment in knock-out mice, appear to relate to its role in keratin degradation^17^. Other roles assigned to DNAJB6 include inhibition of transcription factor activity through histone deacetylase recruitment in activated T-cells^18^, and a role in proteasomal turnover and autophagy^17,19^ and in cell cycle^20,21^, presumably through mediating keratin turnover and reorganization of cellular skeletal proteins during cell division. DNAJB6 is also highly expressed in myocytes, with mutations causing muscular dystrophy^22^ and amyloid formation, as shown in muscle biopsies^23^. There are several isoforms of DNAJB6, of which two are confirmed to be expressed, one nuclear (A) and the second cytosolic (B)^24^. The cytosolic isoform can also translocate into nucleus during heat shock^25^. The results reported here all relate to the cytosolic isoform of DNAJB6 (Uniprot #O75190-2).

According to recent results obtained by us and our collaborators^26–30^, DNAJB6 is a chaperone that may have a general role in preventing amyloid formation^31^, caused by peptides generated from full-length proteins during catabolic events. Indeed, expression network analyses show that DNAJB6 expression is strongly associated with catabolic processes (Fig. S7 in^30^). Aggregation of polyQ peptides is suppressed by DNAJB6, in human cell lines^27^ and in controlled assays with purified protein^26^. DNAJB6 can also suppress amyloid fibril formation by Aβ42, an aggregation-prone peptide associated with Alzheimer’s disease^28^, at remarkably low sub-stoichiometric levels, more efficiently than any other known chaperone. The molecular mechanism of the suppression of aggregation and amyloid fibril formation by the Aβ42 peptide is related to the inhibition of the primary nucleation step, as shown by kinetic analysis of the process^28,29^. Through site-specific mutations in the so-called S/T-rich region, a conserved and functionally important region rich in serine and threonine residues, we found that the potency of DNAJB6 to inhibit primary nucleation and suppress fibril formation by aggregation-prone polyQ peptides declined in proportion to the number of alanine substitutions^30^. The hydroxyl groups in the side chains may reduce the primary nucleation rate by competing with hydrogen bonding, which is necessary for formation of β-hairpins and amyloid fibrils^32^. The co-localization of many hydrogen-bonding groups in DNAJB6 will provide a synergistic or cooperative effect, which will gradually be lost with progressive S/T-to-A substitutions. We have also noted that although the ability of DNAJB6 to suppress amyloid fibril formation by polyQ peptides is mechanistically independent of the presence of Hsp70^26^, a dependency on Hsp70 can be detected under cellular conditions when DNAJB6 capacity is limited^30^. In cells, Hsp70 is presumably involved in the turnover and degradation of DNAJB6 and its bound peptides. Together, these findings imply that DNAJB6 can be used as a tool to facilitate the understanding of the aggregation process and its inhibition, as well as in the development of future treatment of amyloid diseases. Therefore, the mechanism of the highly efficient interactions of DNAJB6 with aggregation-prone peptides needs to be explored further.

The interactions of DNAJB6 with peptides depend on its structural organization, with unique features among DNAJ-proteins in terms of both oligomerization and the presence of the S/T-rich region. The size and polydisperse character of the DNAJB6 oligomers so far prevented structural studies by X-ray crystallography and NMR. In this work, we have combined homology modelling, chemical crosslinking mass spectrometry, small-angle X-ray scattering (SAXS) and negative stain electron microscopy (EM) to obtain a structural model of the monomer, dimer and higher order oligomeric forms of DNAJB6. The dimer model reveals a peptide-binding cleft lined with S/T-residues located at the interface between the two monomers in the DNAJB6 dimer. Detection of ^14^N-^15^N hybrid crosslinks shows that the DNAJB6 oligomers are dynamic entities that can exchange subunits and SAXS data indicate well folded DNAJB6 oligomers, forming a heterogeneous population with varying number of subunits, into which the structural model of the DNAJB6 dimers fit.

## Results

### Structural model of DNAJB6 monomer

**Figure 1:**
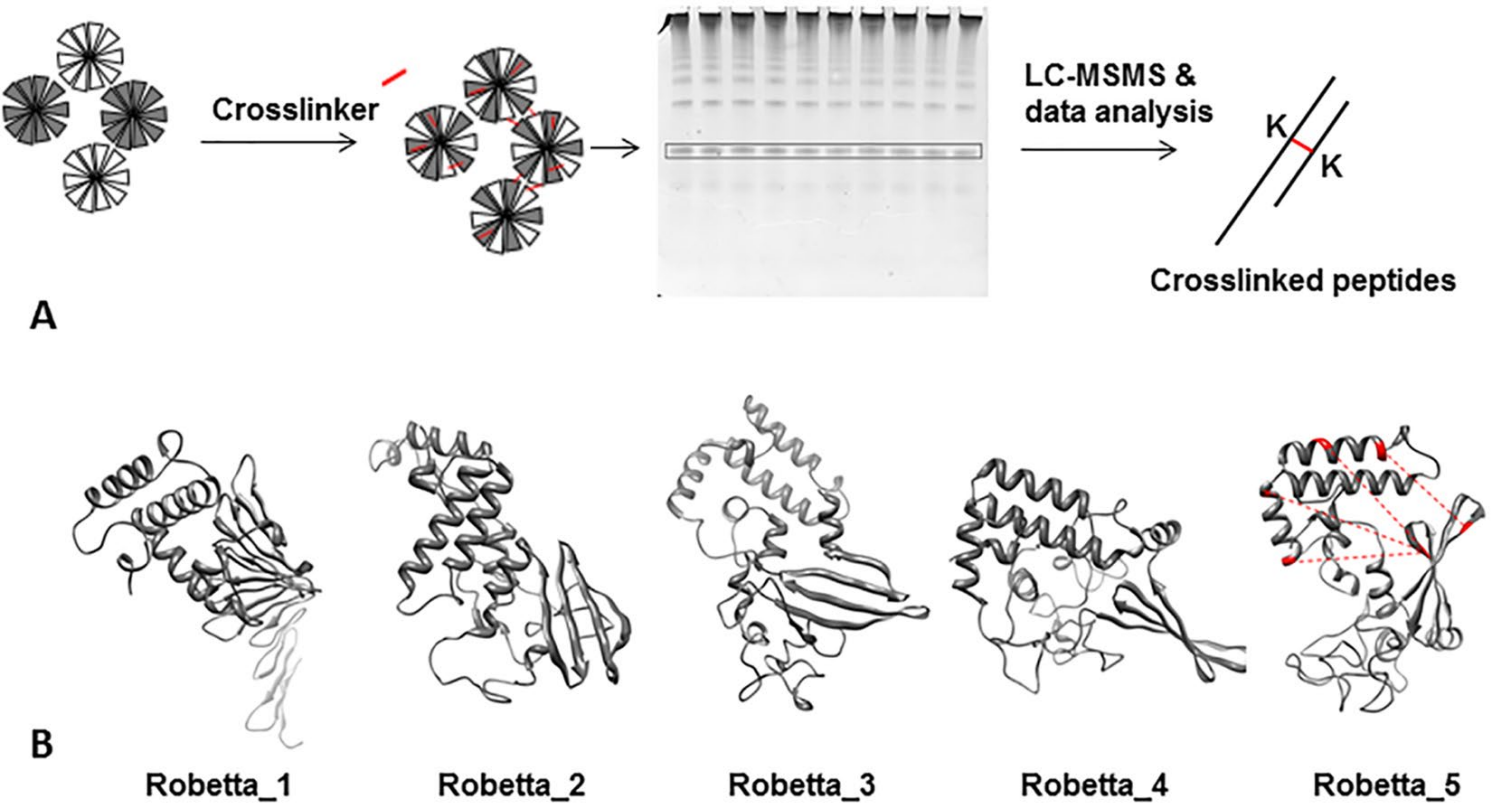
Chemical crosslinking mass spectrometry to select a best fit structural model of a DNAJB6 monomer. (**A**) Samples of DNAJB6 were crosslinked and bands corresponding to crosslinked monomer (enboxed) that contain only intra-subunit crosslinks were proteolytically digested, thereafter the peptide mixture was subjected to LC-MSMS and data analysis with the software MassAI to detect the crosslinked peptides. (**B**) The top five structural models from Rosetta modeling in cartoon presentation, and Robetta_5 was selected as best fit to the distance constraints (indicated as dashed lines in red) obtained from the crosslinking data in Table 1.

A structural model of the DNAJB6 monomer was selected after homology modelling based on intra-molecular distance constraints obtained from crosslinking mass spectrometry. To ensure that crosslinks used as distance constraints were intra-subunit, only crosslinks that were detected in the band corresponding to monomers (Fig. 1A) were used to assess the five models returned by the Robetta server (Fig. 1B). The choice of the output models, according to the original publication^33^ on comparative modelling with RosettaCM, is that the final models which may be generated from different seed alignment) are collected and the best 10% of the models by energy is identified, then clustered and the center of the largest cluster where each model is weighted such that low-energy models have highest weight is selected. Out of the five models in the Robetta server output, Model #5 was the only model which satisfied the experimental data (Table 1). The crosslinking data were compared to the Cα-Cα distances between each pair of lysine residues within the five structural models were calculated by the MassAI software after uploading each pdb-file of the models. The crosslinker can span distances up to approximately 30 Å^34^, due to crosslinker length, lysine side chain dynamics, backbone motions, movement of flexible loops and N- and C-terminal regions. The lysine residues that were detected in crosslinks were either in the N-terminal domain (NTD, residues 1–71) or in the C-terminal domain (CTD, residues 190–241). The intra-domain crosslinks were found to be within the allowed maximum of the Cα–Cα distance of 30 Å for all 5 homology models, not unexpectedly since each domain is <10 kDa, with a diameter of approximately 15 Å, which positions most amine groups within a distance that is reachable by crosslinking. The inter-domain crosslinks, on the other hand, made it possible to distinguish between the structural models. Model #5 satisfied two out of four interdomain crosslinks and showed no crosslinked distances exceeding 40 Å, so called violating crosslinks, in contrast to the other four models. Thus, model #5 was selected as best-fit and used for further analyses and for building a dimer model. The structural model of the DNAJB6 monomer was evaluated (Fig. S1), and found to have stereochemical quality comparable to that observed for high-resolution experimental structures. The file ‘DNAJB6 monomer model_Robetta_5.pdb’ is enclosed in Supplementary Information.

**Table 1:** Crosslinks detected within DNAJB6 monomers.

Precursor mass	Charge	Score	Crosslinked peptides^a^	X^b^	LysA	LysB	Distances (Å) in structural model^c^				
							#1	#2	#3	#4	#5
2291.355	4+	51.2	HASPEDIKKAYR + KLALK	(1)	K20	K25	8.7	8.7	8.7	8.7	8.7
1245.768	3+	29.4	KAYR + KLALK	(1)	K21	K25	6.1	6.0	6.1	6.1	6.1
1973.062	4+	40.8	WHPDKNPENK + KLALK	(1)	K34	K25	11.9	11.9	11.9	12.0	11.9
2688.340	3+	53.1	QVAEAYEVLSDAKK + DIYDKYGK	(1)	K60	K67	10.5	10.5	10.5	10.4	10.7
1934.032	3+	39.1	SISTSTKMVNGR + ALKW	(1)	K196	K29	66.6	34.4	37.3	44.5	22.4
1978.025	3+	14.6	KSLTINGK + KAYRK	(2)	K225	K21	77.9	28.1	45.1	44.6	32.8
2443.217	3+	63.6	VEVEEDGQLKSL + EVLSDAKK	(2)	K225	K60	58.2	46.9	55.3	50.5	36.2
1978.024	3+	24.9	KSLTINGK + DKYGKEGL	(2)	K225	K70	51.8	56.2	50.5	47.7	28.7

^a)^All detected crosslinks are intra-subunit as described in Fig. 1, approved MSMS-spectra used to validate the crosslinks supplied in Fig. S5.

^b)^Crosslinker X was either BS3 (1) or BS2-G (2) which are very similar.

^c)^Calculated Cα-Cα distances for crosslinked lysine residues in the pdb files of the DNAJB6 Robetta structural models #1–5 (Fig. 1). The DNAJB6 monomer is composed of the N-terminal domain (NTD, residues 1–71), the C-terminal domain (CTD, residues 190–241), and a disordered middle domain (residues 72–189) and detected crosslinks in table are intra-domain (upper part) or inter-domain (lower part). Model #5 is satisfying two out of four inter-domain crosslinks with no crosslinked distances exceeding 40 Å, in contrast to the other four models.

**Figure 2:**
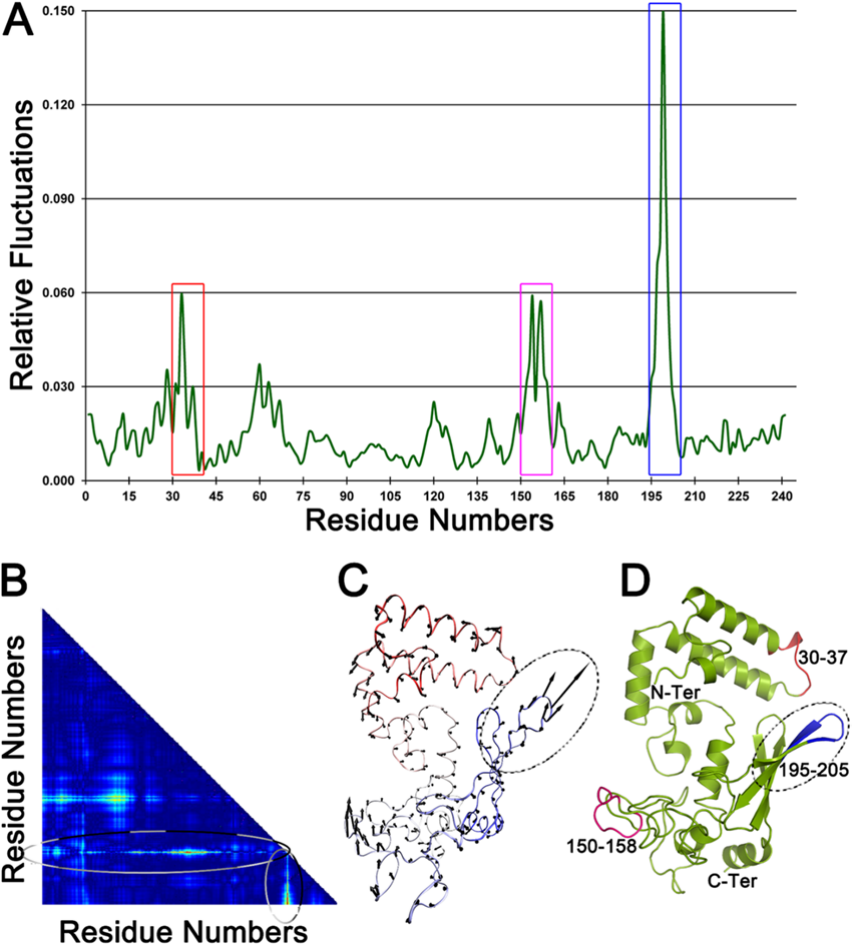
The conformational flexibility of DNAJB6 probed using normal mode analysis. The structural model of DNAJB6 monomer (selected as best fit, Fig. 1) was used for analysis. (**A**) The fluctuations of mode 9 reveal that the region 195–205 (within blue box) undergoes maximum conformational changes. (**B**) The regions with maximum (red) and minimum (blue) deviation represented using the contact map also reflect the previous observation. (**C**) The vector representation of the Cα displacements, with each vector represented as arrows along with their relative direction of motion and magnitude, also reveals the region with maximum displacement (within ellipse). The Cα trace is shown for reference colored from red (N-terminal) to blue (C-terminal). (**D**) A cartoon representation, indicating the loop region with the maximum fluctuation colored in blue, while the other two loops are colored red and pink.

The conformational flexibility of the DNAJB6 monomer was analyzed by normal mode analysis (NMA) simulations^35^. Comparison of predicted normal modes with transitions derived from multiple conformers of the same structures obtained by X-ray crystallography suggest that low-frequency normal modes are often functionally relevant^36^. Here, the low-frequency modes 7 to 11 from the structural model of the DNAJB6 monomer were analyzed to identify flexible regions, with all modes showing qualitatively similar results. As an example, mode 9 is shown in Fig. 2 and in the file ‘mode-9-movie.mov’ which is enclosed in Supplementary Information. A higher degree of flexibility in the loop regions is seen compared to the other regions of the protein. In particular, the loop between β-strands 1 and 2, with amino acid residues N199 and G200, possessed maximum conformational flexibility. This highly mobile loop may be involved in contacts between subunits within the DNAJB6 oligomer.

### Structural model of DNAJB6 dimer

The crosslinked samples were also analyzed without prior fractionation to restrict the analysis to intra-subunit crosslinks and, in order to also explore the interactions between DNAJB6 and Aβ42 that prevent its aggregation, crosslinking was performed also in the presence of Aβ42, and subsequently DNAJB6-DNAJB6 and DNAJB6-Aβ42 crosslinks were identified (Table 2). Concerning the DNAJB6-DNAJB6 crosslinks, although intra-subunit crosslinks are most frequently detected, most of the detected crosslinks are presumably both intra- and inter-subunit crosslinks. The obvious exception is the same-to-same-residue type of crosslinks, here K189-K189 and K232-K232, which can only be inter-subunit. These crosslinks are located in the CTD and therefore provide evidence for inter-subunit contacts in the CTD. Concerning the DNAJB6-Aβ42 crosslinks, four lysine residues in DNAJB6 (K189, K202, K225 and K232) were involved, suggesting that the CTD is involved not only in the inter-subunit contacts, but also in peptide binding.

**Table 2:** Crosslinks detected within DNAJB6 oligomers and between DNAJB6 and Aβ42.

**Precursor mass**	**Charge**	**Score**	**Crosslinked peptides DNAJB6-DNAJB6**^**a**^	**LysA**	**LysB**	**#1–2**^**b**^	**Distance**^**c**^**(Å)**
3101.577	3+	52.9	HASPEDIKKAYR + QVAEAYEVLSDAKK	K20	K60	1	16.6
1708.898	3+	69.4	HASPEDIKK + SDAKK	K20	K60	2	16.6
2012.068	3+	78.3	HASPEDIKK + KSISTSTK	K20	K189	1,2	36.0
1750.981	3+	85.4	HASPEDIKK + KITTK	K20	K202	1,2	29.6
2532.388	3+	56.6	HASPEDIKK + SLTINGKEQLL	K20	K232	2	47.2
2587.334	4+	46.3	WHPDKNPENKEEAER + KLALK	K34	K25	2	12.0
2899.498	4+	57.3	HPDKNPENKEEAER + KITTKR	K34	K202	1	16.5
2116.153	3+	54.6	KQVAEAY + TINGKEQLL	K47	K232	1	36.4
2154.108	3+	57.1	EVLSDAKKR + DIYDKY	K60	K67	1	11.0
2275.082	3+	63.7	GKEGLNGGGGGGSHF + KQVAEAY	K70	K47	1	21.6
2439.121	3+	52.6	GKEGLNGGGGGGSHF + DIYDKY	K70	K67	1	5.4
2812.348	3+	61.4	GKEGLNGGGGGGSHF + VEVEEDGQLKSL	K70	K225	1	28.3
1839.014	4+	72.3	KSISTSTK + KSISTSTK	K189	K189	2,*	—
2506.276	3+	54.6	VEVEEDGQLKSL + HASPEDIKK	K225	K20	1,2	33.1
3360.603	3+	65.2	VEVEEDGQLKSL + WHPDKNPENKEEAER	K225	K34	2	25.4
2371.237	3+	88.8	VEVEEDGQLKSL + EVLSDAKK	K225	K60	2	36.8
2483.232	3+	80.6	VEVEEDGQLKSL + DIYDGYGK	K225	K67	1,2	31.7
2492.265	3+	66.3	VEVEEDGQLKSL + SISTSTKM	K225	K196	1,2	14.5
2072.121	3+	79.5	VEVEEDGQLKSL + KITTK	K225	K202	2	12.3
2853.531	3+	77.8	VEVEEDGQLKSL + SLTINGKEQLL	K225	K232	1,2	16.1
2309.220	3+	67.4	TINGKEQLLR + DIYDKYGK	K232	K67	2	40.7
1198.116	3+	54.2	TINGKEQLL + KITTK	K232	K202	1	25.2
2679.516	3+	79.4	SLTINGKEQLL + TINGKEQLLR	K232	K232	1,2,*	—
**Precursor mass**	**Charge**	**Score**	**Crosslinked peptides**^**a**^**Aβ42-DNAJB6**	**LysA**	**LysB**		
2437.218	3+	48.8	HDSGYEVHHQKL + KSISTSTK	K16	K189	2	
2384.301	3+	53.8	EVHHQKL + VEVEEDGQLKSL	K16	K225	2	
2348.265	3+	81.4	FAEDVGSNKGAIIGLM + KITTK	K28	K202	2	
3129.674	3+	59.3	FAEDVGSNKGAIIGLM + SLTINGKEQLL	K28	K232	2	

^a^Crosslinks with BS3 within DNAJB6 can be either intra-subunit or inter-subunit, except the K189xK189 and K232xK232 crosslinks which must be inter-subunit.

^b^Datasets are: (#1) duplicate samples with crosslinked DNAJB6 oligomers, (#2) duplicate samples in which DNAJB6 oligomers and Aβ42. Approved MSMS-spectra used to validate the crosslinks are supplied in Fig. S6 (#1, 2). As indicated with asterisk (*), the inter-subunit crosslinks K189-K89 and K232-K232 are validated as inter-subunit also as ^14^N-^15^N hybrid crosslinks in a separate dataset with unlabeled (^14^N) and labeled (^15^N) DNAJB6, approved MSMS-spectra are supplied in Fig. S7.

^c^Calculated Cα-Cα distances for crosslinked lysine residues using the pdb file of the DNAJB6 Robetta structural model_5 described in Fig. 1.

**Figure 3:**
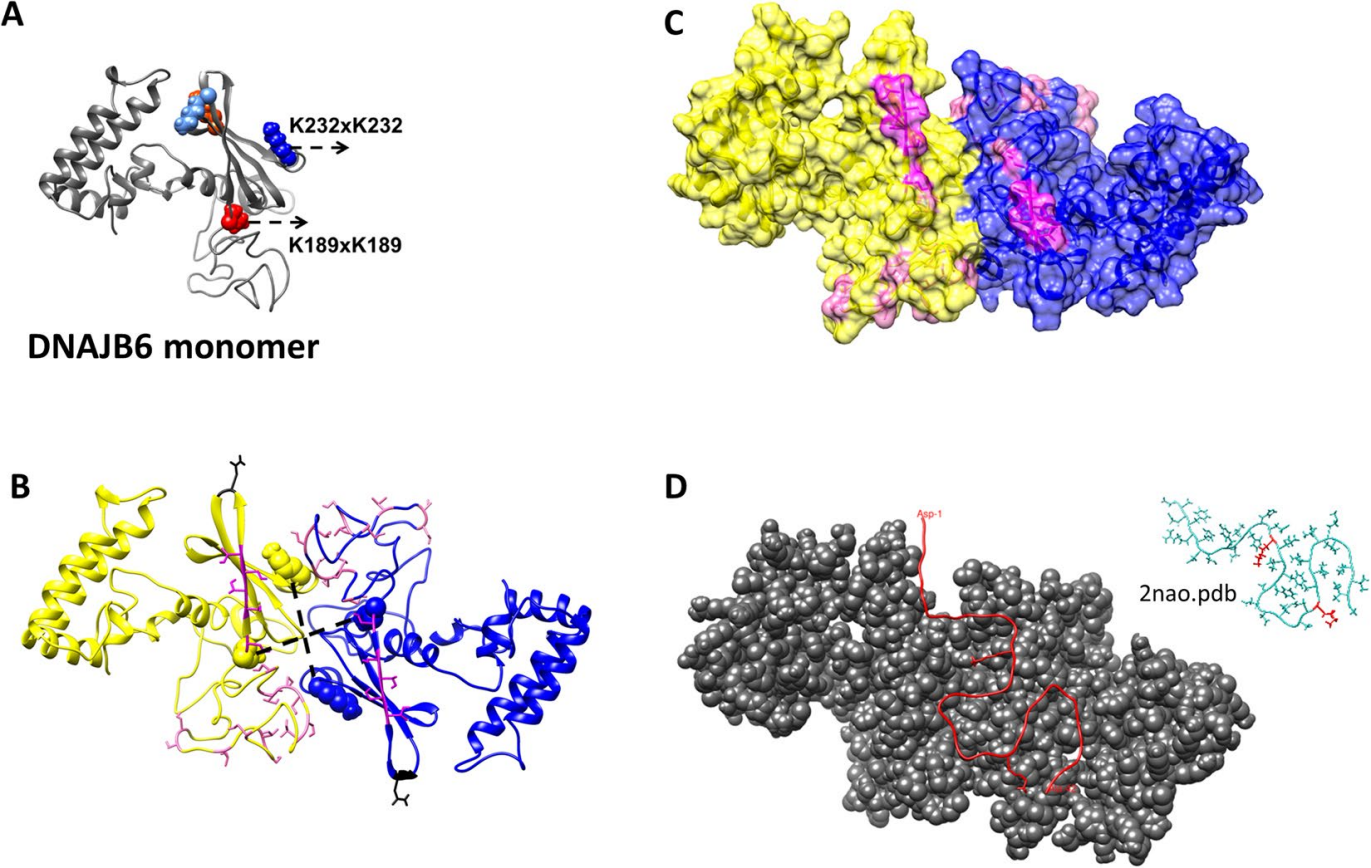
Structural model of DNAJB6 dimer. (**A**) The structural model of DNAJB6 monomer (selected as best fit, Fig. 1) in ribbon presentation (grey) with lysine residues as spheres: K189 (dark red), K232 (dark blue), K202 (light blue), K225 (light red). The inter-subunit crosslinks K189-K189 and K232-K232 (Table 2) were used as restraints in docking. (**B**) The DNAJB6 dimer model as cartoon, with the two subunits in yellow and blue, respectively. The lysine residues K189 and K232 (spheres, color coded according to each subunit) are shown with the inter-subunit crosslinks indicated with dashed lines, with distances K189-K189 = 23.5 Å and K232-K232 = 20.6 Å. The following residues are shown as sticks: N199, G200 (black); the S/T-residues S-STST aa 190, 192–195 (dark pink); other ST-residues in region aa 155–195 (light pink). The two symmetrically positioned β-hairpins (=β-strand 1 and 2 and the residues N199 and G200 in between) contain the functionally essential S/T residues. (**C**) The DNAJB6 dimer model in surface presentation color-coded as in (**B**), showing a central cleft between the domains which could serve as a peptide-binding cleft, surrounded by S/T-residues, S-STST aa 190, 192–195 (dark pink); other ST-residues in region aa 155–195 (light pink). (**D**) The DNAJB6 dimer model in spacefill presentation (grey) and one copy of Aβ42-peptide (red, with residues K16 and K28 as sticks). The image is obtained after HADDOCK docking of the Aβ42 (PDB ID 2NAO, shown as inset with the two K16 and K28 highlighted in red) to the DNAJB6 dimer model, using as restraints the crosslinked residues (K16 and K28 in Aβ42, K189, K232, K202 and K225 in one of the DNAJB6 dimer subunits).

The lysine residues in DNAJB6 involved in inter-subunit contacts and/or peptide binding are highlighted in the image of the DNAJB6 monomer in Fig. 3A. The crosslinked residues K189 and K232 were used as restraints in the docking of two DNAJB6 monomers to construct a DNAJB6 dimer. The docking program HADDOCK^37^ clustered 153 structures in 10 clusters, representing 76.5% of the water-refined models. The top cluster with a high HADDOCK score (−96.0 ± 4.8) shows a dimer with the CTD of each monomer contributing to the dimer interface (Fig. 3B), and with K189-K189 and K232-K232 at distances (dashed lines) 23.5 Å and 30.6 Å, respectively. Two symmetrically positioned β-hairpins, formed by the first two out of four β-stands in the CTD and the residues N199 and G200 in the loop between the strands, contain the conserved and functionally important S/T residues (high-lighted in pink). This clearly suggests that the S/T-residues are not buried but solvent-accessible and lining a central cleft formed at the interface between the two subunits, resulting in a possible site for peptide binding (Fig. 3C). The file ‘DNAJB6 dimer model_Haddock.pdb’ is enclosed in Supplementary Information.

That peptide binding indeed occurs in this peptide-binding cleft is supported by the detected DNAJB6-Aβ42 crosslinks (Table 2). There are three crosslinker-reactive primary amines in the Aβ42-peptide, one is the N-terminal amino group on the first residue and the other two are the primary amines in the side chains of the lysine residues K16 and K28 and only the latter two were crosslinked to DNAJB6. These crosslinks were used as restraints in another round of docking, using the above-mentioned pdb-file of the DNAJB6 dimer and, for Aβ42, the pdb-file 2NAO^38^. This resulted in a model showing a possible arrangement of how one copy of the Aβ42 peptide can be bound to the DNAJB6 dimer, as shown in Fig. 3D.

**Figure 4:**
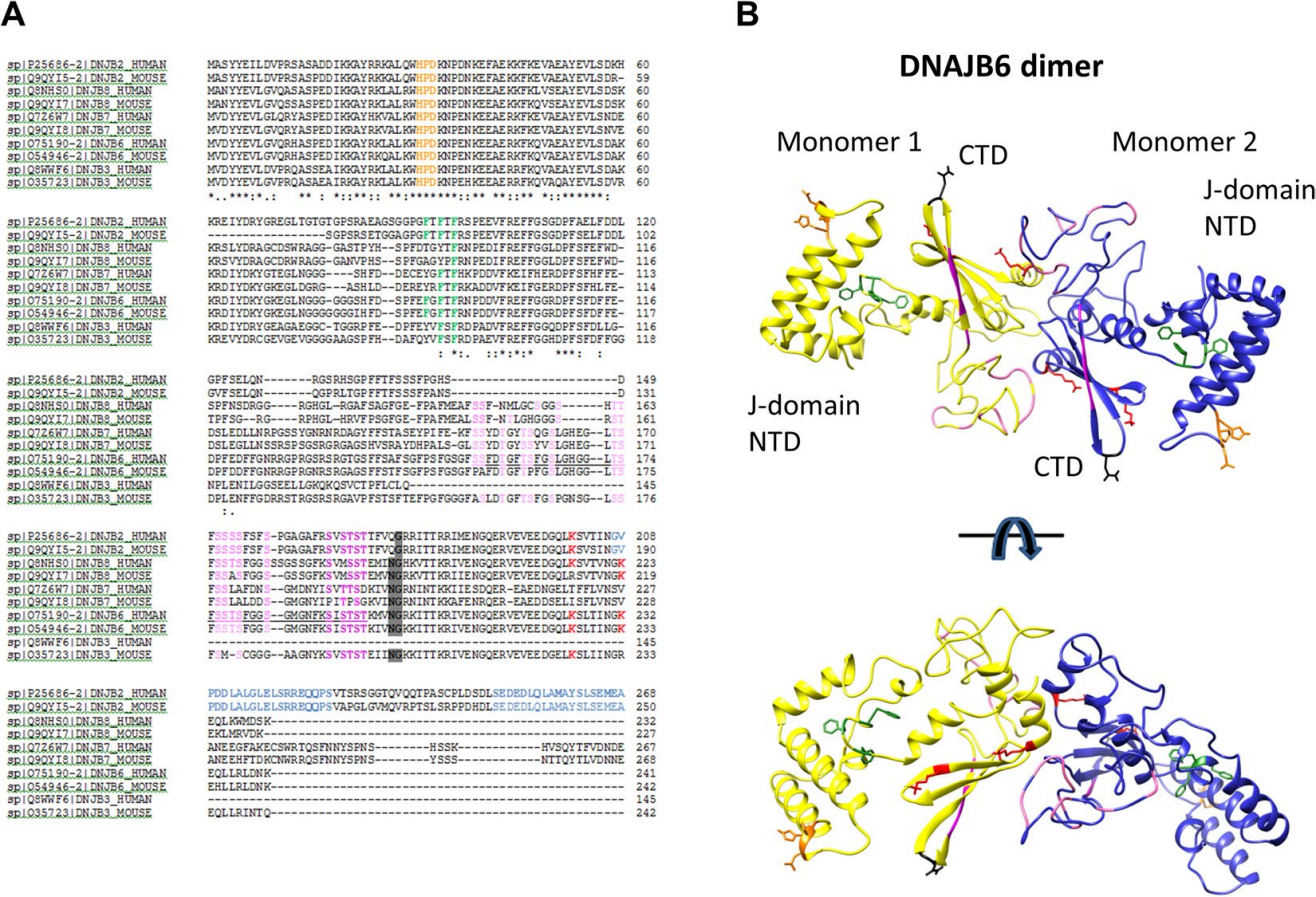
Sequence alignment of DNAJB6-homologues with features highlighted in the structural model of the DNAJB6 dimer. (**A**) Sequence alignment of human DNAJB2, 3, 6–8. The S/T-rich region in DNAJB6 (aa 155–195) is underlined with its 18 S/T-residues highlighted (pink), corresponding S/T-residues in other sequences highlighted similarly. Other highlights: the Hsp70-interaction HPD-motif (orange); the disease-causing F-residues (mutations F89I, F91I/L, F93I/L in muscular dystrophy); the mobile loop residues, identified in Fig. 2, N199 and G200 (black-shaded); the K225/K232-residues (red) suggested to regulate activity through deacetylation; the two UIM (ubiquitin interaction motifs) unique to DNAJB2 (grey-shaded). Alignments were performed at http://www.ebi.ac.uk/Tools/msa/clustalo/. The last residues in CTR of DNAJB2 and DNAJB7, longer than the other ones, are not shown. (**B**) The structural model of the DNAJB6 dimer in Fig. 3 is here shown from two angles, with S/T-residues as cartoon, and other residues as sticks highlighted with same color-code as in the alignment.

This peptide-binding cleft lined with the S/T-residues, which here presented data are suggesting for DNAJB6, represents a peptide-binding site in the CTD that is distinctly different compared to that in the crystal structures of other DNAJ-proteins, DNAJA1 and DNAJB1^8,39^, recently suggested to be referred to as ‘double β-barrel J-proteins’^5^. Whereas DNAJB6 only has one they are composed of two β-barrel domains in the CTD. They also have no S/T-rich region, whereas this region is fully or partially conserved within a sub-group of DNAJ-proteins that we refer to as DNAJB6-like (DNAJB2, 3, 6–8), which cluster together in a phylogenetic tree (Fig. S2). The conserved S/T-rich region in the CTD, and other known features in DNAJ-proteins, such as the conserved Hsp70-binding HPD motif in the NTD and the muscular dystrophy-related phenylalanine residues in the GF-rich middle domain, are summarized in Fig. 4, highlighted and similarly color-coded in the sequence alignment and the DNAJB6 dimer model.

The crosslinks K189-K189 and K232-K232 that were used as restraints in docking the monomers to dimer were readily identified as inter-subunit crosslinks since they are same-to-same-residue type of crosslinks. Since these two crosslinks are very important in constructing the DNAJB6 dimer model, their identity as inter-subunit crosslinks were also verified by mixed isotope crosslinking to rule out the possibility that they could have been incorrectly assigned. We have previously showed that this approach is useful for proof of inter-subunit crosslinks in homo-oligomeric proteins^40^. Hybrid crosslinks, formed when a labeled (^15^N) peptide is crosslinked to an unlabeled (^14^N) peptide, are detected by our software MassAI (MS/MS-spectra supplied in Fig. S7. The K189-K189 and K232-K232 crosslinks were detected among other hybrid crosslinks in a sample of DNAJB6 oligomers (^14^N-^15^N, mixed 1:1) incubated 1 h before adding the crosslinker. However they were not detected in a control sample where unlabeled and labeled oligomers were crosslinked separately and mixed 1:1 before LC-MSMS step. The detection of ^14^N-^15^N hybrid crosslinks shows that the DNAJB6 oligomers are dynamic entities that exchange subunits on a time-scale of less than an hour.

### Structural model of DNAJB6 oligomers

**Figure 5:**
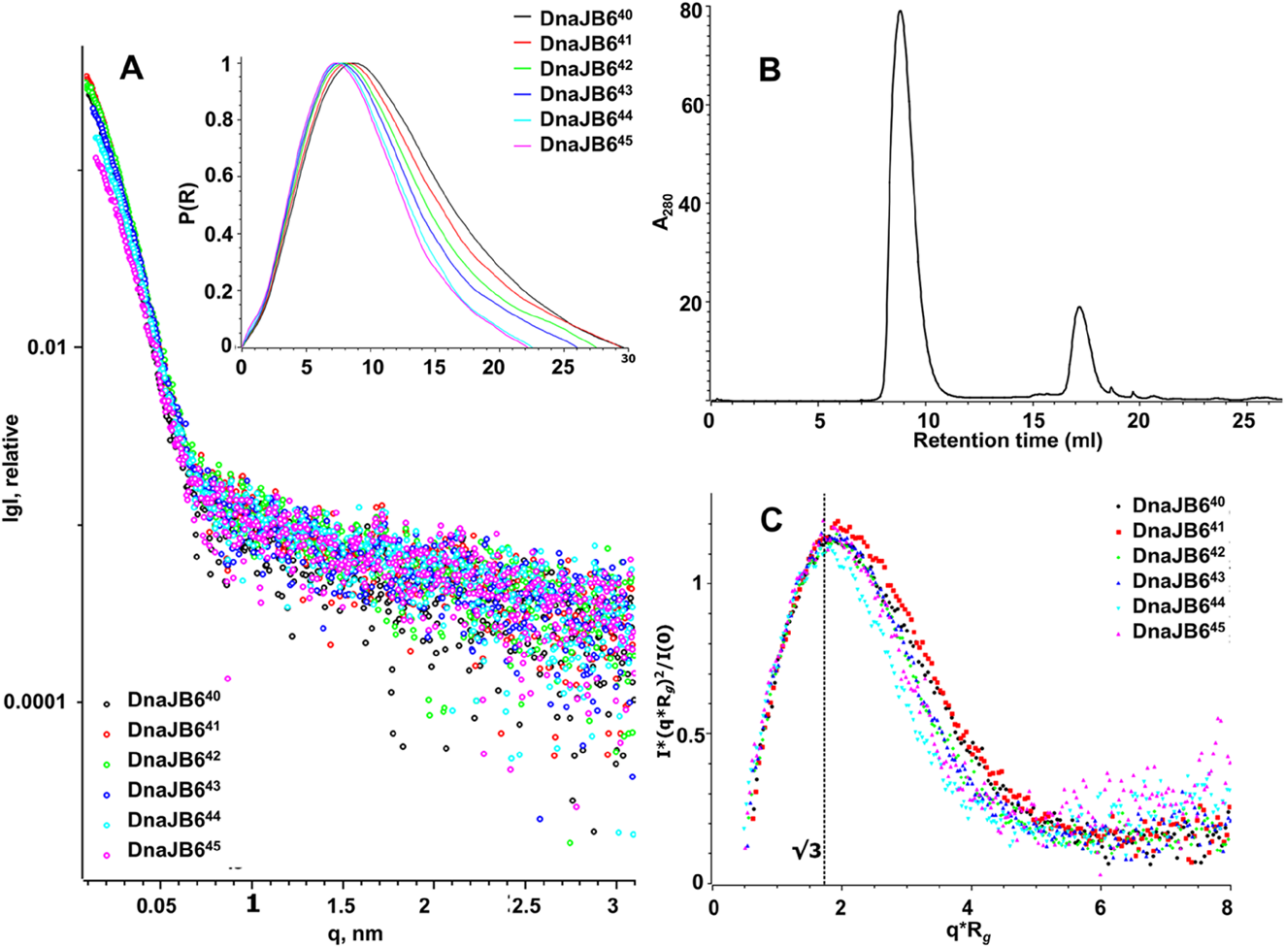
SAXS-data of DNAJB6. (**A**) Experimental online SEC-SAXS data with sample name numbers relating to retention times; the inset shows the P(R) distance distribution function of DNAJB6 calculated from the experimental SAXS data for estimation of D_max_ (Table 3). (**B**) The size-exclusion chromatography profile for DNAJB6, SAXS data shown are for the fractions from collected the main peak (8–11 ml) in the chromatogram, the peaks at >15 ml had too low scattering intensity. (**C**) The corresponding dimensionless Kratky plot of the SAXS data in (A).

That the purified DNAJB6 protein is composed of oligomers with a varying number of subunits, is well folded and is soluble up to 10 mg/ml is concluded from data obtained when the DNAJB6 oligomers were fractionated into six sub-populations by online size-exclusion chromatography combined with small-angle X-ray scattering (Fig. 5A). None of the scattering profiles, all derived from the main peak in the chromatogram (Fig. 5B), show any sign of aggregation, otherwise detected as a steep rise in a non-linear Guinier region of the scattering curve. The parabolic appearance of the Kratky plot (Fig. 5C), which can be used to assess the degree of fold of the scattering particles^41^, confirms our earlier data from circular dichroism spectroscopy that DNAJB6 is well folded. Flexibility or a deviation from a typical globular shape is observed for the DNAJB6 oligomers, since there is a peak maximum at a slightly higher value than 1.1 at qRg = √3. The radius of gyration (R_*g*_) and maximum dimension (D_*max*_), calculated from the P(r)-distribution curves (Fig. 5A, inset), ranged from 6.9 nm to 8.7 nm, and from 22.2 nm to 29.6 nm, respectively (Table 3). The estimated mass of the DNAJB6 oligomers calculated from SAXS data ranged from 0.8 MDa to 1.5 MDa, in line with our earlier data from dynamic light scattering^26^ and, since the monomeric mass of DNAJB6 is 26.9 kDa, corresponds to DNAJB6 oligomers with a varying number of subunits, ranging from 34 to 55.

**Table 3:** SEC-SAXS data.

**Sample**	^**a**^**R**_***g***_**, nm**	^**a**^**D**_***max***_**, nm**	^**a**^**V**_***p***_**, nm**^**3**^	^**b**^**MW**_***p***_**, kDa**	^**b**^**MW**_***a***_**, kDa**	^**c**^**Subunits**
DNAJB6^40^	8.73 ± 0.16	29.6	2368	1480	1450	55
DNAJB6^41^	8.50 ± 0.35	29.4	2114	1321	1240	49
DNAJB6^42^	7.70 ± 0.2	27.5	1849	1156	1090	43
DNAJB6^43^	7.43 ± 0.11	26.6	1669	1043	979	39
DNAJB6^44^	6.72 ± 0.25	22.5	1523	952	856	35
DNAJB6^45^	6.88 ± 0.19	22.2	1470	919	838	34

^a^R_g_, Radius of gyration, D_max_, maximum size, V_p_, Porod volume, calculated as described in Methods.

^b^MW_p_, MW_a_, mass estimates calculated by dividing V_p_ by 1.6 and by the DAMMIF model excluded volume by 2, respectively.

^c^Subunits, number of subunits fitting the average MW_*p*_ calculated with mass of the DNAJB6 26.9 kDa.

**Figure 6:**
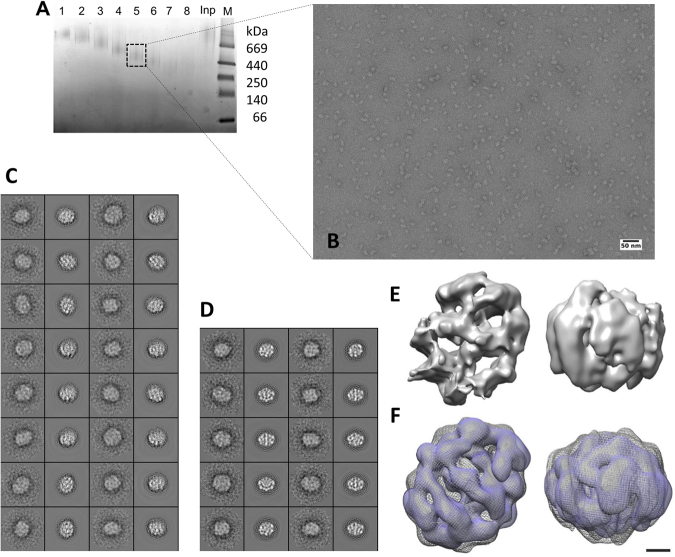
DNAJB6 oligomers analyzed by negative stain electron microscopy. (**A**) Insert shows native PAGE analysis of DNAJB6 fractions (1–8) collected after GraFix separation,’Inp’ DNAJB6 before GraFix. **(B)** EM image of DNAJB6 oligomers collected from fraction 5 in (**A**). (**C**,**D**) 2D class averages and corresponding projections of 3D maps obtained using C1 (**C**) and C2 (**D**) symmetry. (**E**) Asymmetric density map of DNAJB6 from two mutually perpendicular directions. (**F**) A comparison of a density map where 2-fold symmetry was imposed on the unsymmetrized C1 map (in mesh) and one density map where processing was done using C2 symmetry (in surface rendered blue). The maps are depicted at 5σ threshold. Scale bar represents 20 Å. See also Fourier shell correlation in Fig. S3.

The DNAJB6 oligomers with varying number of subunits were also detected by negative stain EM, and one sub-population of DNAJB6 oligomers was selected for closer analysis (Fig. 6). Reference-free classification of boxed out particles showed classes of slightly elongated particles with major and minor axes lengths of approximately 160 Å and 120 Å, respectively. The asymmetric 3D map showed an oligomeric complex, which can be fit into a box of 332 × 332 × 332 Å^3^. A two-fold symmetry was imposed to generate a map that was compared to the symmetry independent reconstruction. A high degree of similarity between these maps suggested that the analyzed fraction of DNAJB6 is symmetrical, at least at this low resolution level. The reconstructed map with C2-symmetry was obtained at a resolution of ~20 Å measured at 0.143 Fourier shell correlation (Fig. S3) and the file ‘dnajb6_negstain_c2.mrc’ is enclosed in Supplementary Information. Considering a particle mass of 540 kDa, very approximately estimated from non-denaturing PAGE and that one DNAJB6 subunit has the mass 26.9 kDa, this sub-population of DNAJB6 oligomers selected for single particle analysis corresponds approximately to 20 subunits.

**Figure 7:**
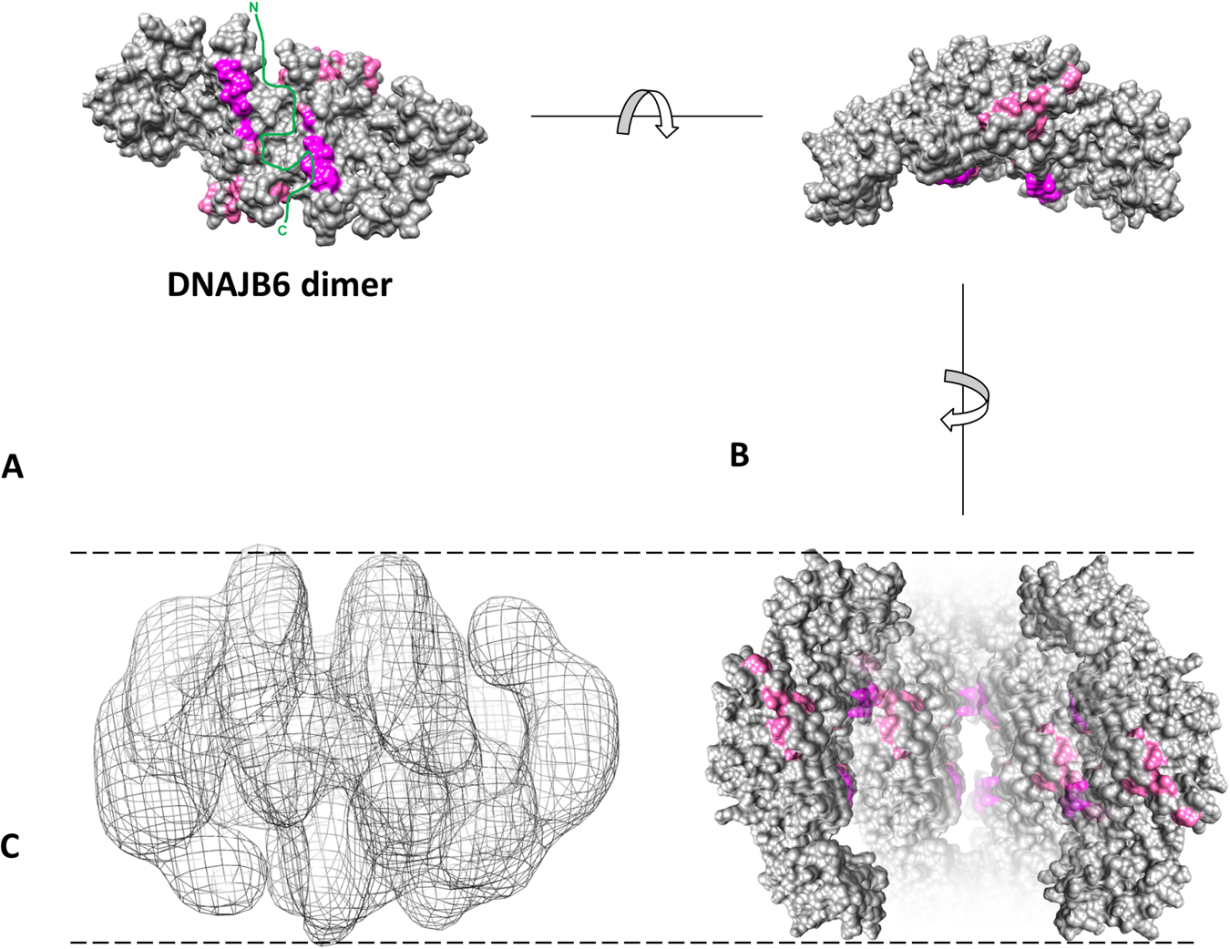
Structural model of DNAJB6 dimer model in relation to DNAJB6 oligomer. The DNAJB6 dimer model generated as described in Fig. 3 here presented in space-fill (grey), with a tentatively outlined Aβ42 peptide (green) at the dimer interface in a peptide-binding cleft, which may interact with aggregation-prone peptides in various conformations (e.g. one peptide, a pair of peptides or one peptide in hairpin conformation), and which is surrounded by the S/T-residues 190 and 192–195 close to the cleft (dark pink) and the other S/T-residues further away from the cleft (light pink). The conserved S/T-residues are required to suppress fibril formation, presumably due to hydrogen-bonding between the hydroxyl groups in the side chains of the S/T-residues and the aggregation-prone peptides^30^. In the lower part of the figure it is outlined how the DNAJB6 dimer can be turned to appear in a bent shape as a crescent with the S/T-residues 190 and 192–195 on the concave side and that such DNAJB6 dimers can be fitted to the DNAJB6 electron density map, generated as described in Fig. 6. In Fig. S4 is further described how the DNAJB6 dimers fit into the DNAJB6 oligomers.

The DNAJB6 dimers fit into the electron density map of the DNAJB6 oligomer. A structural model of the DNAJB6 dimer is presented in Fig. 7, with a peptide-binding cleft lined with S/T-residues at the dimer interface and a tentatively outlined Aβ42 peptide, similar to the image presented in Fig. 3D after crosslink-assisted docking. The S/T-residues in closest proximity to the peptide (the S/T-residues 190 and 192–195) are highlighted in dark pink and the other S/T-residues in light pink. The DNAJB6 dimer can be turned to appear in a bent shape as a crescent with the S/T-residues 190 and 192–195 on the concave side, and fitted to the electron density map of the DNAJB6 oligomer. The length of the dimer extends from one end of the oligomer to the other, with the N-terminal domains exposed towards the poles and the C-terminal domains located in the equatorial plane of the oligomer, as outlined in Fig. S4. An approximate volume calculation in Chimera at a threshold which is close to the noise-level yields a map volume of approximately 700 000 Å^3^, in which there would be space for approximately 10 dimers in total, i.e. 20 subunits. It should be noted that this is just one possible oligomer model, based on only one subfraction of DNAJB6 oligomers.

## Discussion

We here present a structural model of oligomeric DNAJB6. It is based on modelling to obtain first a monomer and then a dimer based on restraints from crosslinking, on SAXS-data showing that the preparation is well folded and on EM-data showing an oligomer at low resolution into which the dimers can be fitted. Only crosslinks that are validated by manual inspection of the MSMS-spectra were used as restraints in modelling. Crosslinked samples with only one or two proteins are not that complex and there is a redundancy of observations of each crosslinks. The ones that are presented in Tables 1 and 2 have been observed many times, with strict selection criteria imposed to avoid any false positive crosslinks.

### DNAJB6 dimer with a peptide binding site lined with S/T-residues

There are several reasons to expect that the DNAJB6 is composed of dimeric building blocks. Our recombinantly expressed and purified DNAJB6 protein shows not only the band corresponding to the mass of the 26.9 kDa monomer in SDS-PAGE, but also a band corresponding to the mass of a dimer at 60 kDa which is confirmed to contain DNAJB6 by mass spectrometry^28^. Furthermore, homologues for which the structure has been determined are dimeric, for example human DNAJB1 (PDB # 2QLD/3AGX)^7,8^, yeast DNAJB1 (1C3G/2B26)^9,42^, and yeast DNAJA1 (PDB # 1NLT/1XAO)^39,43^. The complete structure recently determined for a more distantly related homologue from *T. thermophilus* (PDB #4J80)^44^, also is composed of dimeric building blocks.

The structural model of the DNAJB6 monomer (Fig. 1) was obtained by modelling, and selection of best-fit based on crosslink constraints (Table 1) within the monomer. Since there is no suitable template for direct comparative modeling of DNAJB6, the Rosetta modelling was based on a protocol that determines the regions of a protein chain that align to different templates and find regions that may fold into globular units, which does not require a full template with high overall sequence identity. A structural model of the DNAJB6 dimer (Fig. 3) was then obtained by docking of two monomers with a software, in which additional information on the proximity of the protein interfaces is used to drive the docking process. As protein interface information, we used the lysine residues 189 and 232 in neighboring monomers (Table 2) as restraints. There is an ensemble of solutions as output from the HADDOCK server. Among the clusters with best energy the second best cluster was considerably less good (score −47.9 +/− 6.2) than the best (score −96.0 +/− 4.8). The lowest score is the most likely solution, and also low standard deviation is a positive factor. The score is based on an average of the best 4 models of each cluster together with the associated standard deviation. The distance constraints imposed by the crosslinking were largely met in the resulting dimer model, with K189-K189 at 23.5 Å distance and K232-K232 at 31.3 Å distance. Estimation of the buried surface area (2258.5 Å^2^) provides a reasonable value expected for a protein dimer interface^45^. An evaluation of the interface with the PISA server^46^ showed 65 interfacial residues, with 13 hydrogen bonds mainly between residues in the regions 175–178 and 232–234. Some may be hot spot residues critical in their contribution to the binding free energy^47^ and can be targeted for site-specific mutagenesis to evaluate the model.

An essential implication of the DNAJB6 dimer model is that the functionally important S/T-residues are not buried in the monomer-monomer interface (Fig. 3B,C). Instead, they surround a central cleft formed between the two CTD domains, one from each monomer. We suggest that this region can serve as a possible peptide-binding cleft with two symmetrically positioned sites, each having on one side five S/T-residues S-STST (aa 190, 192–195, color-coded dark pink) and on the other side the other ST-residues (in region aa 155–195, color-coded light pink). The S/T-rich region (residues 155–195) has in total 18S/T-residues that are to a varying extent conserved among DNAJB6 homologues (Figs 4 and S2) and the suppression of fibril formation and the inhibition of the primary nucleation is specifically dependent on the S/T-rich region^30^.

We have earlier proposed that S/T-residues provide a multivalent hydrogen-bonding capacity, whereby hydroxyl groups can reduce the primary nucleation rate by competing with the hydrogen bonding required for formation of β-hairpins and amyloid fibrils^32^. The detected crosslinks suggest that the possible peptide-binding cleft lined with the conserved S/T-residues is where DNAJB6 interacts with Aβ42, as shown in the image obtained after docking of the peptide (Fig. 3D) and as outlined in Fig. 7. Even if the detected crosslinks are correctly assigned it is impossible to tell if the detected crosslinks originate from one or several Aβ42-peptides, and if Aβ42 is disordered, is a β-hairpin or in some other form. Still, with the simple assumption of one peptide monomer we performed docking and, although one must be aware of and not overinterpret the results based on this assumption, it is possible to conclude (i) how all the detected crosslinks in DNAJB6 are localized in one interaction area, the presumed peptide binding cleft with the S/T-residues, (ii) how the N-terminal end of the Aβ42 peptide, with its primary amine not detected in crosslinks with DNAJB6, appears to point away from the interaction area, and (iii) how K16 and K28 in Aβ42, if they were in one and the same peptide, could be localized in close proximity to the S/T-residues. In the model presented in Fig. 3D, the sequence STST (192–195) in DNAJB6 was close enough to the Aβ42 peptide so that S194 and T195 could form hydrogen bonds to side-chains of SNK (26–28) in the peptide whereas S192 and T193 were closer to peptide residues with less polar side-chains. Although this is of some interest to note, the assumption of one peptide monomer is presumably not fully correct since DNAJB6 presumably does not bind Aβ42 monomers, according to our previous kinetic calculations^28,29^. Instead DNAJB6 binds aggregated forms of Aβ42, possibly already stacked as initiating amyloids with hydrogen bonding. There may still be unsatisfied hydrogens bonds in oligomeric assemblies and edge strands in such aggregated forms of Aβ42 that could bind to the S/T-rich region in DNAJB6.

Peptide binding is indeed expected to typically occur in clefts or surface pockets of proteins^48^. In DNAJB1 two peptide binding sites were found in CTD domain I, one of which was involved in binding an octapeptide to the C-terminal region of Hsp70 and the second contributed to recognizing ‘non-native polypeptide substrates’, with both binding sites being very hydrophobic^8^. The structure of the CTD in DNAJB1, and the mode of peptide binding is presumably distinctly different from that in DNAJB6.

The sofar known target peptides of DNAJB6, polyQ and Aβ42^26–30^, share the common property of being very aggregation-prone, with a high propensity to form amyloid fibrils. A dimer structure of Aβ42 has been observed in Aβ42 fibrils by cryo-EM^49^ and a hairpin conformation, assumed to occur in the pathway of fibril formation^50^, has been structurally characterized after capture of Aβ42 in complex with an anti-aggregation antibody^51^ or with an engineered lipocalin^52^. The crosslinks between Aβ42 and DNAJB6 that we here detected (Table 2) showed that crosslinking had occurred to both primary amines in the side chains of the two lysine residues, K16 and K28. However, no crosslink was detected that involved the third primary amine of the Aβ42-peptide, which is the N-terminal amine of the first amino acid, which is usually crosslinked in most proteins and previously detected in crosslinks between Aβ42 peptides^53^. This strongly indicates that it is the C-terminal rather than the N-terminal part of the Aβ42-peptide that interacts with DNAJB6. The Aβ peptide is comprised of a charged N-terminal and a hydrophobic C-terminal region, with a highly hydrophobic central region, in which the residues 16–21 (KLVFFA) constitute the most aggregation-prone segment and are alone sufficient to cause formation of insoluble fibrils^54^. A time-scale crosslinking study could possibly catch conformational changes in Aβ42 and monitor the DNAJB6 interference with the Aβ42 fibril formation, and explore the interactions between DNAJB6 and peptides.

### DNAJB6 oligomers with a varying number of subunits in dynamic subunit exchange

The dynamic character of DNAJB6 oligomers and the exchange of subunits is evident from the detection of inter-subunit ^14^N-^15^N hybrid crosslinks (Table 2, Fig. S7). The SAXS data suggest that the DNAJB6 oligomers are well folded and soluble (Fig. 5), with estimated subunit content in the range between 34 to 55 subunits, based on Porod volume calculations and the DAMMIF software (Table 3). Calculation of mass based on I(0), the sequence and the protein concentration^55^, requires a known protein concentration and was therefore not applicable after the online SEC-SAXS fractionation. The subpopulation of DNAJB6 oligomers analyzed by EM (Fig. 6) corresponds to 20 subunits, however the density map is obtained from just one of the smaller subpopulations of DNAJB6 oligomers, and it should be mentioned that negative stain EM often underestimates the volume. It can be concluded from both SAXS- and EM-data that DNAJB6 oligomers have a slightly elongated shape and a varying number of subunits, at least 20 and up to 55.

The oligomeric conformation of DNAJB6 may increase the local concentration of the peptide-binding interaction surfaces, may control peptide binding and release and may maintain continuous weak and transient interactions at sub-stoichiometric ratios. The oligomeric character of DNAJB6 resembles that of the small heat shock protein (sHsp) chaperones^16^. In spite of the importance of sHsps in cells, there are only a few high-resolution structures available, probably due to difficulties related to the dynamic character of the often polydisperese oligomers^56^, an inherent property that appears to be required for their ability to bind and sequester target proteins. There appears to be distinct structural and functional differences between on one hand the DNAJB1-like chaperones, recently suggested to have a dis-aggregase activity in complex with Hsp70^57^, and on the other hand DNAJB6-like chaperones. Within the sub-group of DNAJB6-like chaperones (Fig. S2), the S/T-rich region is present in DNAJB8 which is oligomeric, as DNAJB6, but expressed only in testes^16^. The S/T-rich region is found also in DNAJB2, but it is not known whether DNAJB2 is oligomeric or not. DNAJB2 is expressed in neuronal cells with ubiquitin interaction motifs that bring clients to the proteasome for degradation^58^. Some S/T-residues are conserved also in DNAJB3 and DNAJB7, which are less well known. Structural models will benefit and progress research on DNAJB6-like chaperones.

### DNAJB6 dimers fitted into DNAJB6 oligomers

One of the questions of particular interest to address concerning the DNAJB6 oligomers is where the functionally S/T-rich region, required to suppress fibril formation^30^, is situated. Is it exposed on the outer surface of the DNAJB6 oligomers, or is it hidden inside? In our DNAJB6 oligomer model with dimers fitted into the density map (Figs 7 and S4), the CTDs forming the dimer interface are located in the equatorial plane. The residues N199 and G200, in the mobile loop that was identified in the NMA simulations to be highly flexible (Fig. 4), are located in a position where they could form contacts between the dimers. The NTDs with the conserved Hsp70-interaction HPD-motif (residues 30–32) are accessible from the oligomer surface, whereas the S/T-rich region appears to face the interior of the oligomers. It remains to be investigated how peptides can get access to the S/T-rich region and the presumed peptide-binding cleft, if it is the oligomeric form of DNAJB6 that interacts with the aggregation-prone peptides or if smaller subunits, for example dimers in equilibrium with the oligomeric forms, are involved in the interactions.

In conclusion, we here present structural data that suggest that DNAJB6 unlike many other DNAJ-proteins is able to form a heterogeneous ensemble of oligomers, with a varying number of well-folded DNAJB6 dimers as building blocks. A structural model of a dimer obtained by a combination of homology modeling, crosslinking and docking suggests that a peptide-binding cleft, lined with S/T-residues, is created at the interface between monomers. The structural insights presented here open up new possibilities for targeted mutagenesis and exploration of the dimer-oligomer equilibrium and the interaction sites for peptides, Hsp70 and deacetylases that may regulate its activity and provide a testable DNAJB6 structural model as a frame-work to inform future investigations.

## Methods

### Proteins and reagents

Recombinantly expressed human DNAJB6 (Uniprot # O75190-2) and the Aβ42 peptide was produced as previously described^28^. DNAJB6 protein isotope labeled with ^15^N was obtained by growing the bacterial host in minimal medium containing ^15^N-NH_4_Cl as nitrogen source, and purified as the unlabeled protein. The crosslinking reagents, DSSG (DiSulfoSuccinimidylGlutarate)/BS2G (Bis[Sulfosuccinimidyl]Glutarate) and BS3 (Bis[SulfoSuccinimidyl]Suberate), were purchased from Creative Molecules Inc. (Victoria, Canada) or Thermo Fischer Scientific (Rockford, USA).

### Chemical crosslinking mass spectrometry

#### Chemical crosslinking and protease digestion

The DNAJB6 protein concentration during crosslinking was approximately 60 μM, and if the Aβ42 peptide was present it was at a 1:1 molar ratio of DNAJB6 to peptide. Crosslinkers were dissolved in distilled water to a concentration of 30 mM immediately before use, and added to samples to yield a 3 mM concentration during crosslinking (molar ratio crosslinker to protein approximately 50:1). After 15 min, the crosslinking reaction was quenched by a surplus of primary amine by adding 1 M Tris-(hydroxymethyl)-aminomethane to a final concentration of 20 mM. Samples were subjected to denaturing gel electrophoresis as described in^40^ and the band corresponding to crosslinked DNAJB6 monomer was excised and subjected to in-gel digestion (ratio 1:50 protease to protein) with a combination of trypsin and chymotrypsin (Promega, Madison, WI, USA) at 37 °C. Samples were acidified by adding 2 μl 10% trifluoroacetic acid (TFA). Samples that were not prefractionated by electrophoresis, in order to analyze not only intra-subunit crosslinks, were precipitated with freeze-cold acetone to remove excess reagents and to concentrate the proteins. Pellets were then dissolved in 25 mM ammonium bicarbonate buffer, digested into peptides by in-solution digestion and pretreated on 3 mm long Poros R2 reversed phase microcolumns^59^ before LC-MSMS.

#### Mass spectrometry

Peptides were subjected to reversed-phase nano-LC prior to mass spectrometric analyses in a LTQ-Orbitrap Velos Pro mass spectrometer (Thermo Fisher Scientific, Rockford, USA) equipped with a nanoEasy spray ion source (Proxeon Biosystems, Odense, Denmark), essentially as described in^59^, by data-dependent collision-induced dissociation MS/MS scans excluding 2^+^ since crosslinked peptides generally are charged 3+ or more. For the samples with unlabeled and (^14^N) and labeled (^15^N) peptides the gradient was created by solvent A (0.1% (v/v) FA in water) and solvent B (0.1% (v/v) FA in 100% (v/v) acetonitrile) as follows: 5–30% for 40 min, 30%-50% for 20 min and 50–95% for 5 min and at 95% for 10 min. With MIPS enabled the ^15^N-labeled peptides, as well as hybrid-crosslinked peptides, are discriminated against the non-labeled peptides for fragmentation since they show pre-peaks in the MS-spectra as ^15^N-salts contain some 2% ^14^N, thus the ^15^N-labelled peptides do not fulfill the requirement for fragmentation since A + 2 is not smaller than A + 1, where A is the monoisotopic peak. Therefore to promote MS/MS fragmentation of ^15^N-labeled peptides the mono isotopic precursor selection (MIPS) was disenabled.

#### Data analysis

Raw-files, typically containing 20 000 scans each, from the Thermo Xcalibur software were converted to mgf-files with an in-house licensed version of the software Mascot Server (version 2.5, or earlier, Matrix Science Inc, Boston, USA, http://www.matrixscience.com) and Mascot Distiller (version 2.6, or earlier), in case of the ^14^N-^15^N hybrid crosslinks the settings in MS and MSMS processing were optimized as described in ref.^60^. The mgf-files were analyzed to detect crosslinks with the free MassAI software^61,62^ (http://www.massai.dk, version August 2015; and in case of the ^14^N-^15^N hybrid crosslinks, version February 2017). Filtering was performed to retain only the top 125 most intense peaks per scan, using the built-in MGF-filter. Crosslinked peptides were detected with the following search settings: fragmentation mode: CID, 10 ppm MS accuracy, 0.05 Da or 0.2 MS/MS accuracy, trypsin combined with chymotrypsin as enzyme, 2 allowed missed cleavages, Fixed modification: none. Variable modifications: the crosslinker in question, including deadend variant and/or internal crosslink, 3 allowed modifications per peptide. The searches were performed with the setting ‘Also xlink modified peptides’, without or with the filtering option ‘Crosslink peptide only if peptide is observed as deadend’ which reduces the number of false positives. All MSMS spectra of crosslinks were manually inspected and enclosed in Supplementary Information, and accepted only if following criteria were fulfilled: scan number marked in green (no other explanation to spectra), intensity >1000, fragment ions from both peptides, and no major peaks unexplained. The sequences used in data analysis refer to the amino acid sequence for human DNAJB6 isoform B (UniProt # O75190-2) and for the amino acid sequence of the Aβ42-peptide from human APP (Amyloid Precursor Protein, Uniprot # P05067) the sequence DAEFRHDSGYEVHHQKLVFFAEDVGSNKGAIIGLMVGGVVIA, with and without the extra start methionine that was added during recombinant expression.

### Structural modelling

#### Rosetta modelling for DNAJB6 structural model

To generate structural models of DNAJB6, the sequence was uploaded to several different servers, the LOOPP server (http://clsb.ices.utexas.edu/web/loopp_server.html)^63^, the I-TASSER server (http://zhanglab.ccmb.med.umich.edu/I-TASSER)^64^ and the Robetta server (http://robetta.bakerlab.org)^33^. The data from LOOPP did not cover the whole sequence. I-TASSER and Robetta returned five models, each covering the whole sequence. None of the structural models from I-TASSER matched the crosslinks. The Robetta server is based on a protocol to determine the regions of a protein chain that align to templates and otherwise find regions that will fold into globular units (Output file states: Rosetta provides both ab initio and comparative models of protein domains. Comparative models are built from structures detected and aligned by HHSEARCH, SPARKS, and Raptor. Loop regions are assembled from fragments and optimized to fit the aligned template structures. De novo models are built using the Rosetta denovo protocol and the procedure is fully automated). The server returned several proposed structural models that matched the crosslinks to varying extent. The intra-subunit crosslinks identified by crosslinking mass spectrometry were used as distance constraints to filter the structural models and select the best-fit model. Quality evaluation of the structural model of the DNAJB6 monomer was made by uploading the pdb-file, enclosed in Supplementary Information, to https://swissmodel.expasy.org/qmean/ at the Expasy (Swiss Institute of Bioinformatics). The data show that the model has stereochemical quality comparable to that observed for experimental structures. The QMEAN4 score for the structural model is calculated as a linear combination from 4 statistical potential terms and transformed to a Z-score relating it to high resolution X-ray structures of similar size.

#### NMA simulation of DNAJB6 monomer

Normal mode analysis (NMA) simulations using the elastic network model on DNAJB6 were carried out using the ElNémo software^35^. NMA is used to probe motions in biological macromolecules, assuming that the protein behaves like a harmonic oscillator, from which one can obtain the normal modes and their corresponding frequencies and the majority of the conformations can be captured using the low-frequency normal modes. The structural model of the DNAJB6 monomer (model 5, from the Robetta server) with one C^α^ atom per block and 10 Å interaction distance cut-off criterion was used for the computation. The resulting low-frequency modes 7–11 were analyzed with the help of various softwares. The contact map was computed using the CMView software^65^, the vector representation and the dynamics movie were generated using the software VMD^66^.

#### Crosslink-assisted docking for structural model of DNAJB6 dimer

The inter-subunit crosslinks identified by crosslinking mass spectrometry were used as restraints to generate a structural model of a DNAJB6 dimer by docking. The HADDOCK (High Ambiguity Driven protein-protein DOCKing) program (http://haddock.chem.uu.nl/) is an information-driven flexible docking approach distinguished from ab initio docking methods in that it encodes information on protein interfaces to drive the docking process^67^. Docking was performed using the structural model of the DNAJB6 monomer (Fig. 1, model Robetta_5), with constraints applied for the residues K189 and K232. The interface of the DNAJB6 dimer model was evaluated using the PISA tool (http://www.ebi.ac.uk/pdbe/pisa/)^46^ available for calculations of structural and chemical properties of macromolecular surfaces and interfaces on structures.

### SAXS data collection and analysis

The SAXS data were collected at the Max II beamline I911-SAXS^68^ at the MAX IV laboratory. The data was initially reduced and processed using an automatic pipeline of scripts developed at MAX-Lab and using the ATSAS tools^69,70^. Data were normalized to the intensity of the transmitted beam and buffer scattering was subtracted. Forward scattering I(0) and the radius of gyration Rg were estimated using the Guinier approximation^71^. Using Primus, we calculated the distance distribution functions P(R) of the scattering patterns and estimated the maximum particle dimensions Dmax. Primus also automatically calculates the excluded volume of the hydrated particle (Porod volume Vp)^70^ together with the distance distribution function. All data sets were modelled with DAMMIF and the ab initio model’s excluded volume Va was divided by two in order to estimate the mass^70^. We performed online size exclusion chromatography on DNAJB6 using an ÄKTA Pure (GE Healthcare, Uppsala, Sweden) FPLC. The sample, 250 μl 6.5 mg/ml DNAJB6, was applied to a Superdex 200 Increase 10/300 (GE Healthcare, Uppsala, Sweden) equilibrated with 20 mM phosphate buffer pH 8.0 and 150 mM NaCl. Flow rate was 0.2 ml/min and data acquired every minute.

### Electron microscopy and single particle averaging

#### Gradient fixation

The polydispersity of the DNAJB6 oligomers prevented single-particle reconstruction on the samples earlier analyzed by negative stain EM^26^ hence in order to improve the specimen for EM studies we here fractionated the DNAJB6 oligomers using the GraFix procedure to reduce the sample complexity by a gradient fixation protocol^72,73^, in which the sample is subjected to weak intramolecular chemical crosslinking during density gradient ultracentrifugation. A 4 ml linear 5–30% (w/w) sucrose and 0–0.2% (w/w) glutaraldehyde gradient in buffer (20 mM NaPO4, 150 mM NaCl, pH 8.0) was prepared using a gradient master (Biocomp, Canada) mixer. In total, 200 µl of DNAJB6 solution (0.4 mg/ml) was added on top of the gradient and run for 16 h at 4 °C at a speed of 30,000 rpm in a Beckman SW 55 rotor. Fractions of approx. 250 µl were collected from the bottom of the tube using a fraction collector (Gilson, USA) and analyzed by blue native-PAGE on precast 4–16% Bis-Tris Gels (Life Technologies, Sweden) and scanned with an Epson Perfection V600 photo scanner.

#### Sample preparation and imaging

Samples (5 µl of each) from fractions 1–6 after GraFix were applied to glow-discharged continuous carbon-coated 400 mesh copper grids. The grids were subsequently blotted with filter paper, washed with two drops of milli-Q water and negatively stained with 2% (w/v) uranyl acetate. Fraction 5 obtained well-defined particles at relatively high concentration (~100 particles/image) and was selected for single-particle analysis. The sample was imaged using a JEM2100F electron microscope (JEOL, Japan) operated at 200 kV. Images were recorded on a DE-20 direct electron detector (Direct Electron, USA) at a magnification of ×30,000 and 0.9–1.7 µm defocus. The selected magnification resulted in a pixel size of 2.08 Å at the specimen level. Images were recorded using a frame rate of 20 frames/s and 2 s exposure time. The accumulated dose for the whole exposure was approximately 20 e^−^/Å^2^. A total of 40 images were recorded.

#### Image processing

For each exposure, the comprised frames were drift corrected using the DE_process_frames-2.7.1.py script^74^. The first five frames were excluded from the data due to large amounts of drift. The remaining frames (6–40) were processed using full frame alignment. The alignment procedure was iterated four times and the resulting drift-corrected images were imported to EMAN2 (version 2.12) for further processing^75^. First, defocus, particle separation and contrast were evaluated with *e2evalimage.py*. Two images were discarded due to high defocus values. Then, 4 344 particles were selected automatically from the remaining 38 images using *e2boxer.py* in *swarm* mode. False positives from the automated picking procedure, representing clusters of aggregated particles or staining artefacts, were discarded. For each image, the contrast transfer function (CTF) parameters were estimated using 160 × 160 pixels boxed out (particle containing) regions using *e2ctf.py*. Reference-free two-dimensional (2D) classification was performed using 3 717 phase-flipped particles with *e2refine2d.py*. 2D classes representing different orientations were selected for initial model generation using *e2initialmodel.py*, assuming asymmetric particles (*sym* = C1). The model with the best matching 2D class averages and model projections was selected as a starting model for three-dimensional (3D) refinement. First, a 3D refinement was performed with downscaled (4.16 Å/pixel) data aiming at low resolution (*targetres* = 25 Å) using *e2refine_easy.py*. A second round of 3D refinement was performed using the full-size (2.08 Å/pixel) data and the final map from the first 3D refinement as input, targeting medium resolution (*targetres* = 20 Å). The resolution reported was calculated at Fourier shell correlation (FSC) = 0.143^76^, following the gold-standard FSC procedure^77^ embedded in EMAN2. The presence of two-fold symmetry was tested by imposing this symmetry element along a chosen axis. Subsequently, reconstruction of a 3D map was made by applying C2 symmetry. Molecular graphics and analyses were performed with the UCSF Chimera package^78^ (http://www.rbvi.ucsf.edu/chimera).

### Data availability

Datasets generated and analysed during the current study, which are not already included in this published article and its Supplementary Information files, are available from the corresponding author on reasonable request.

## Electronic supplementary material

Supplementary video

Supplementary information

DNAJB6 monomer model

DNAJB6 dimer model

dnajb6_negstain
