# Supplementary material for: Structural modelling of the DNAJB6 oligomeric chaperone shows a peptide-binding cleft lined with conserved S/T-residues at the dimer interface
Source: Sci Rep. 2018 Mar 26;8:5199. doi: 10.1038/s41598-018-23035-9 (PMC5979959; doi:10.1038/s41598-018-23035-9)
Supplement: Supplementary file 2 — Supplementary information [file 41598_2018_23035_MOESM2_ESM.pdf]

Structural modelling of the DNAJB6 oligomeric chaperone shows a peptide-binding cleft lined with conserved S/T-residues at the dimer interface

[1]Christopher AG Söderberg*, [2]Cecilia Månsson, [2]Katja Bernfur, [2]Gudrun Rutsdottir, [3]Johan Härmark, [4]Sreekanth Rajan, [2]Salam Al-Karadaghi, [5]Morten Rasmussen, [5]Peter Höjrup, [3]Hans Hebert, [2]Cecilia Emanuelsson* [1]MAX IV Laboratory, Lund University, PO Box 118, SE-221 00 Lund, Sweden; [2]Department of Biochemistry and Structural Biology, Center for Molecular Protein Science, Lund University, PO Box 124, SE-221 00 Lund, Sweden; [3]School of Technology and Health, KTH Royal Institute of Technology and Department of Biosciences and Nutrition, Karolinska Institute, Stockholm, Sweden; [4]School of Biological Sciences, Nanyang Technological University, Singapore - 637551, Singapore; [5]Department of Biochemistry and Molecular Biology, University of Southern Denmark, Odense, Denmark.
* Correspondence to: Cecilia.Emanuelsson@biochemistry.lu.se

Supplemental information

Fig. S1 Quality evaluation of the structural model of the DNAJB6 monomer. Related to Fig. 1
Fig. S2. The subgroup of DNAJB6-like homologues. Related to Fig. 4
Fig. S3 Fourier shell correlation between reconstructions. Related to Fig. 6
Fig. S4 Fitting the structural model of the DNAJ6 dimer into a DNAJB6 oligomer model. Related to Fig. 7
Fig. S5 MSMS-spectra for crosslinks within DNAJB6 monomers. Related to Table 1
Fig. S6 MSMS-spectra for crosslinks between DNAJB6 monomers and between DNAJB6 and Aβ42. Related to Table 2
Fig. S7 MSMS spectra confirming K189-K189 and K232-K232 as ^{14}N-^{15}N hybrid crosslinks. Related to Table 2

Supplemental Information files:

SI_1 DNAJB6 monomer model_Robetta_5. Related to Fig. 1
SI_2 mode-9-movie. Related to Fig. 2
SI_3 DNAJB6 dimer model_Haddock. Related to Fig. 3
SI_4 dnajb6_negstain_c2.mrc. Related to Fig. 6 and 7.

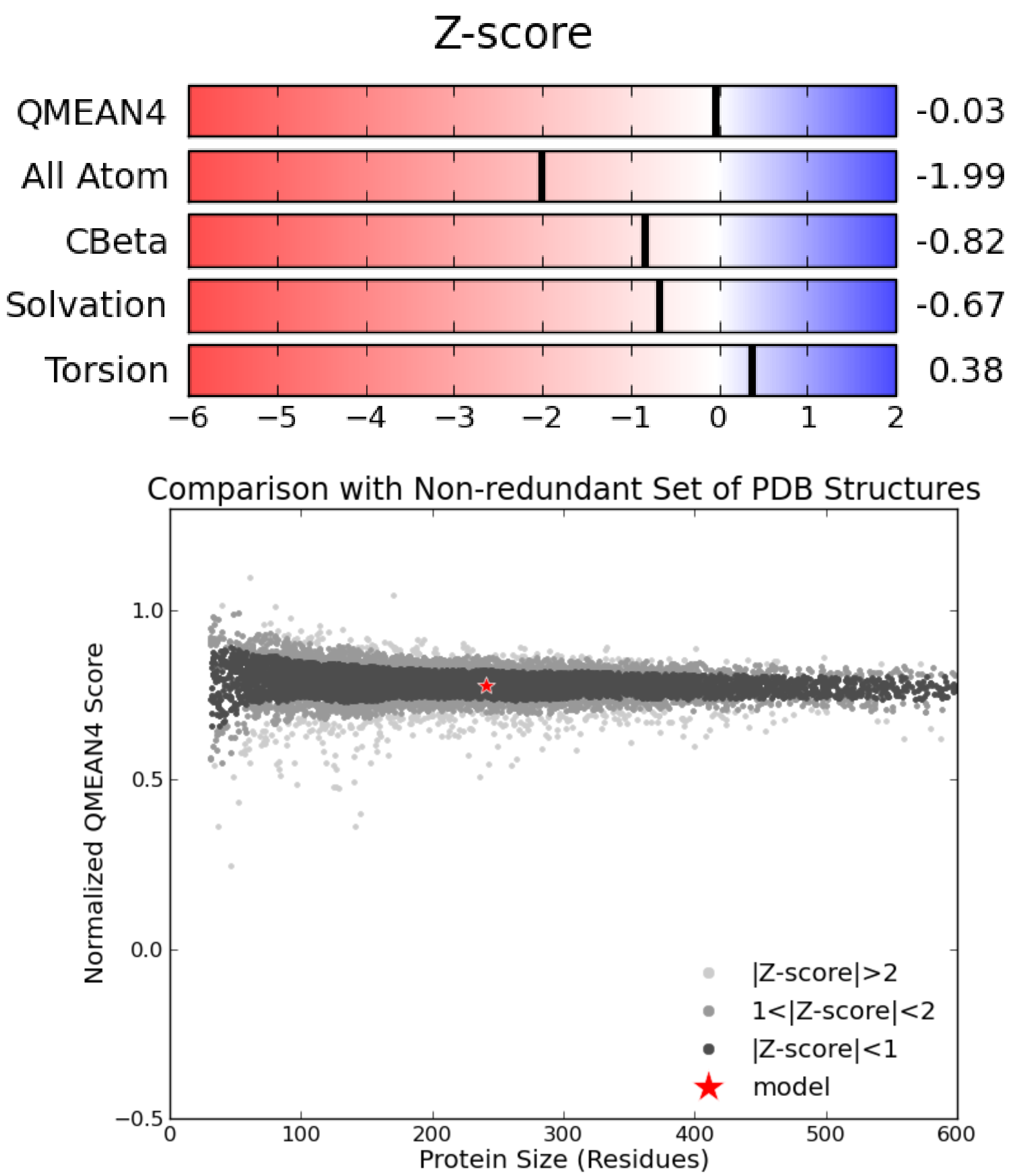

Fig. S1 Quality evaluation of the structural model of the DNAJB6 monomer. QMEAN4 score for the structural model of the DNAJB6 is calculated as a linear combination from 4 statistical potential terms and transformed to a Z-score relating it to high resolution X-ray structures of similar size. The data show that the model has stereochemical quality comparable to that observed for experimental structures. The file DNAJB6 monomer model_Robetta_5.pdb was uploaded to https://swissmodel.expasy.org/qmean/

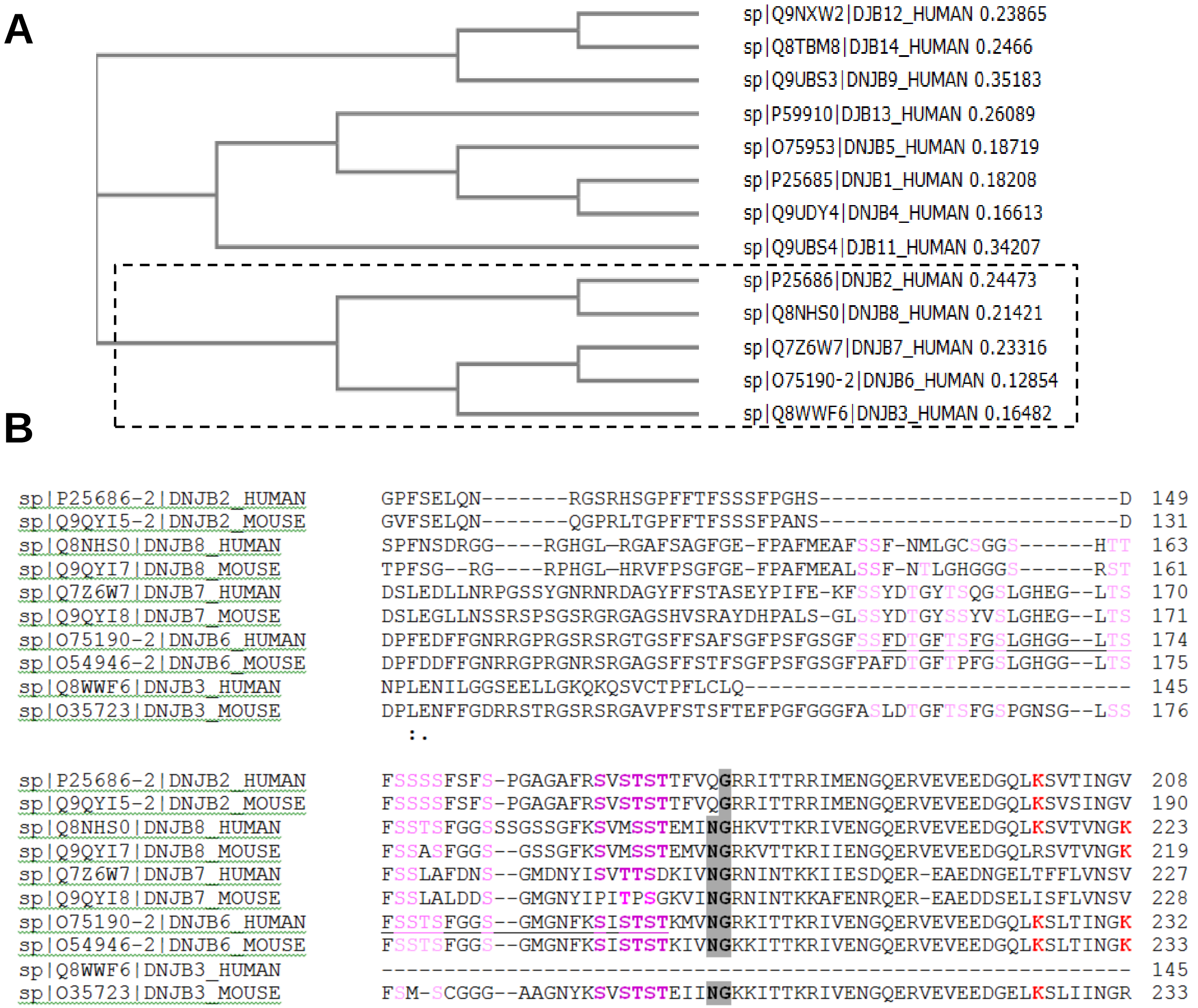

Fig. S2 The subgroup of DNAJB6-like homologues. (A) Phylogenetic tree data based on multiple alignment of full length amino acid sequences, showing that DNAJB6 belongs to a subgroup of human DNAJB-homologues (enboxed with dashed line). (B) Partial sequence alignment of the subgroup of human DNAJB6-like homologues (DNAJB2, 3, 6-8); the serine/threonine (S/T)-rich region in DNAJB6 (aa 155-195) is underlined, S-STST aa 190, 192-195 (dark pink); other ST-residues in region aa 155-195 (light pink). Other high-lighted residues are the residues N199 and G200 (grey-shaded) in the mobile loop shown in Fig. 2 and the K225/K232-residues (red) suggested to regulate DNAJB6/DNAJB8 activity through deacetylation.

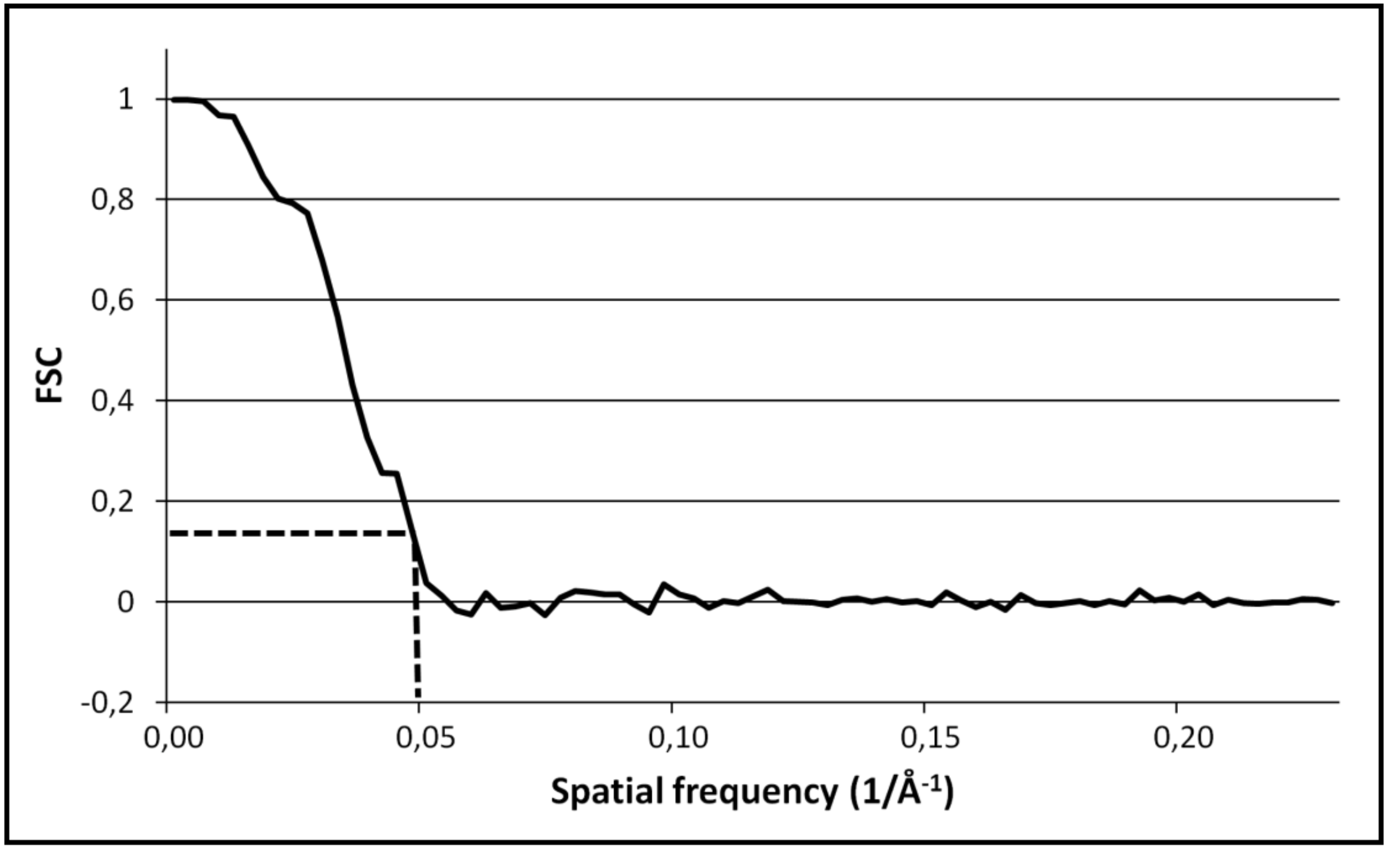

Fig. S3 Fourier shell correlation between reconstructions produced by splitting the data set in two halves.
Both sets were reconstructed separately. The resolution reported for the reconstructed 3D map was calculated at FSC = 0.143.

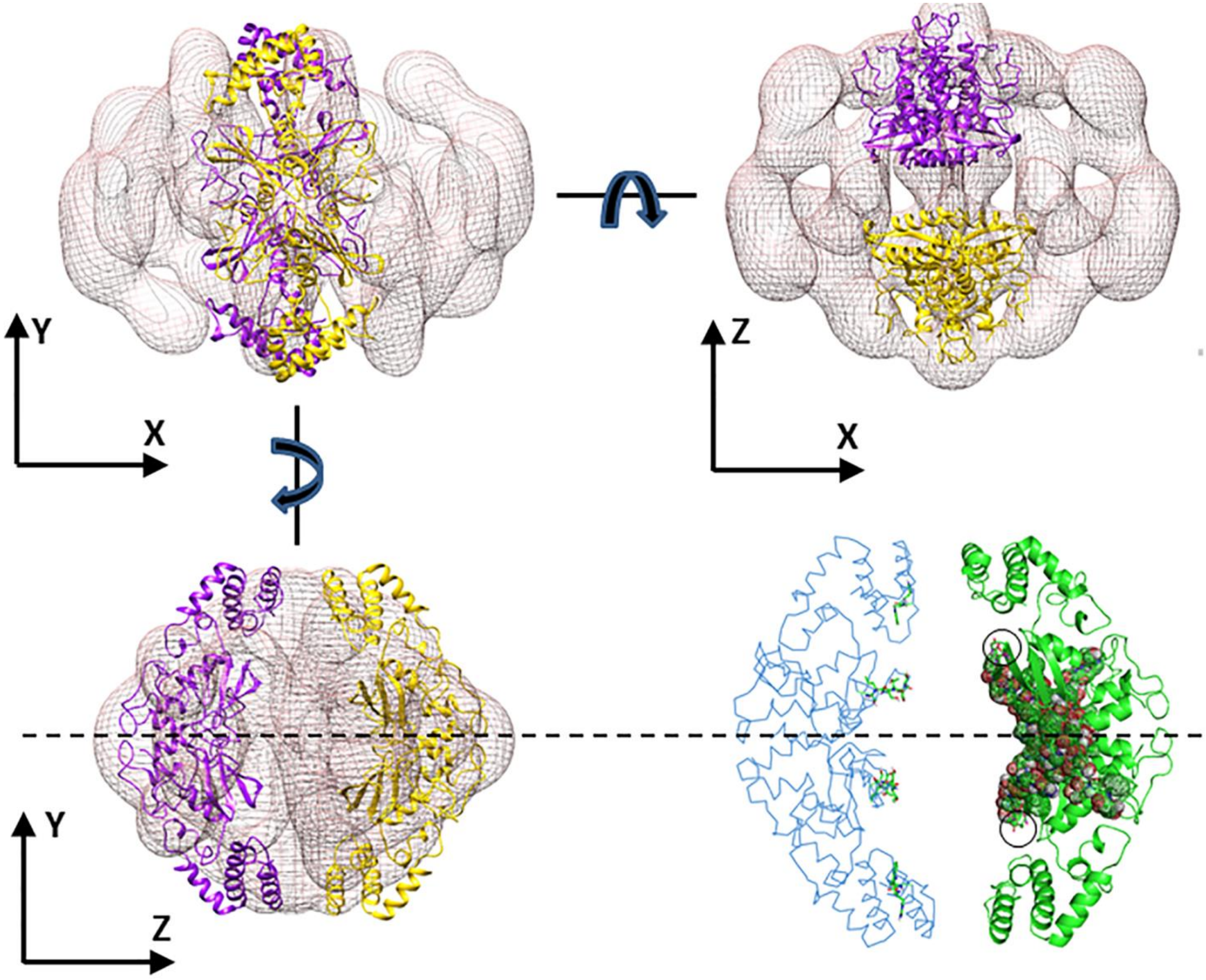

Fig. S4 Fitting the DNAJB6 dimer model into DNAJB6 oligomer. Two DNAJB6 dimers shown in cartoon were fitted to the map using the Fit in Map feature in Chimera. The DNAJB6 density map is shown with partially transparent mesh surface representation from three perpendicular directions showing the 2 fold symmetry axis (dashed line). In the lower right corner is shown, as an orientation: the left dimer in Cα trace with the following amino acids as sticks: the C-terminal domain residues S-STST (190, 192-196) and the N-terminal domain residues HPD (30-32) and the right dimer in cartoon with the residues in the S/T-rich region (155-195) as spheres and as sticks the residues N199 and G200 (encircled) in the mobile loop region.

Fig. S5 MSMS-spectra for crosslinks within DNAJB6 monomers. Related to Table 1.

Color coding in MSMS spectra:
y-ions (blue), b-ions (green), peptide A/B ions (purple), unexplained (red).

Peptide with longest sequence is referred to as A peptide.

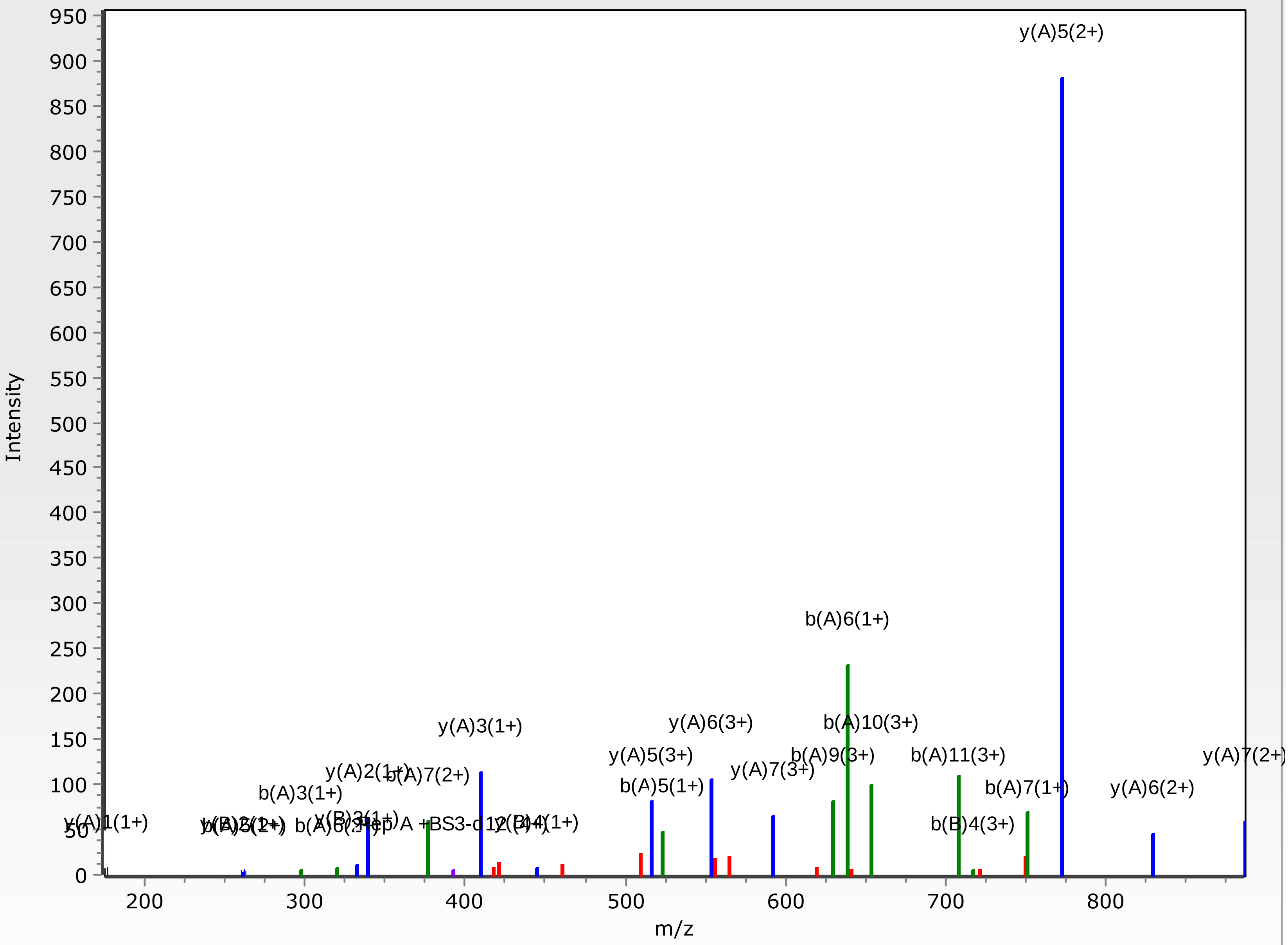

DNAJB6 K20-K25, HASPEDIKKAYR + KLALK

Fig. S5 MSMS-spectra for crosslinks in Table 1 - crosslinked DNAJB6 monomers

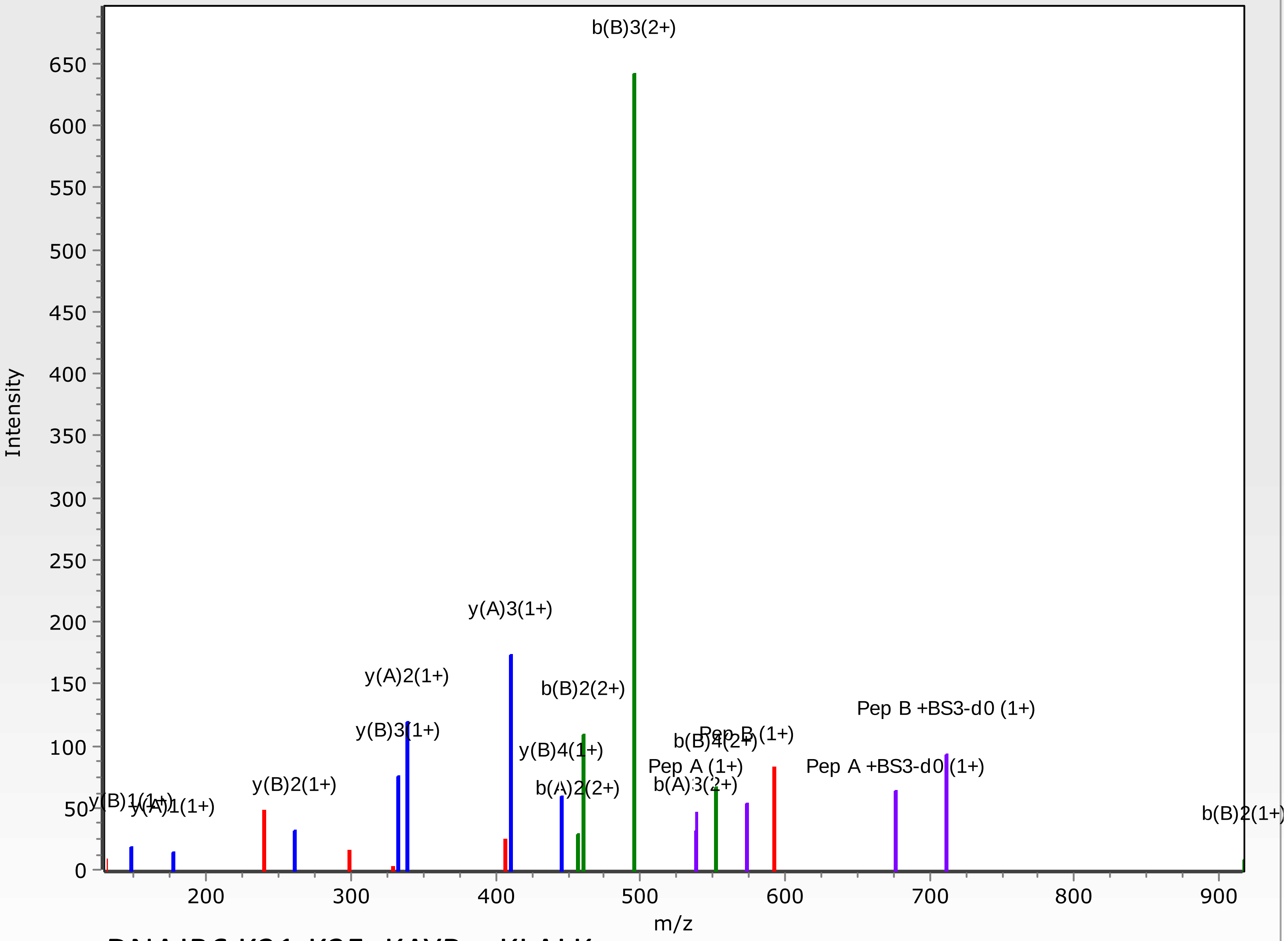

DNAJB6 K21-K25, KAYR + KLALK

Fig. S5 MSMS-spectra for crosslinks in Table 1 - crosslinked DNAJB6 monomers

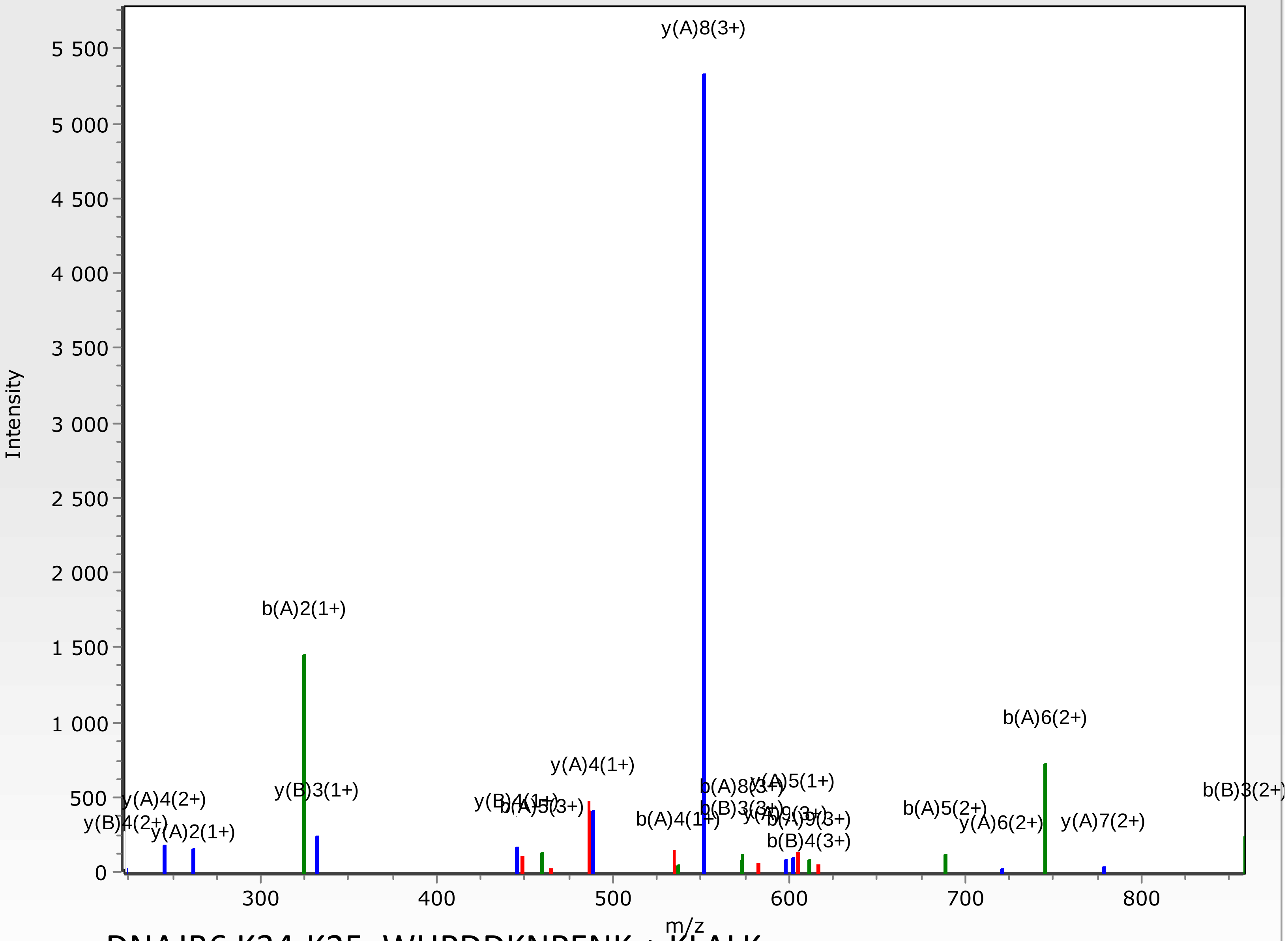

DNAJB6 K34-K25, WHPDDKNPENK + KLALK

Fig. S5 MSMS-spectra for crosslinks in Table 1 - crosslinked DNAJB6 monomers

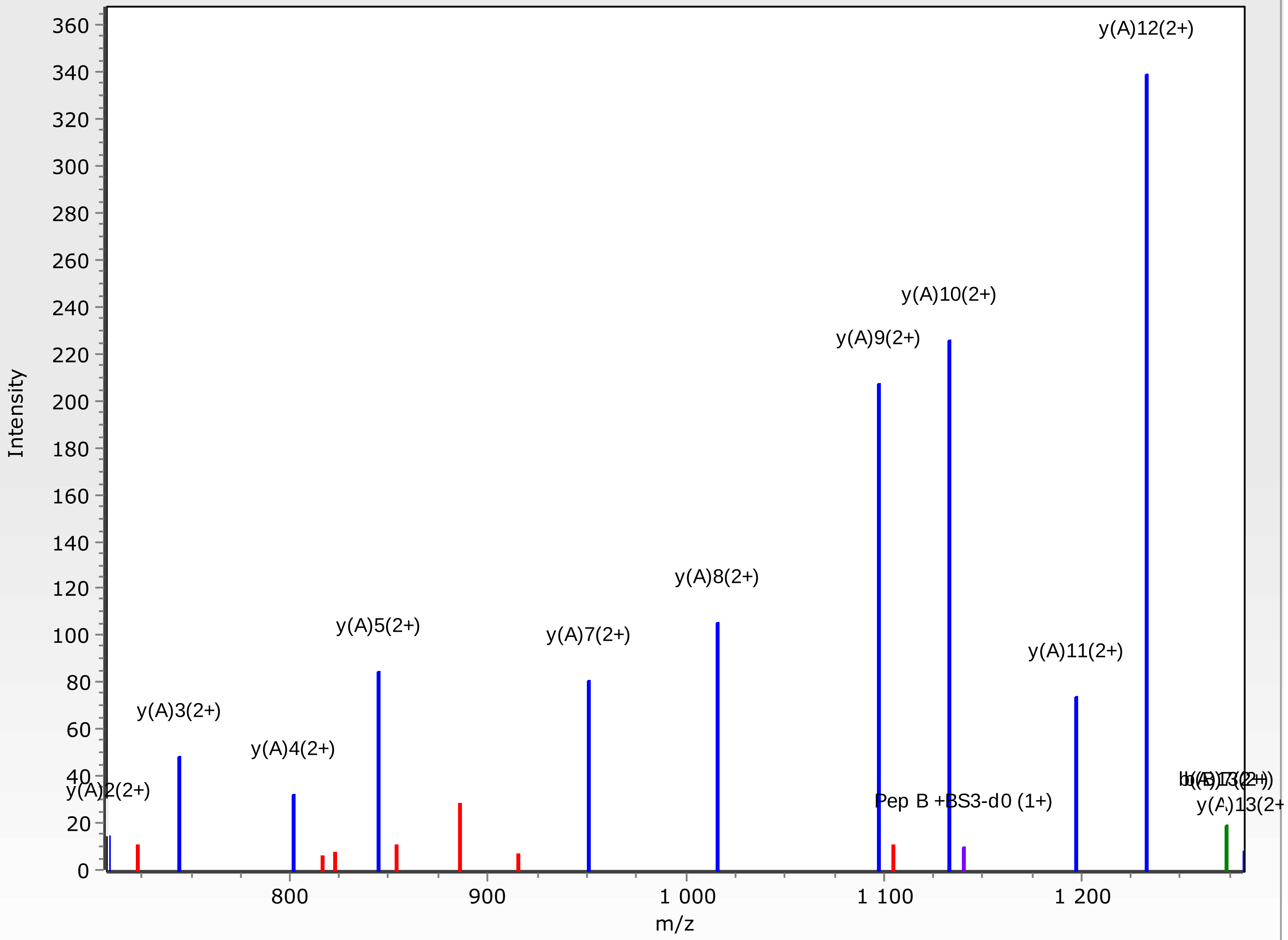

DNAJB6 K60-K67, QVAEAYEVLSDAKK + DIYDKYGK

Fig. S5 MSMS-spectra for crosslinks in Table 1 - crosslinked DNAJB6 monomers

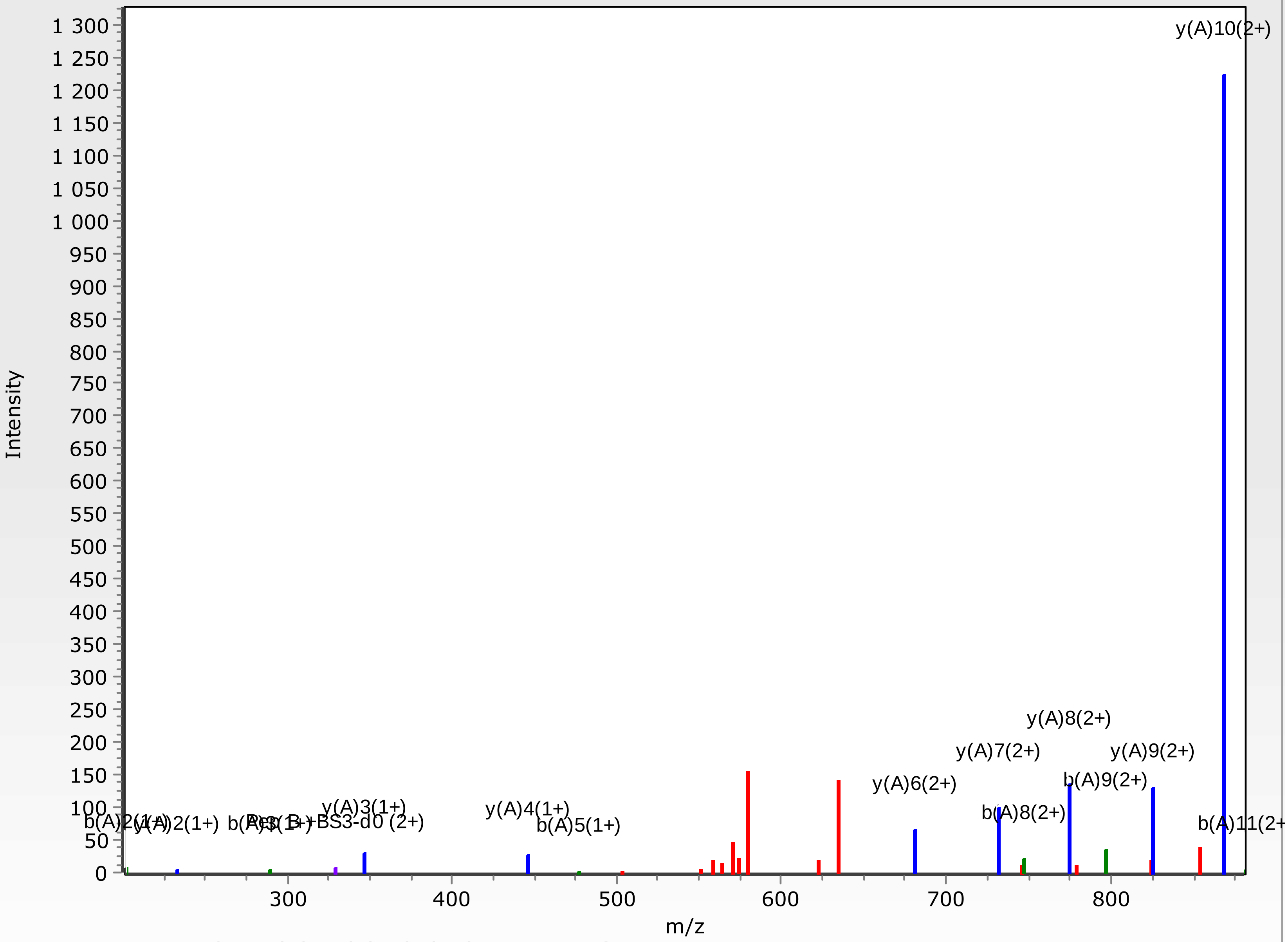

DNAJB6 K196-K29, SISTSTKMVNGR +ALKW

Fig. S5 MSMS-spectra for crosslinks in Table 1 - crosslinked DNAJB6 monomers

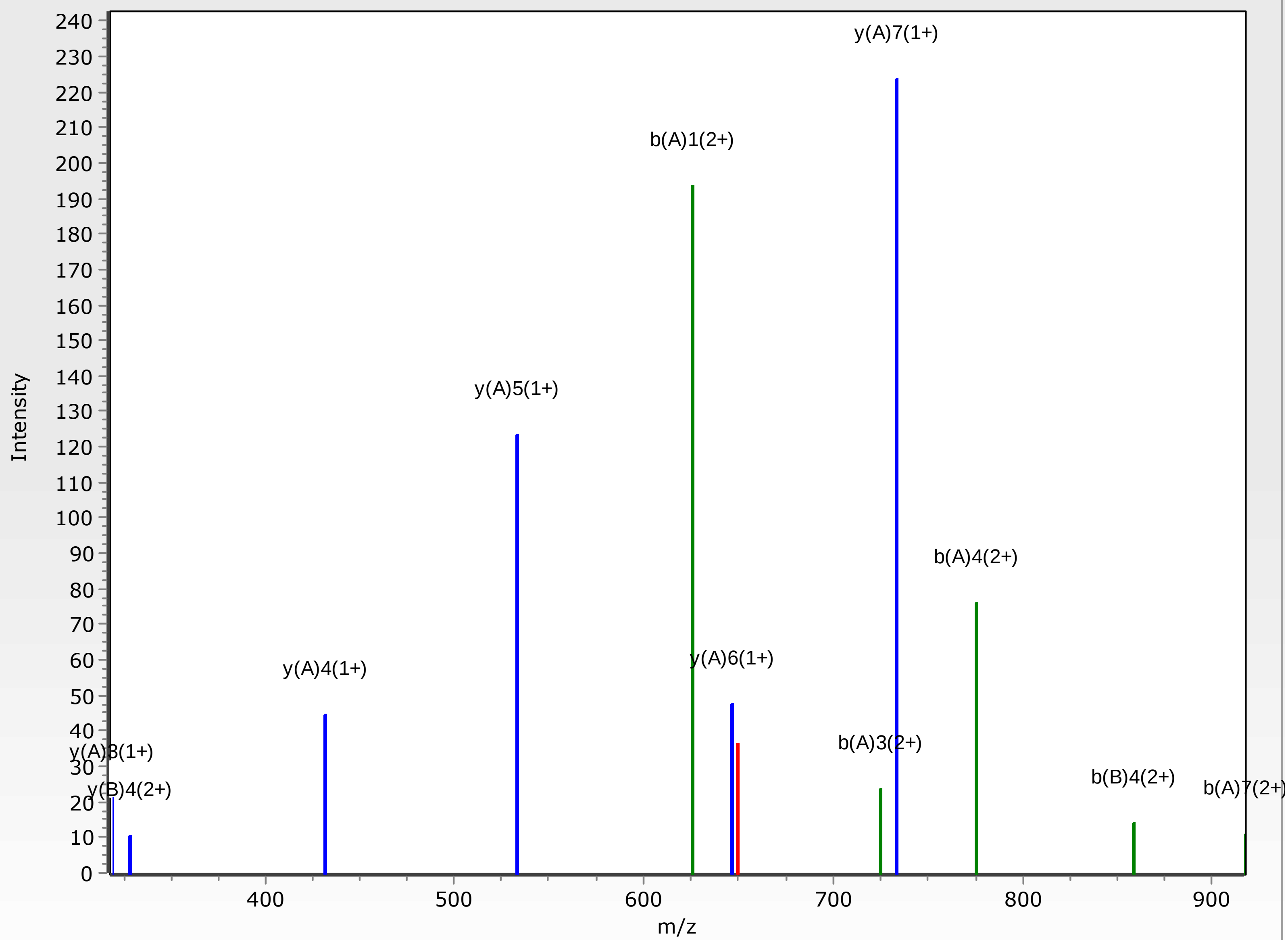

DNAJB6 K225-K21, KSLTINGK + KAYRK

Fig. S5 MSMS-spectra for crosslinks in Table 1 - crosslinked DNAJB6 monomers

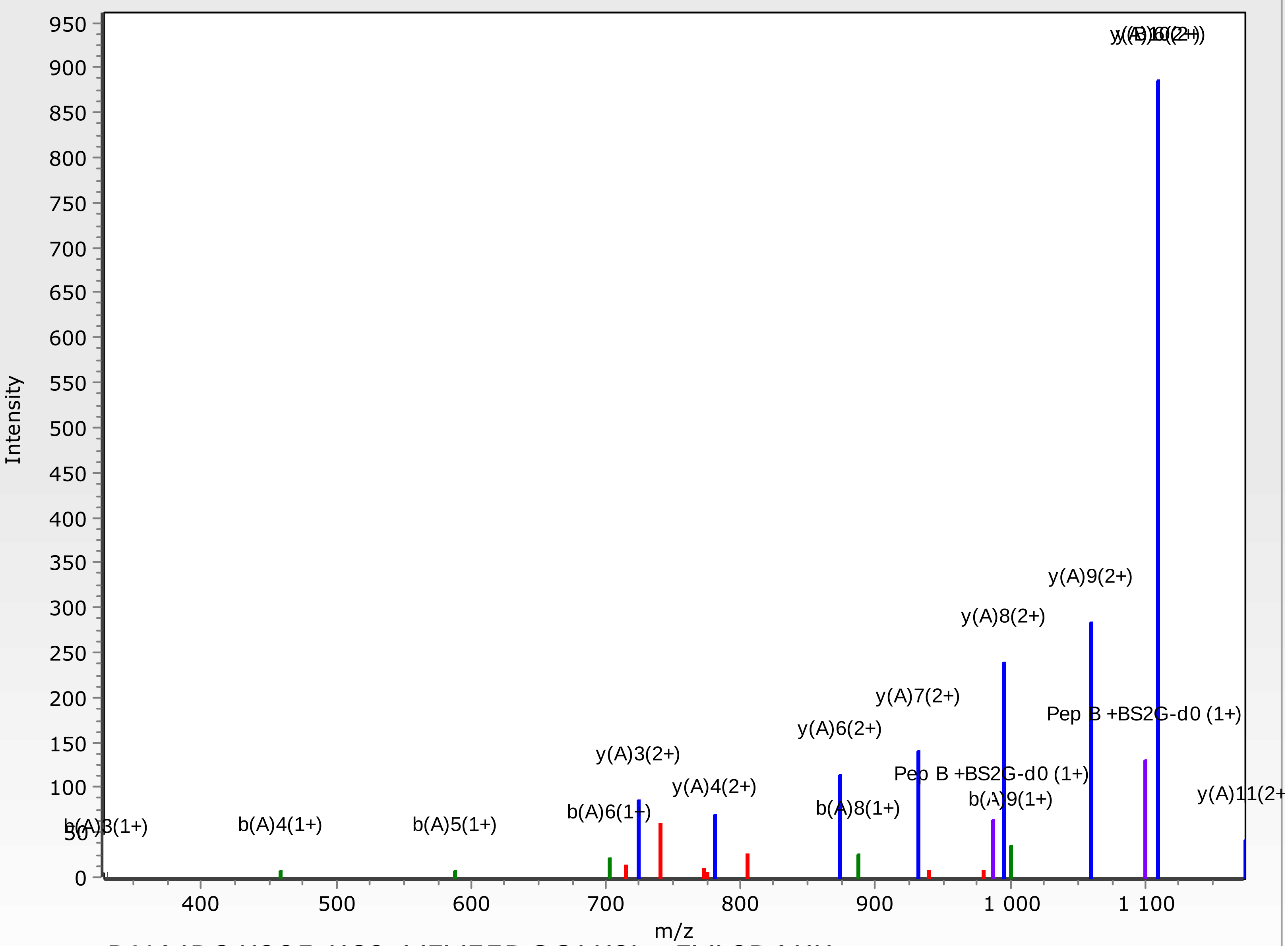

DNAJB6 K225-K60, VEVEEDGQLKSL+ EVLSDAKK

Fig. S5 MSMS-spectra for crosslinks in Table 1 - crosslinked DNAJB6 monomers

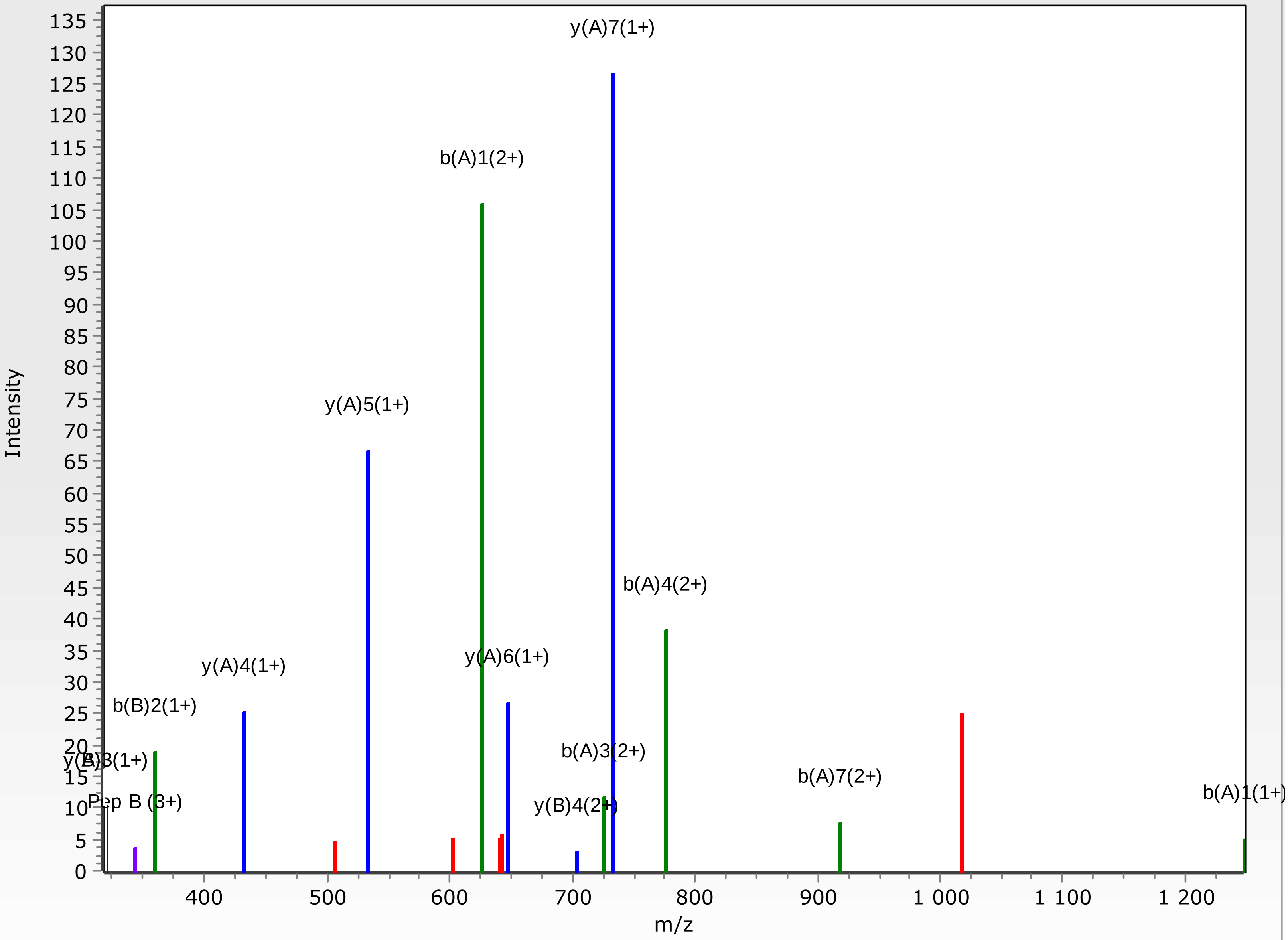

DNAJB6 K225-K70, KSLTINGK + DKYGKEGL

Fig. S5 MSMS-spectra for crosslinks in Table 1 - crosslinked DNAJB6 monomers

Fig. S6 MSMS-spectra for crosslinks within DNAJB6 and between DNAJB6 and Aβ42. Related to Table 2.

Color coding in MSMS spectra:
y-ions (blue), b-ions (green), peptide A/B ions (purple), unexplained (red).

Peptide with longest sequence is referred to as A peptide.

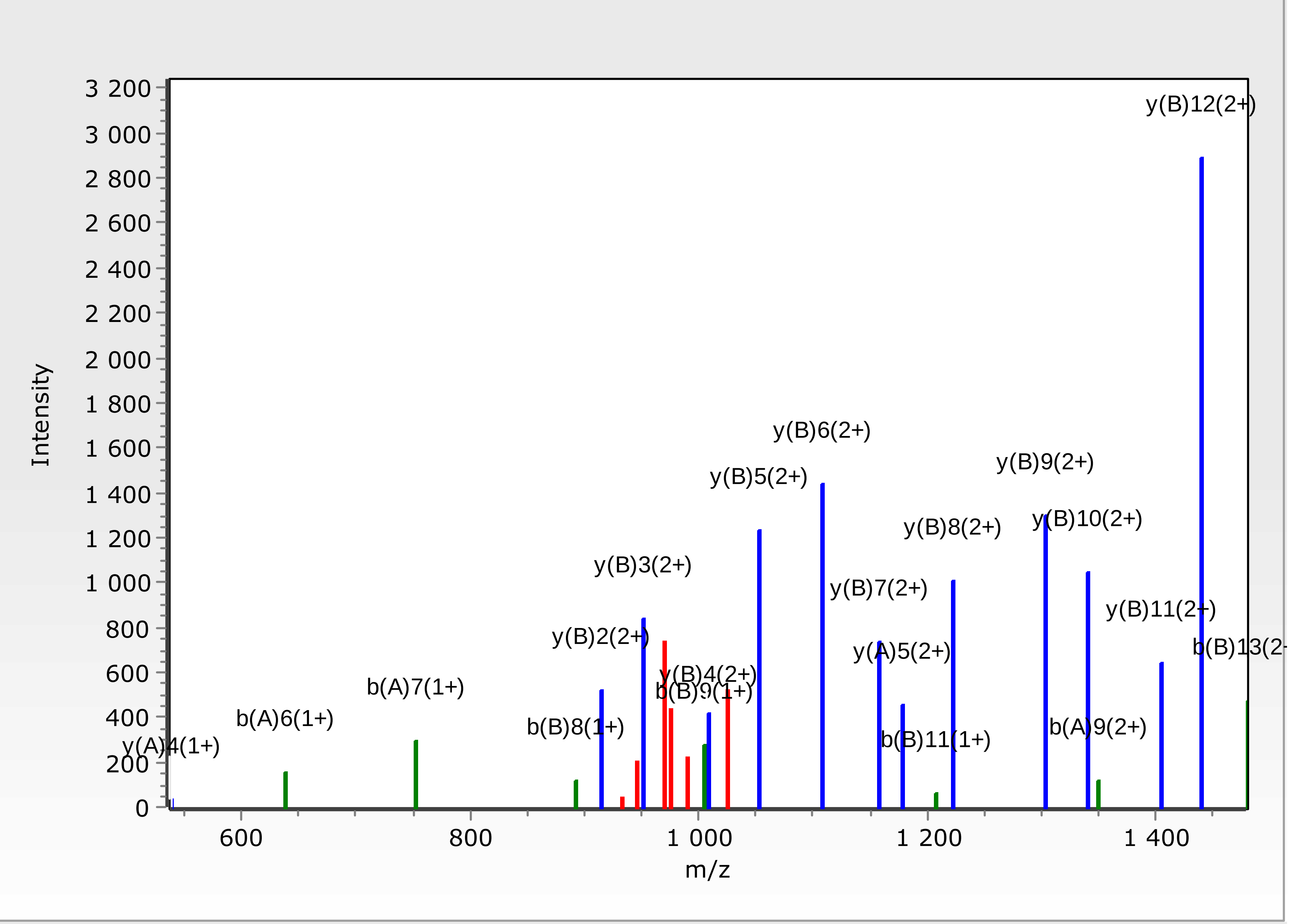

DNAJB6 K20-K60, HASPEDIKKAYR + QVAEAYEVLSDAKK

Fig. S6 MSMS-spectra for Table 2

Dataset #1
Duplicate samples with crosslinked DNAJB6 oligomers

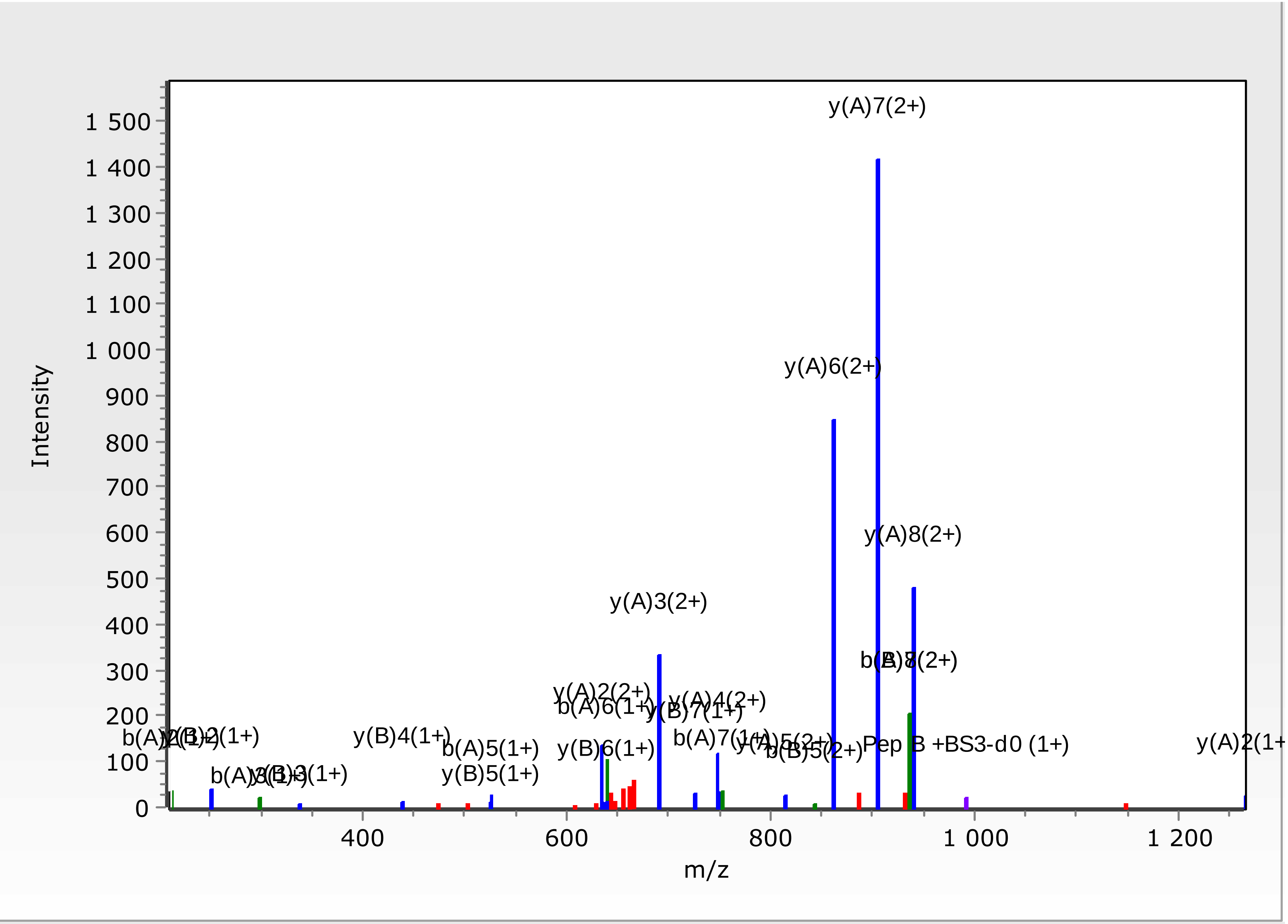

DNAJB6 K20-K189, HASPEDIKK + KSISTSTK

Fig. S6 MSMS-spectra for Table 2

Dataset #1
Duplicate samples with crosslinked DNAJB6 oligomers

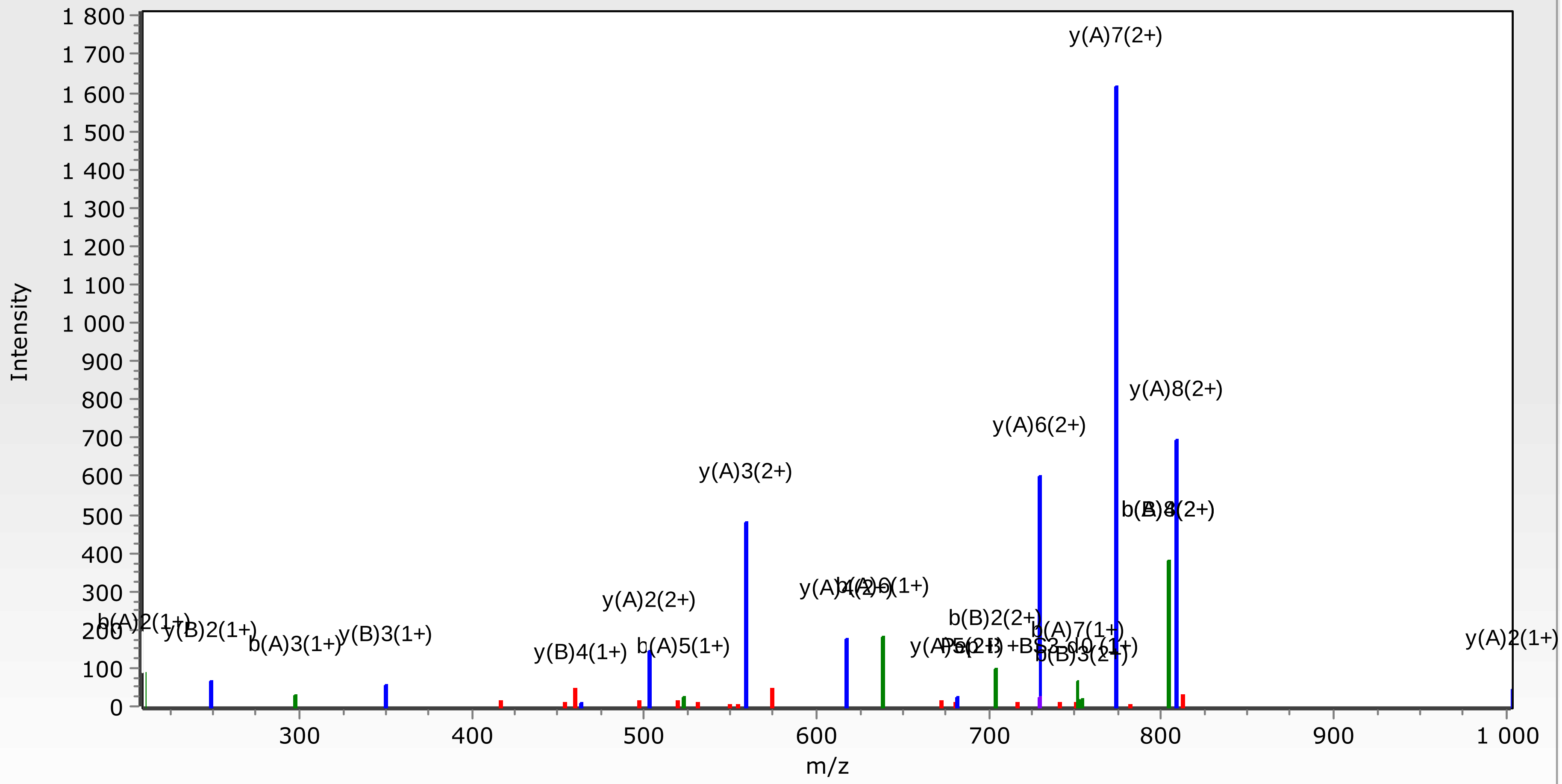

DNAJB6 K20-K202, HASPEDIKK + KITTK

Fig. S6 MSMS-spectra for Table 2

Dataset #1

Duplicate samples with crosslinked DNAJB6 oligomers

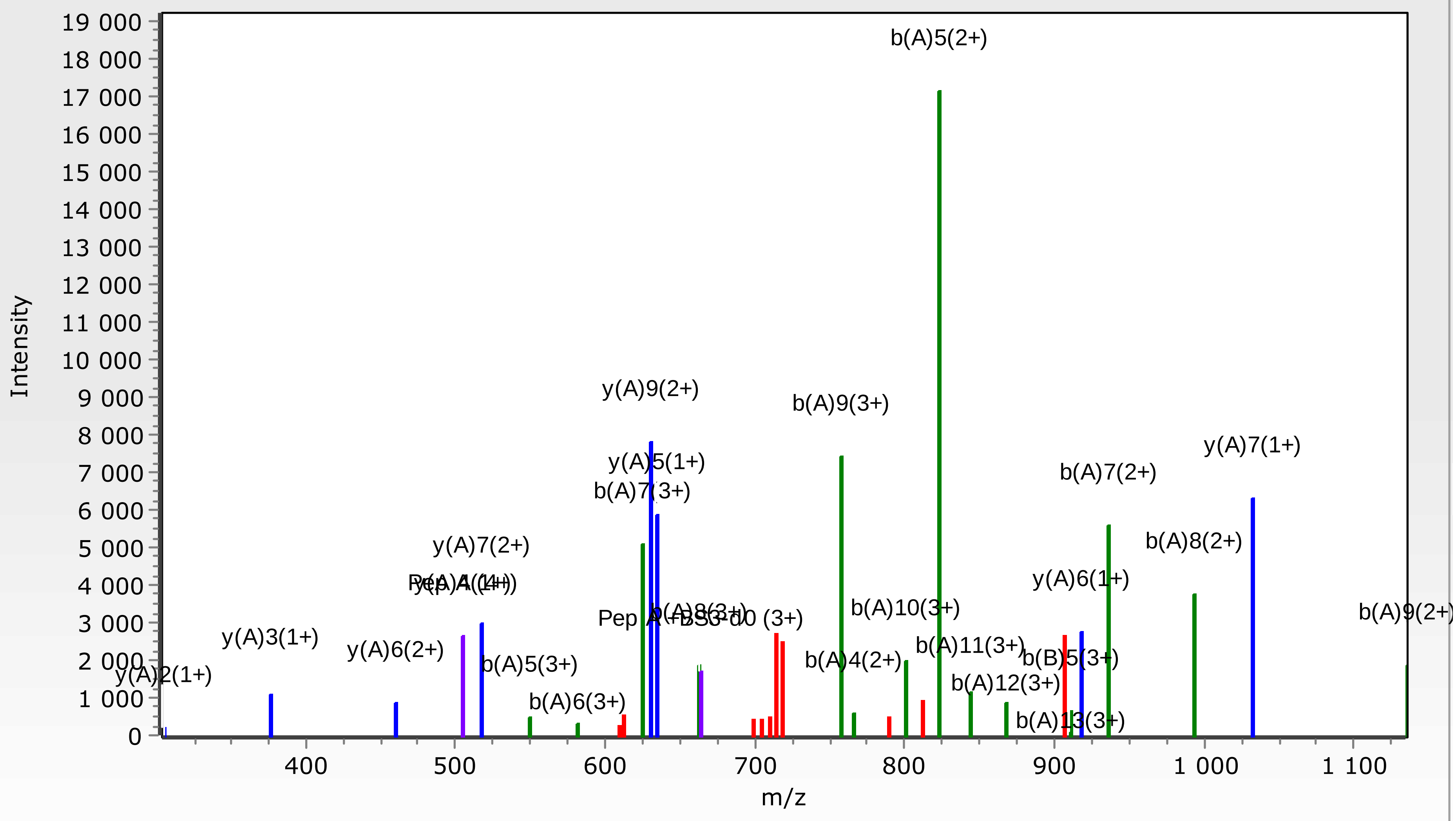

DNAJB6 K34-K202, HPDKNPENKEEAER + KITTKR

Fig. S6 MSMS-spectra for Table 2

Dataset #1
Duplicate samples with crosslinked DNAJB6 oligomers

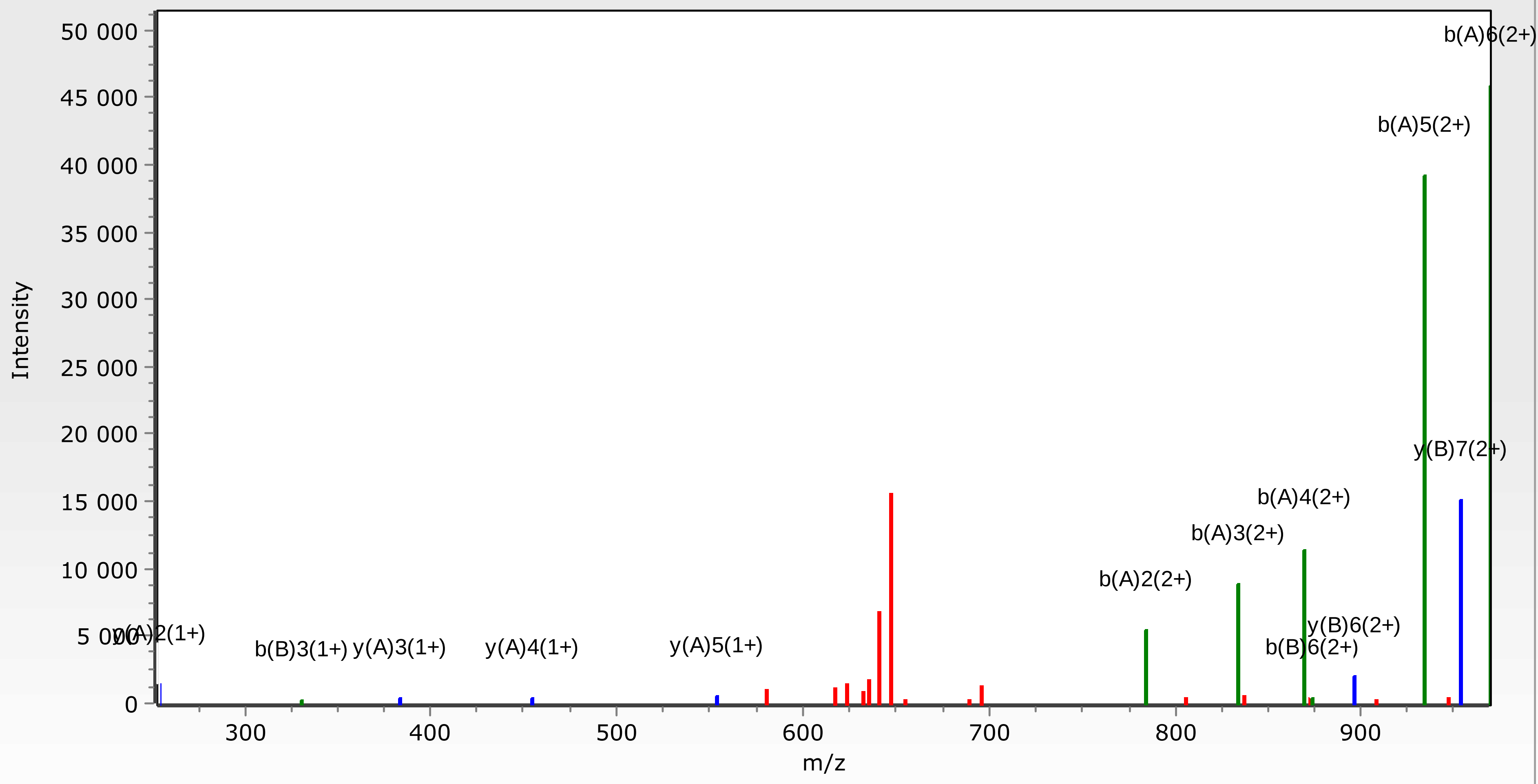

DNAJB6 K47-K232, KQVAEAY + TINGKEQLL

Fig. S6 MSMS-spectra for Table 2

Dataset #1
Duplicate samples with crosslinked DNAJB6 oligomers

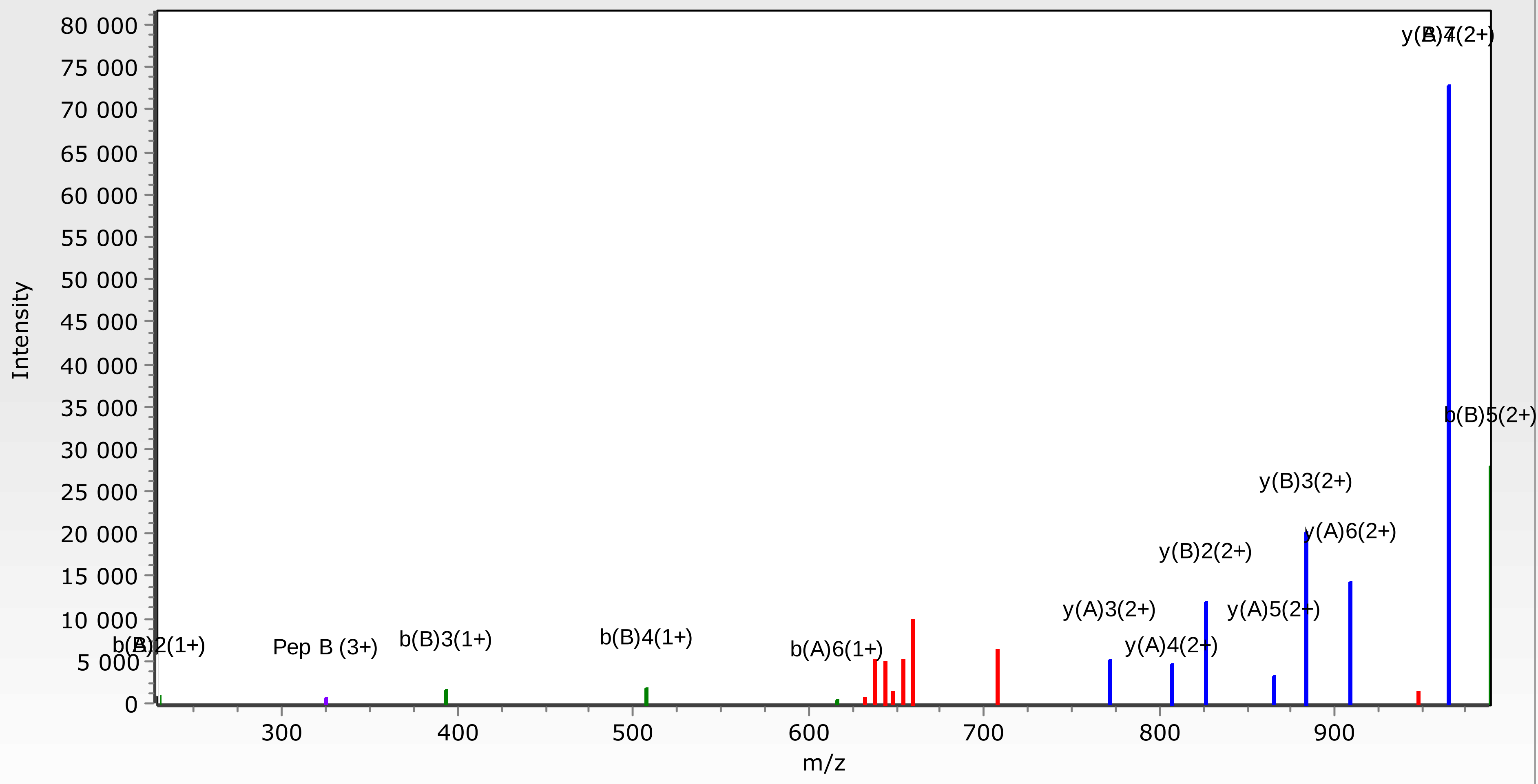

DNAJB6 K60-K67, EVLSDAKKR + DIYDKY

Fig. S6 MSMS-spectra for Table 2

Dataset #1
Duplicate samples with crosslinked DNAJB6 oligomers

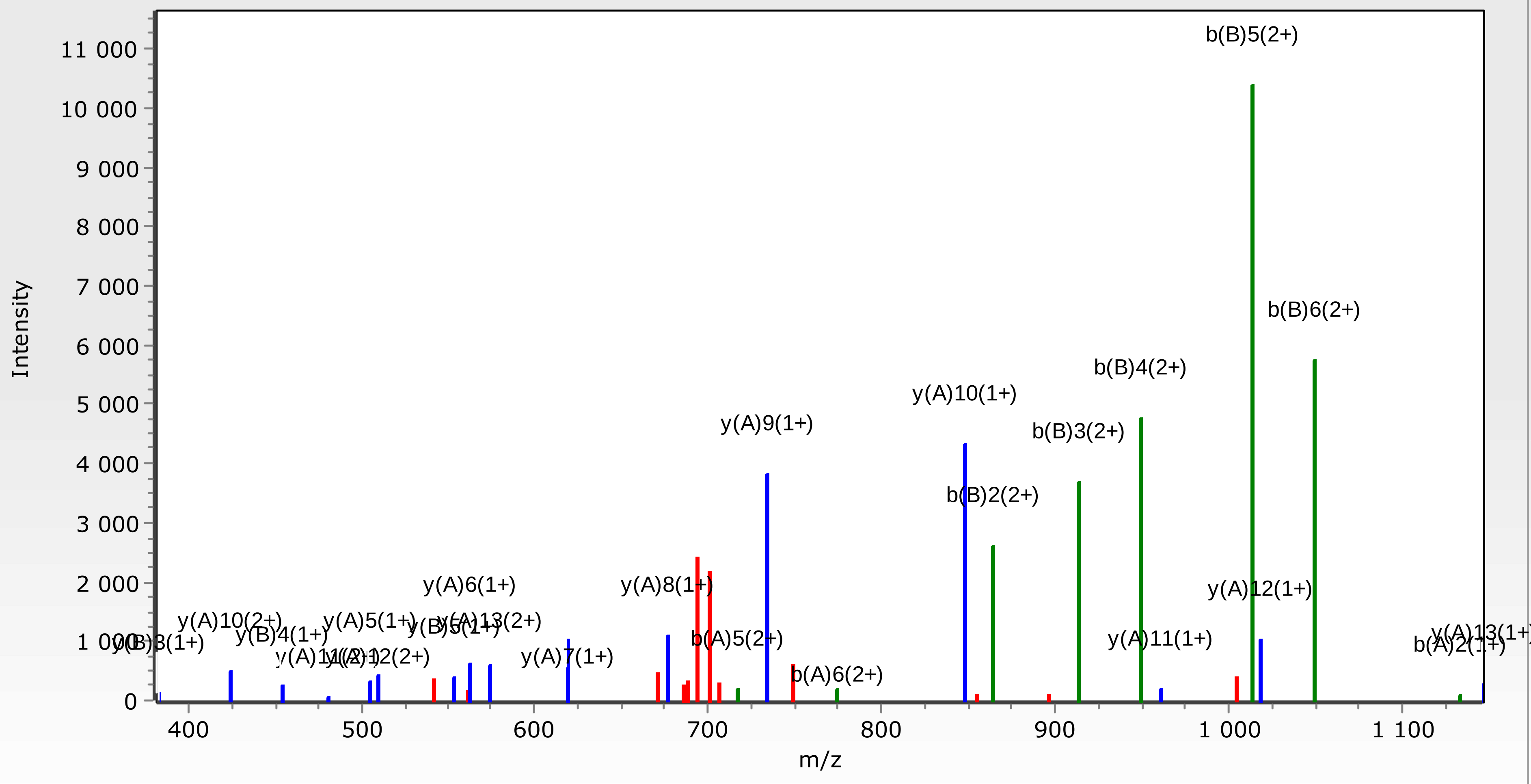

DNAJB6 K70-K47, GKEGLNGGGGGGSHF + QVAEAY

Fig. S6 MSMS-spectra for Table 2

Dataset #1
Duplicate samples with crosslinked DNAJB6 oligomers

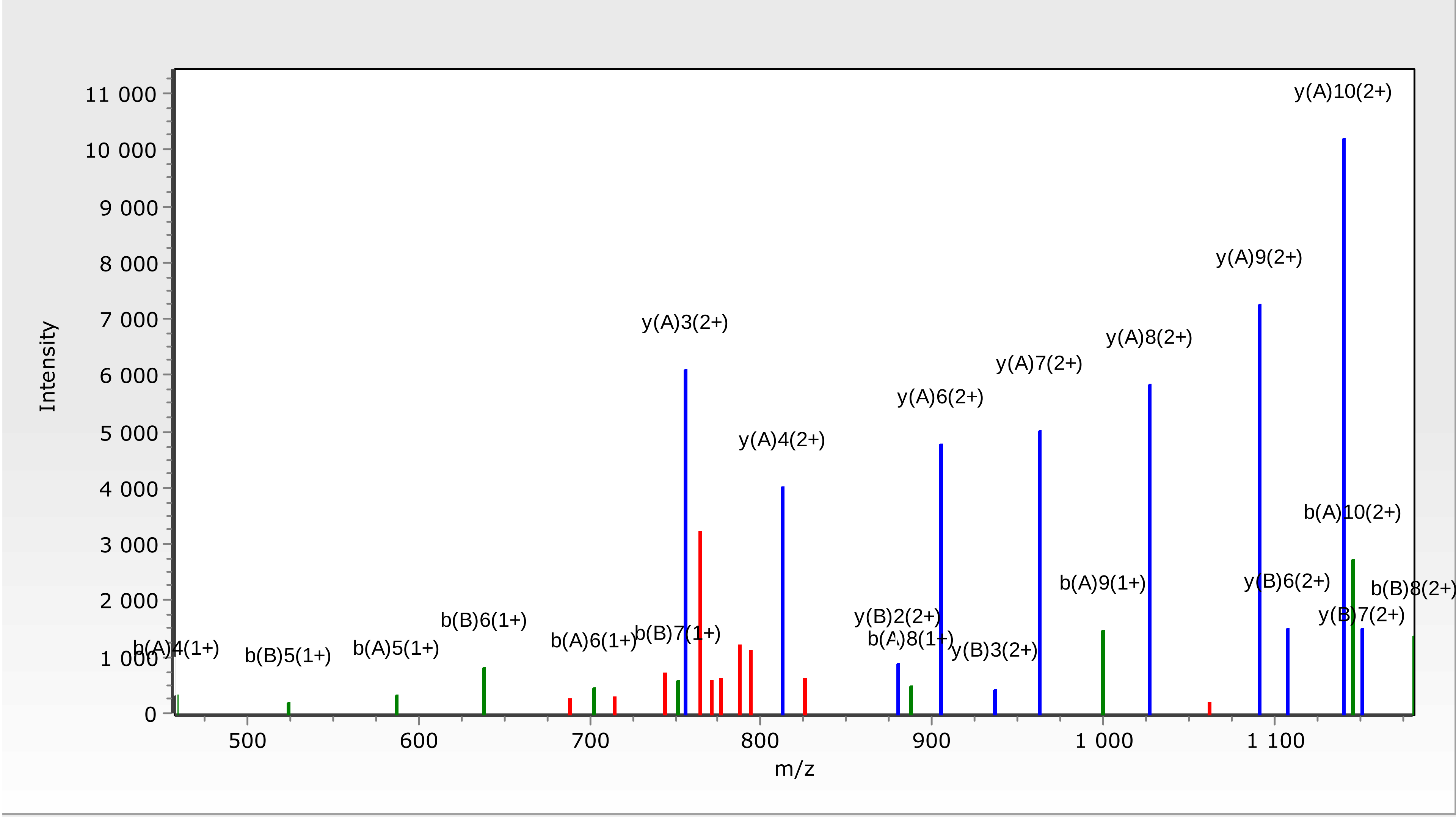

DNAJB6 K70-K67, GKEGLNGGGGGGSHF + DIYDKY

Fig. S6 MSMS-spectra for Table 2

Dataset #1
Duplicate samples with crosslinked DNAJB6 oligomers

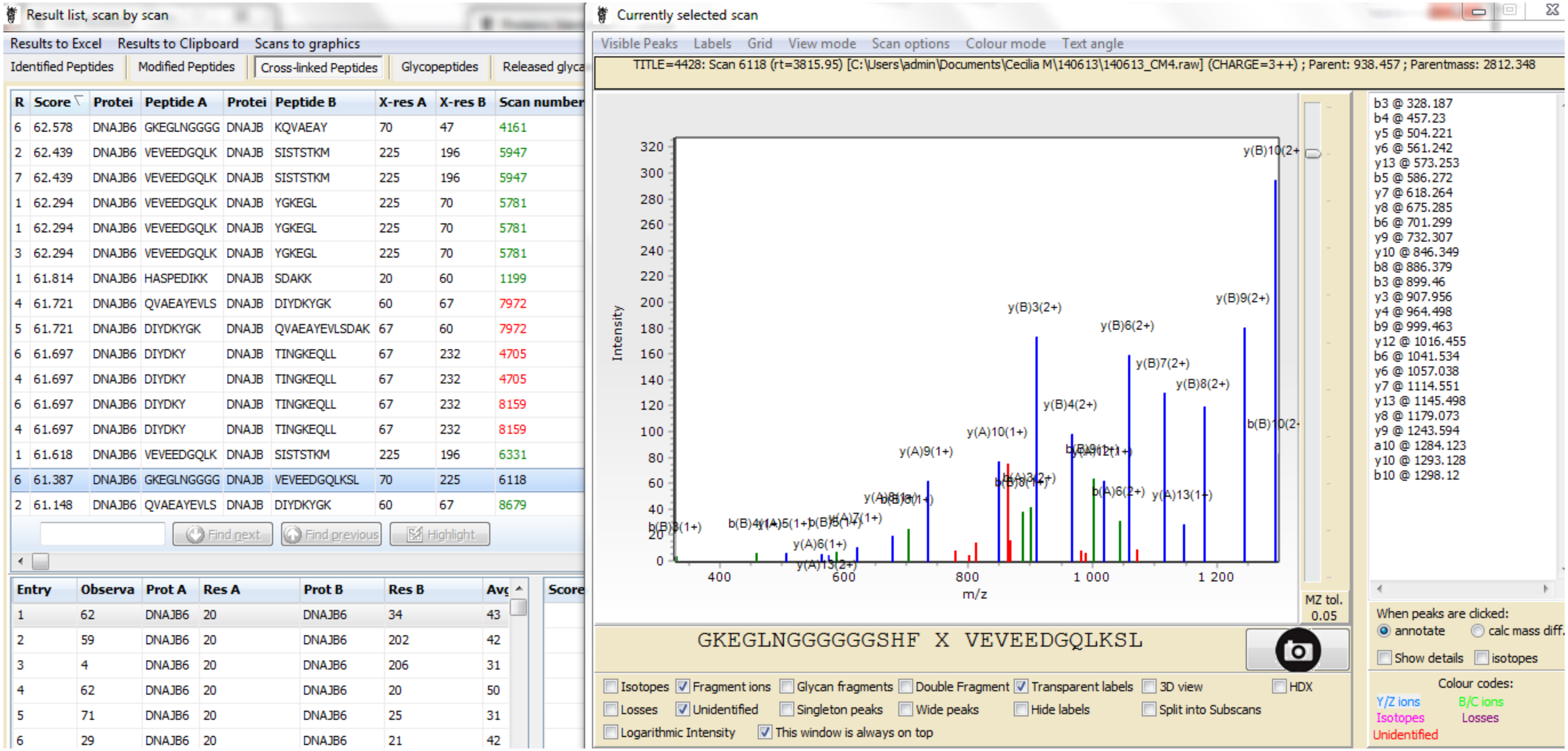

DNAJB6 K70-K225, GKEGLNGGGGGGSHF + VEVEEDGQLKSL

Fig. S6 MSMS-spectra for Table 2

Dataset #1
Duplicate samples with crosslinked DNAJB6 oligomers

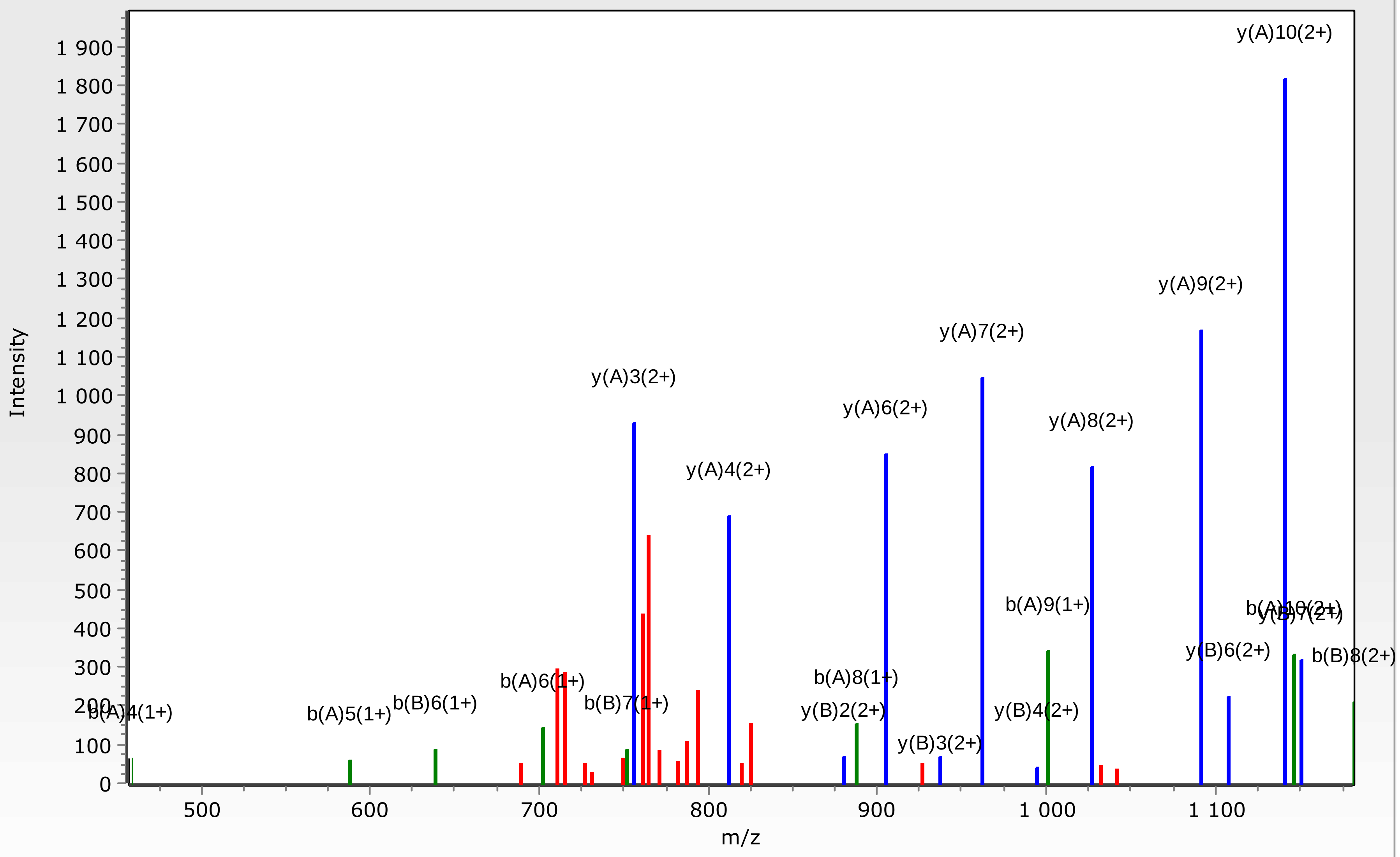

DNAJB6 K225-K20, VEVEEDGQLKSL+ HASPEDIKK

Fig. S6 MSMS-spectra for Table 2

Dataset #1
Duplicate samples with crosslinked DNAJB6 oligomers

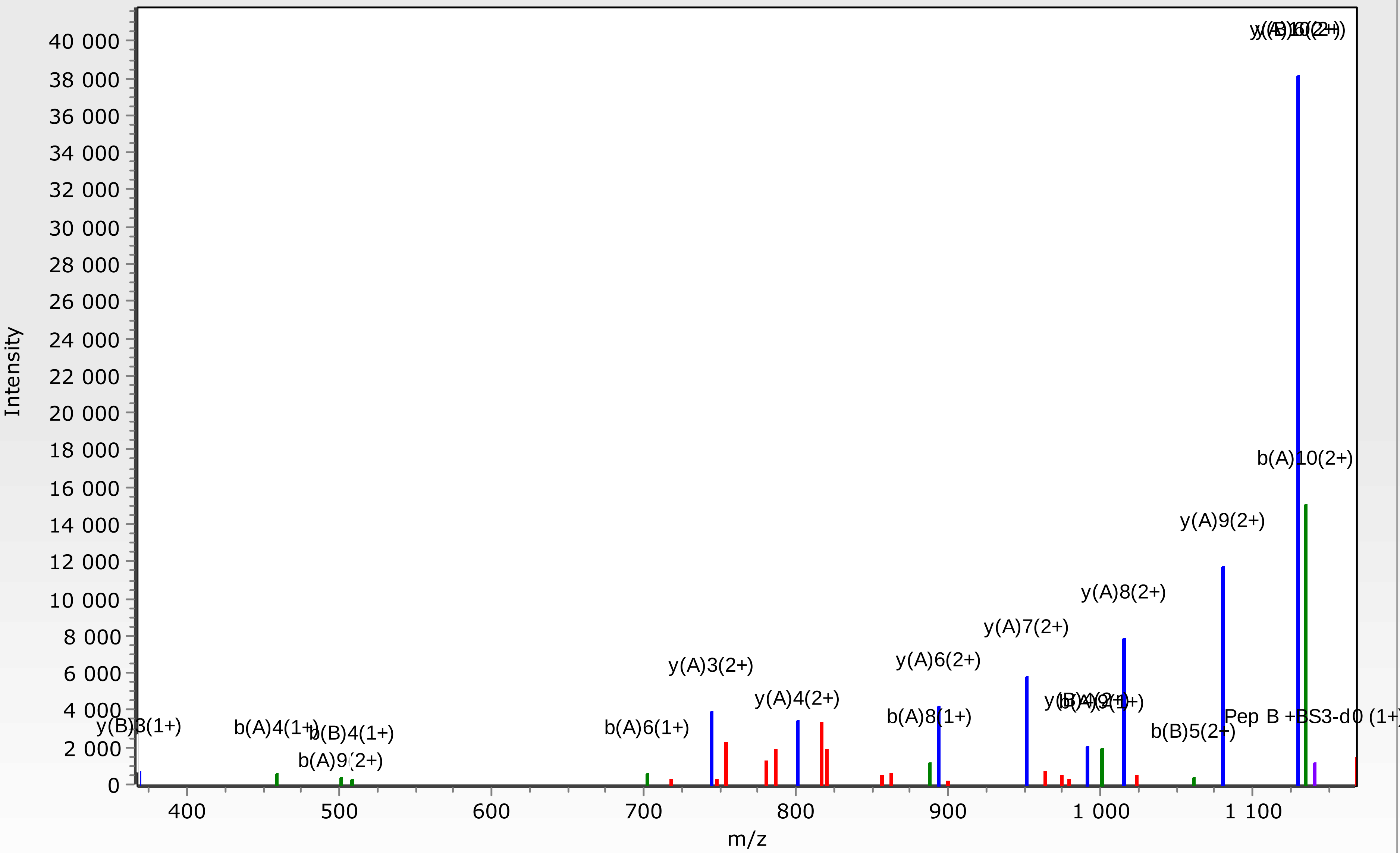

DNAJB6 K225-K67, VEVEEDGQLKSL + DIYDGYGK

Fig. S6 MSMS-spectra for Table 2

Dataset #1
Duplicate samples with crosslinked DNAJB6 oligomers

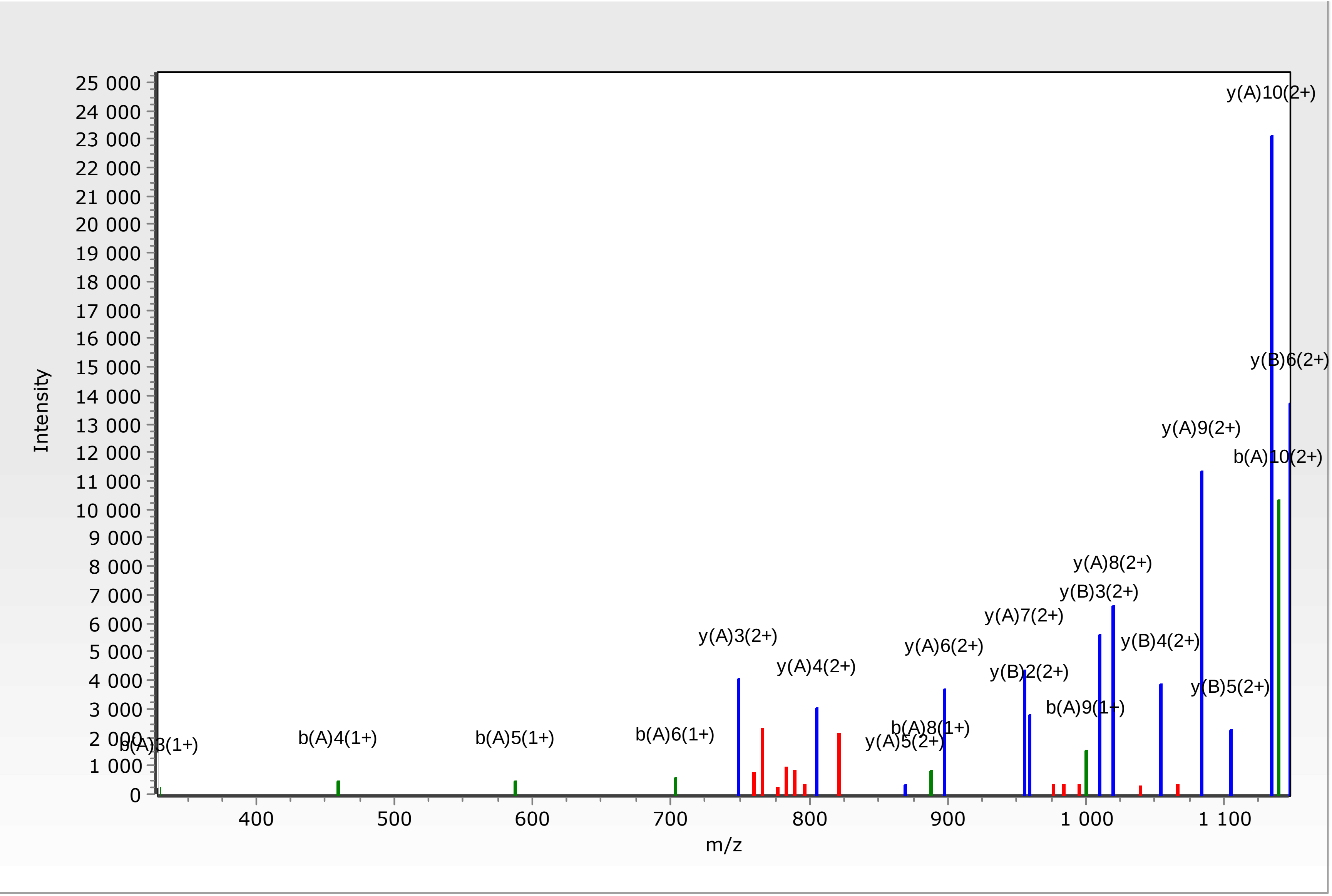

DNAJB6 K225-K196, VEVEEDGQLKSL + SISTSTKM

Fig. S6 MSMS-spectra for Table 2

Dataset #1
Duplicate samples with crosslinked DNAJB6 oligomers

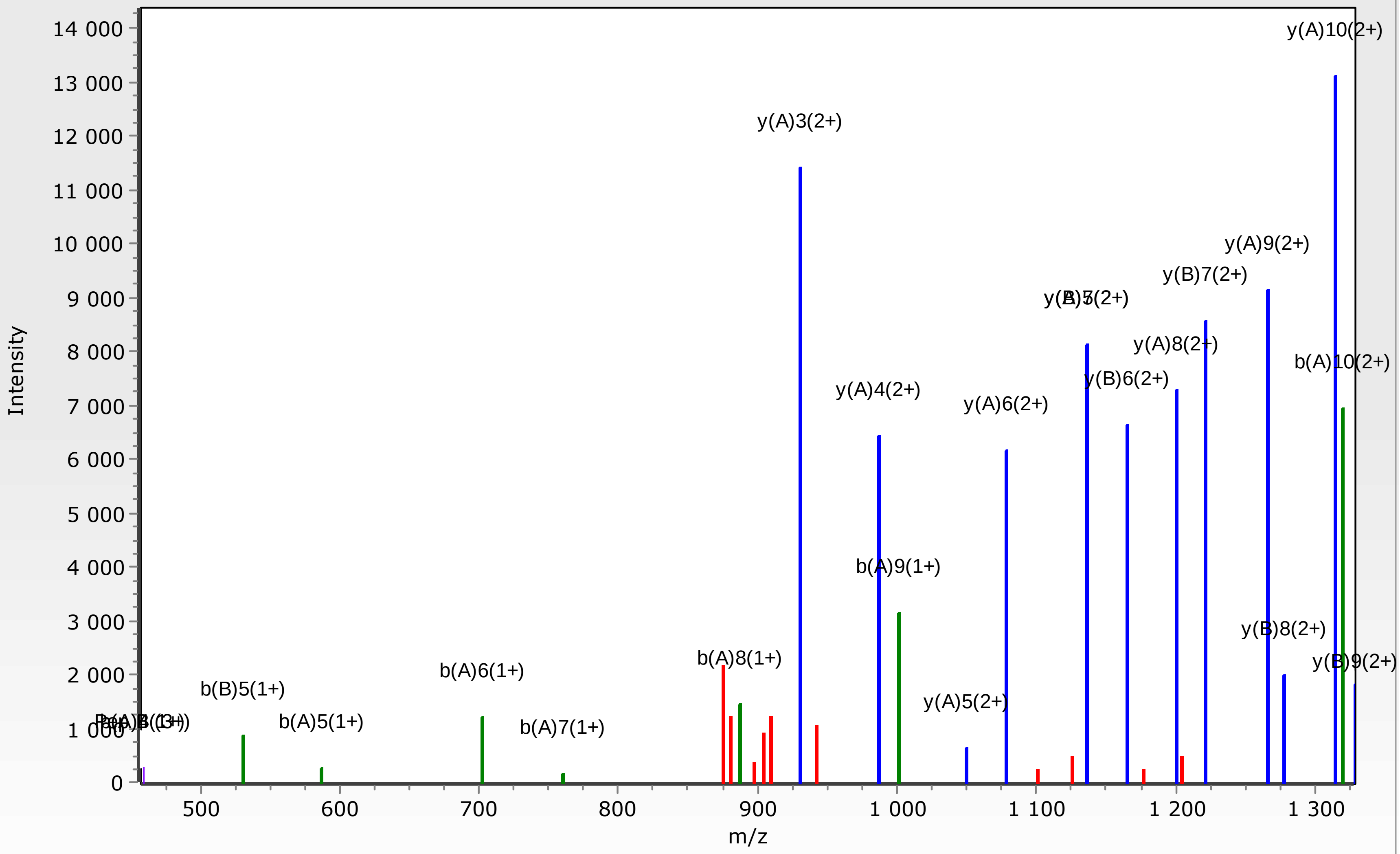

DNAJB6 K225-K232, VEVEEDGQLKSL + SLTINGKEQLL

Fig. S6 MSMS-spectra for Table 2

Dataset #1
Duplicate samples with crosslinked DNAJB6 oligomers

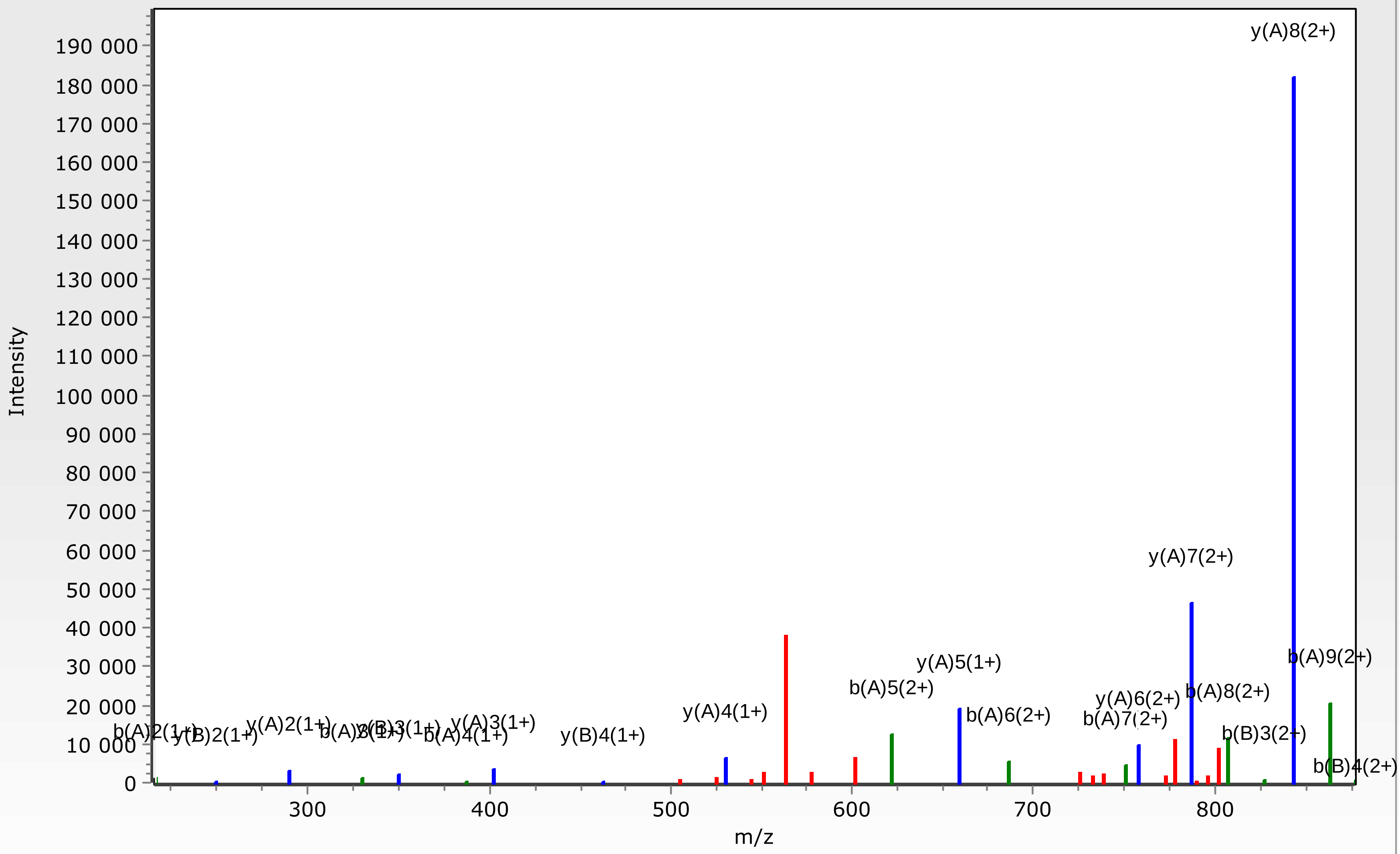

DNAJB6 K225-K202, TINGKEQLL + KITTK

Fig. S6 MSMS-spectra for Table 2

Dataset #1
Duplicate samples with crosslinked DNAJB6 oligomers

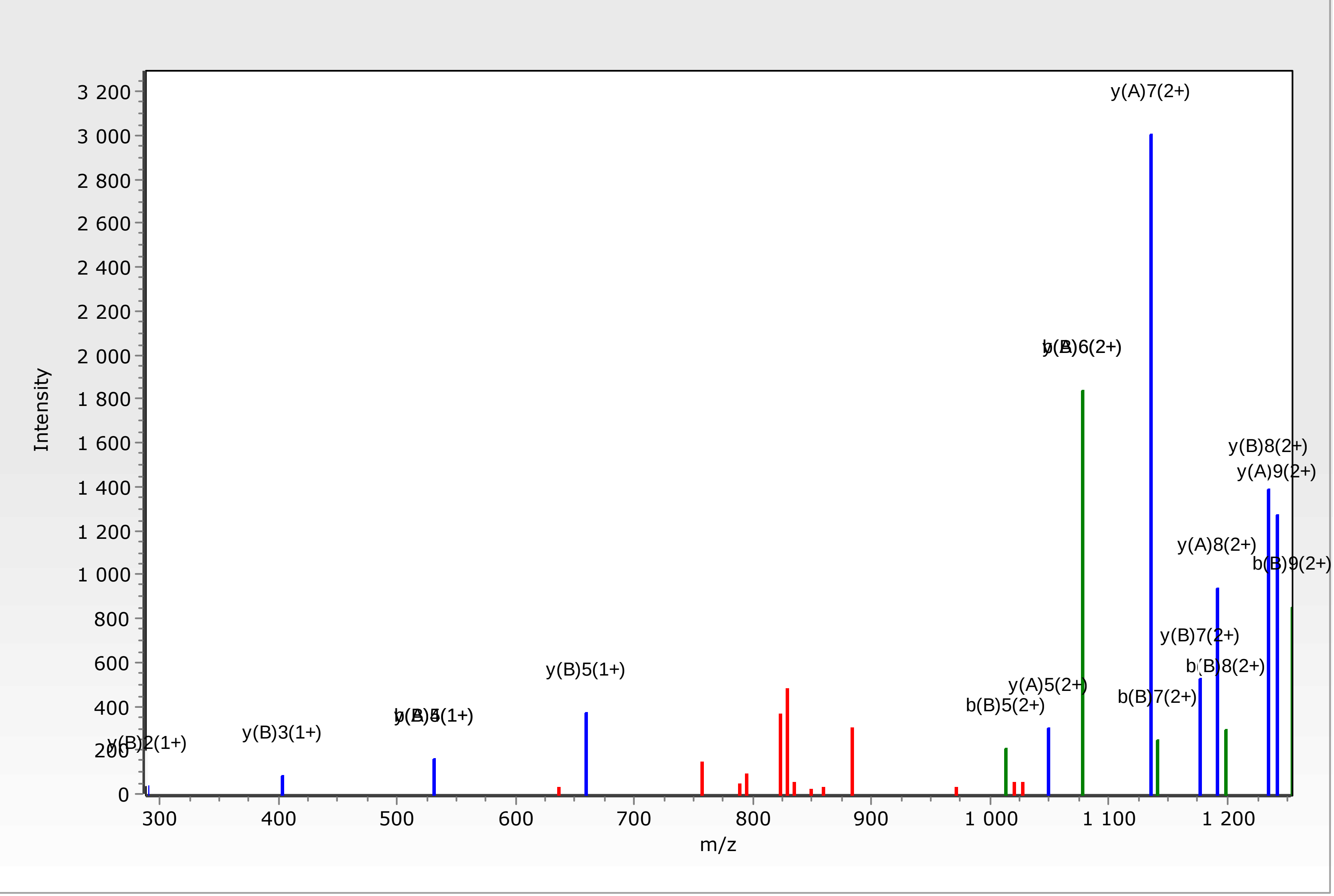

DNAJB6 K232-K232, SLTINGKEQLL + TINGKEQLLR

Fig. S6 MSMS-spectra for Table 2

Dataset #1

Duplicate samples with crosslinked DNAJB6 oligomers

K232xK232, six scans with charge 3+ (left) and charge 4+ (right)

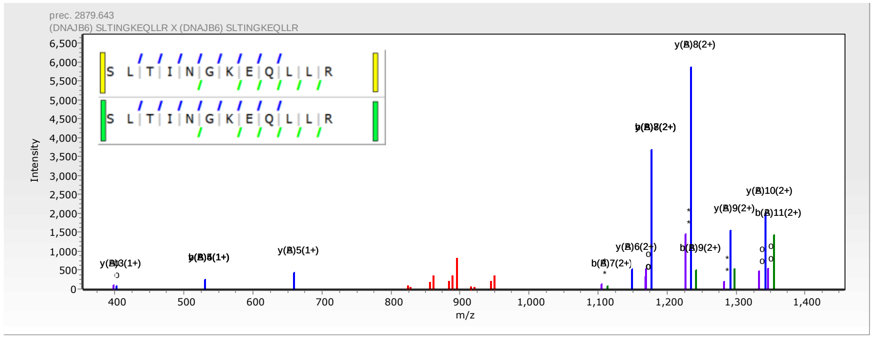

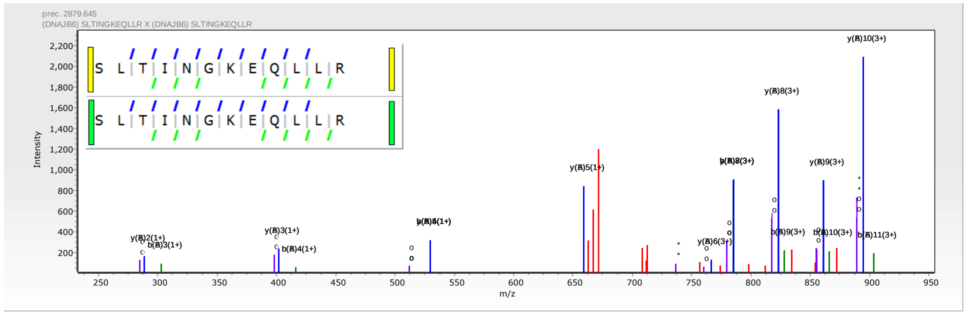

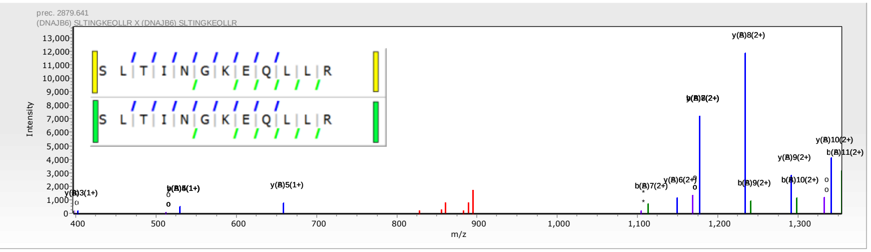

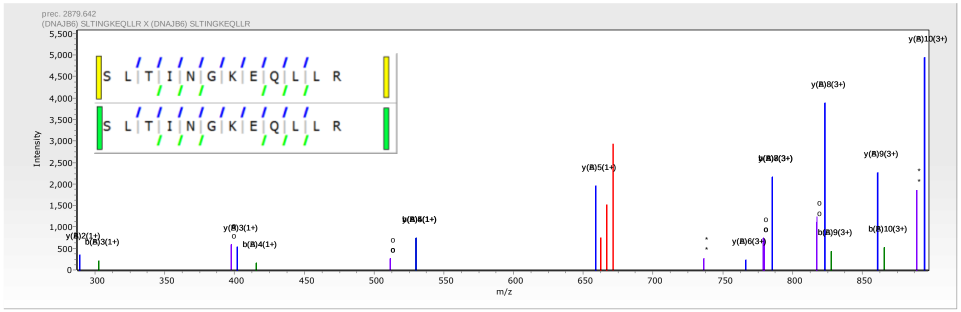

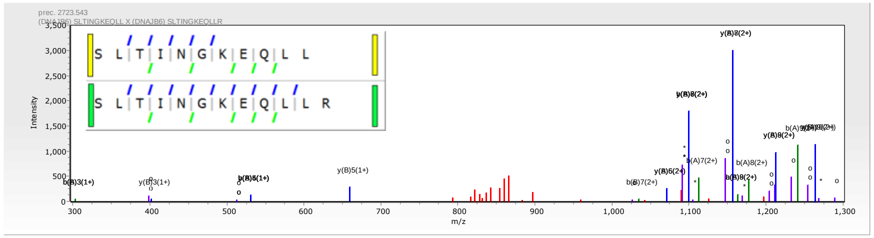

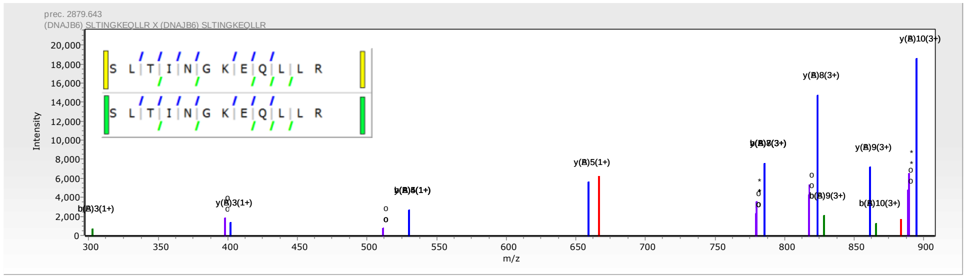

Fig. S6 MSMS-spectra for Table 2

Dataset #1
Duplicate samples with crosslinked DNAJB6 oligomers

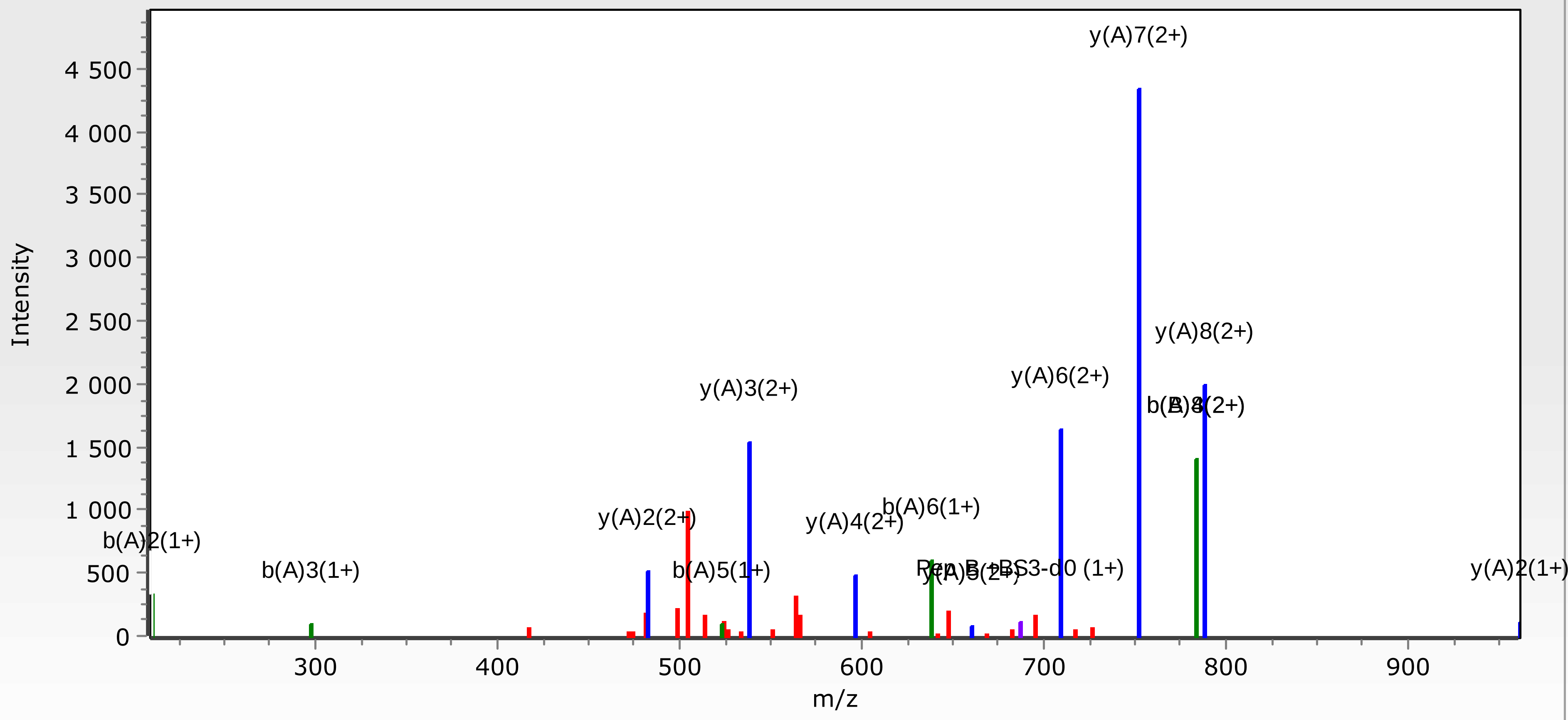

DNAJB6 K20-K60, HASPEDIKK + SDAKK

Fig. S6 MSMS-spectra for Table 2

Dataset #2
Duplicate samples with crosslinked 1:1 mixture of DNAJB6 oligomers and Aβ peptide

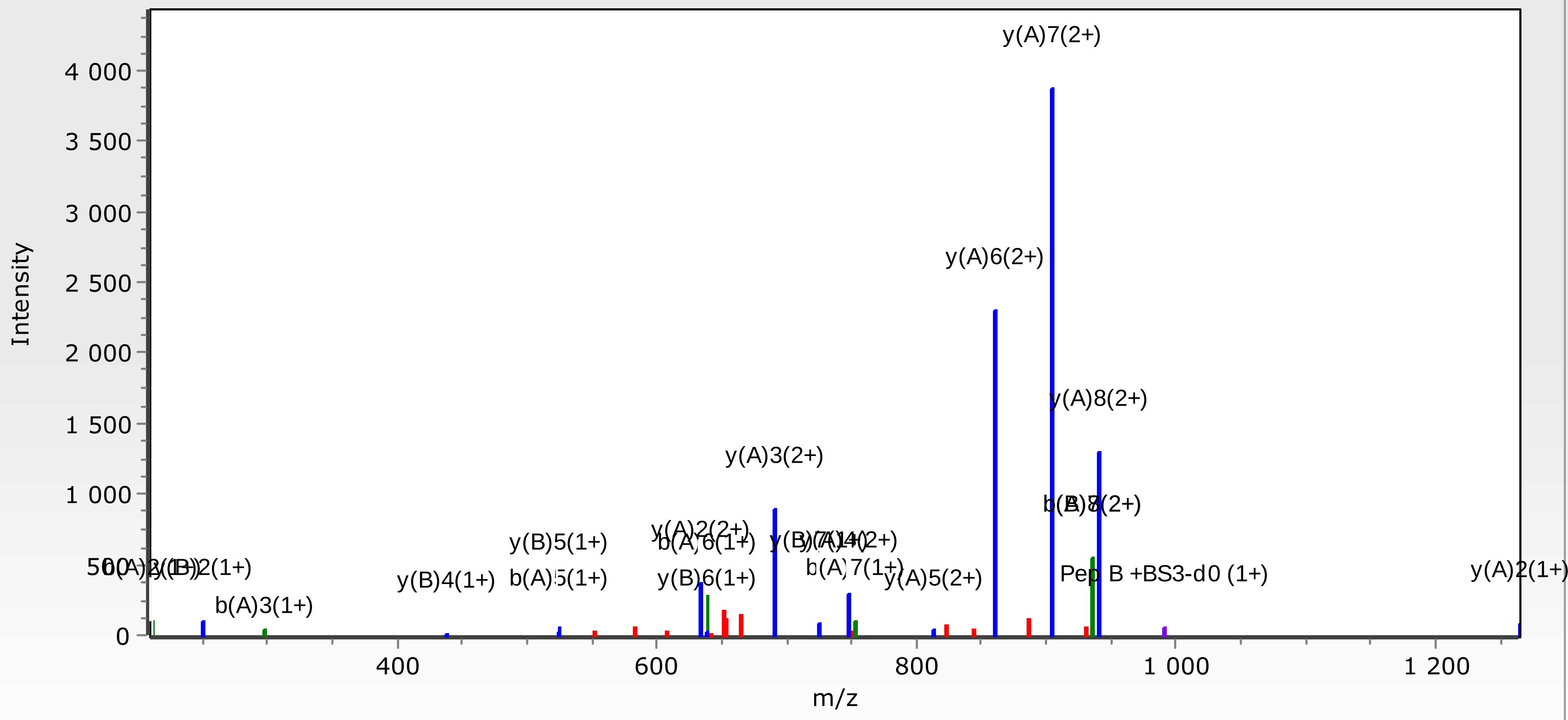

DNAJB6 K20-K189, HASPEDIKK + KSISTSTK

Fig. S6 MSMS-spectra for Table 2

Dataset #2
Duplicate samples with crosslinked 1:1 mixture of DNAJB6 oligomers and Aβ peptide

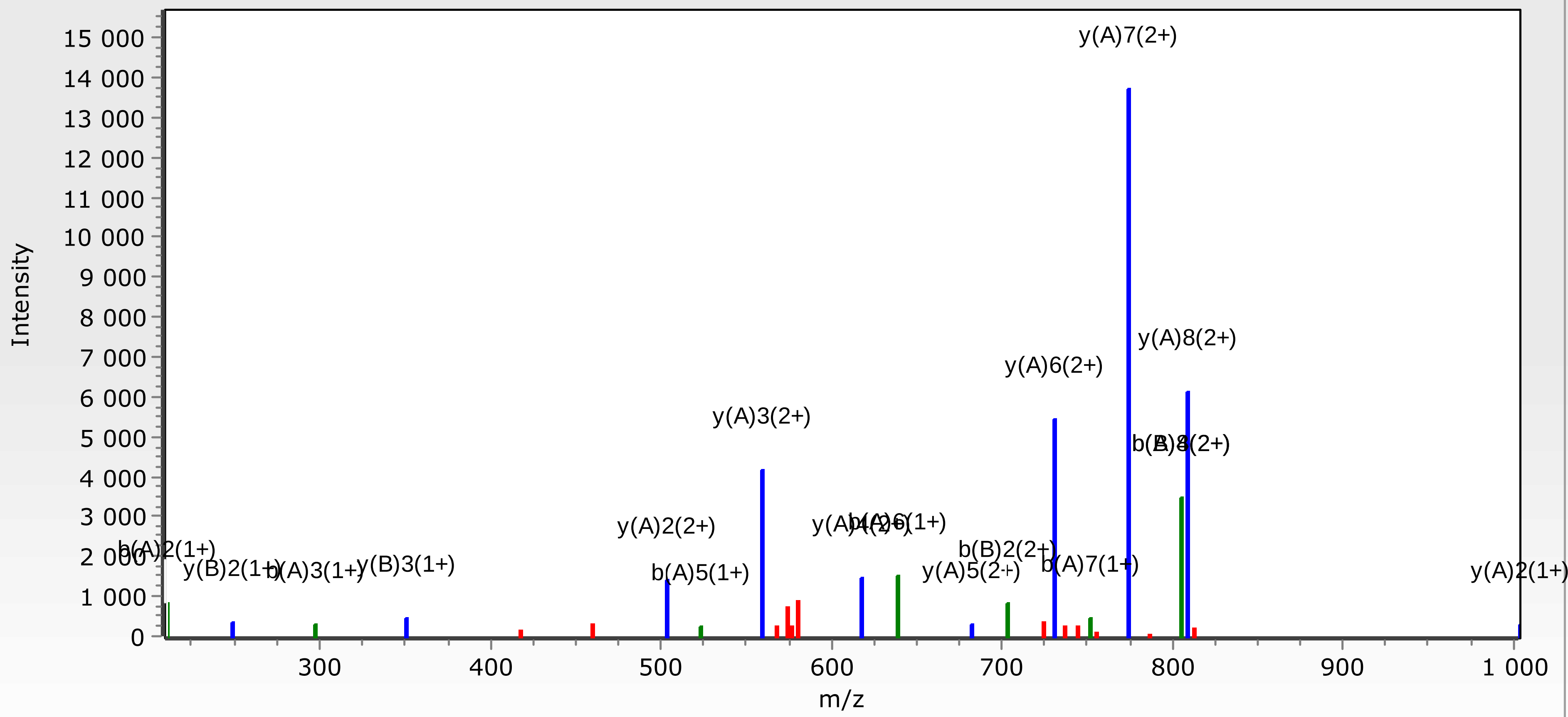

DNAJB6 K20-K202, HASPEDIKK + KITTK

Fig. S6 MSMS-spectra for Table 2

Dataset #2
Duplicate samples with crosslinked 1:1 mixture of DNAJB6 oligomers and Aβ peptide

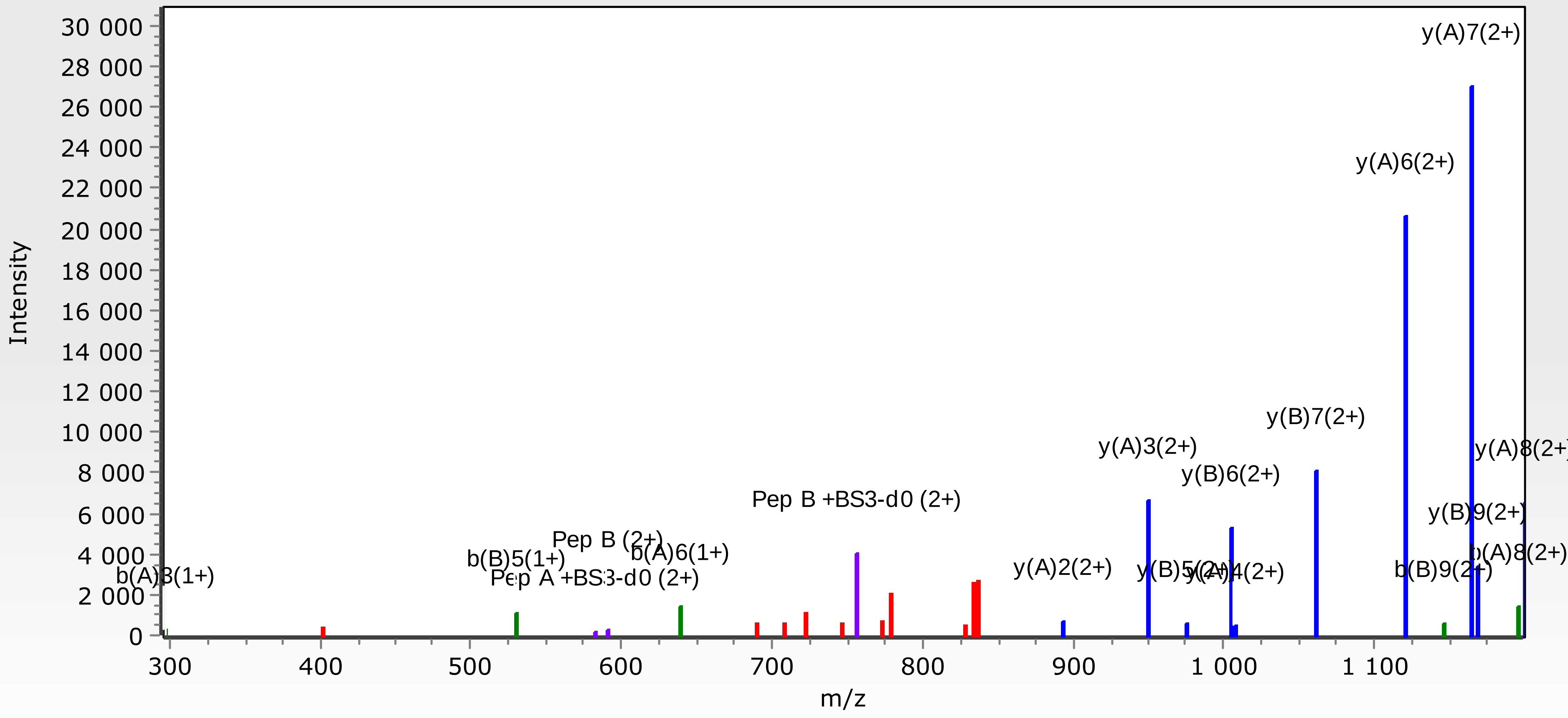

DNAJB6 K20-K232, HASPEDIKK + SLTINGKEQLL

Fig. S6 MSMS-spectra for Table 2

Dataset #2
Duplicate samples with crosslinked 1:1 mixture of DNAJB6 oligomers and Aβ peptide

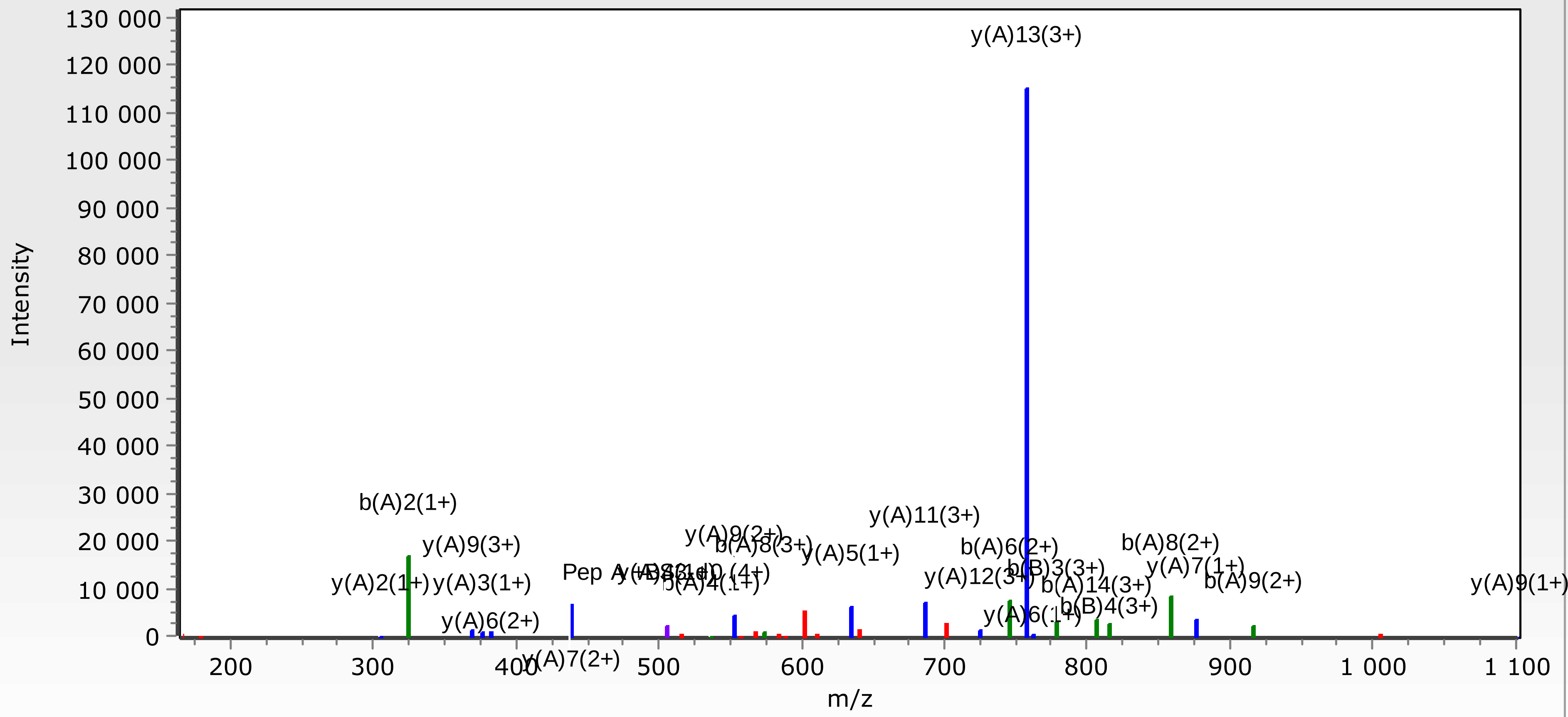

DNAJB6 K34-K25, WHPDKNPENKEEAER + KLALK

Fig. S6 MSMS-spectra for Table 2

Dataset #2
Duplicate samples with crosslinked 1:1 mixture of DNAJB6 oligomers and Aβ peptide

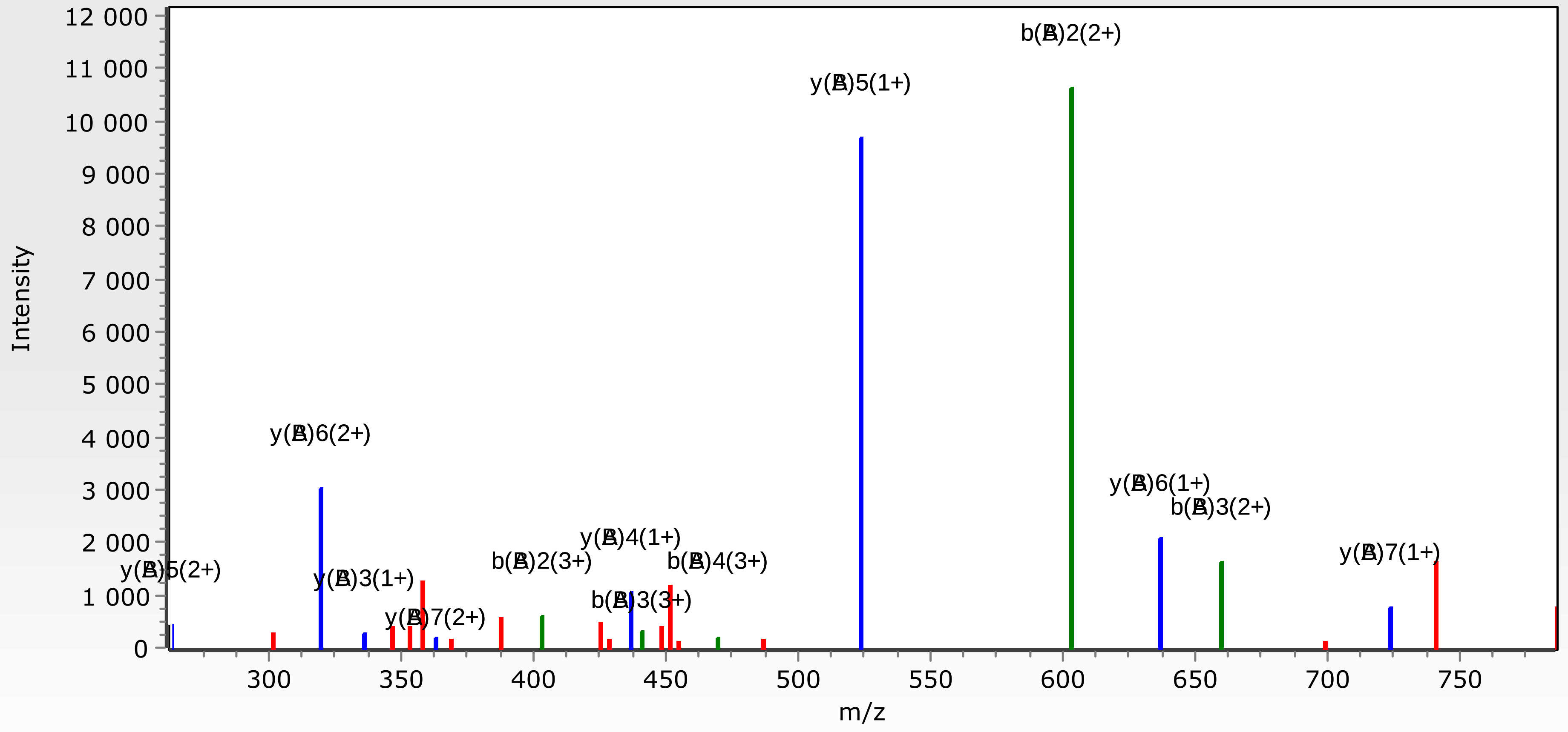

DNAJB6 K189-K189, KSISTSTK + KSISTSTK

Fig. S6 MSMS-spectra for Table 2

Dataset #2
Duplicate samples with crosslinked 1:1 mixture of DNAJB6 oligomers and Aβ peptide

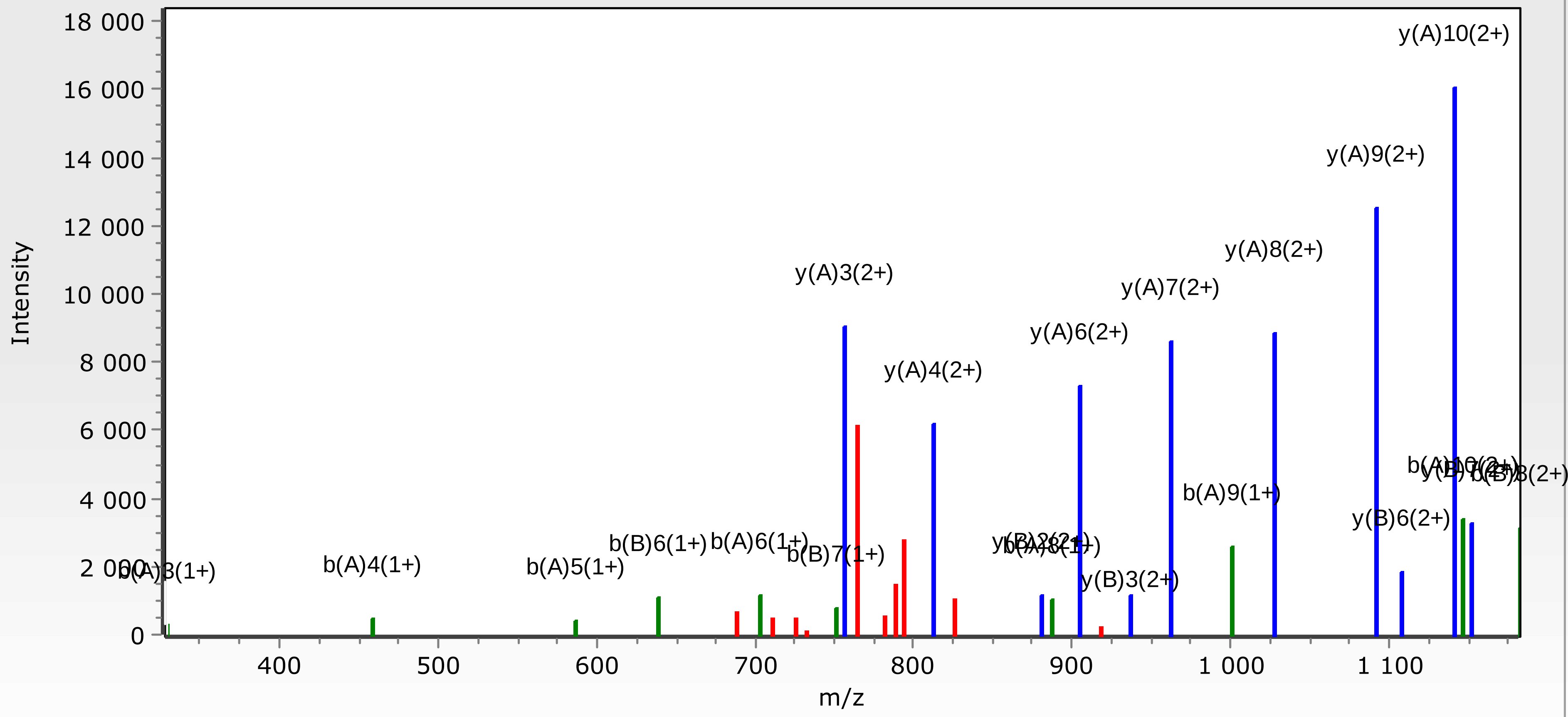

DNAJB6 K225-K20, VEVEEDGQLKSL + HASPEDIKK

Fig. S6 MSMS-spectra for Table 2

Dataset #2
Duplicate samples with crosslinked 1:1 mixture of DNAJB6 oligomers and Aβ peptide

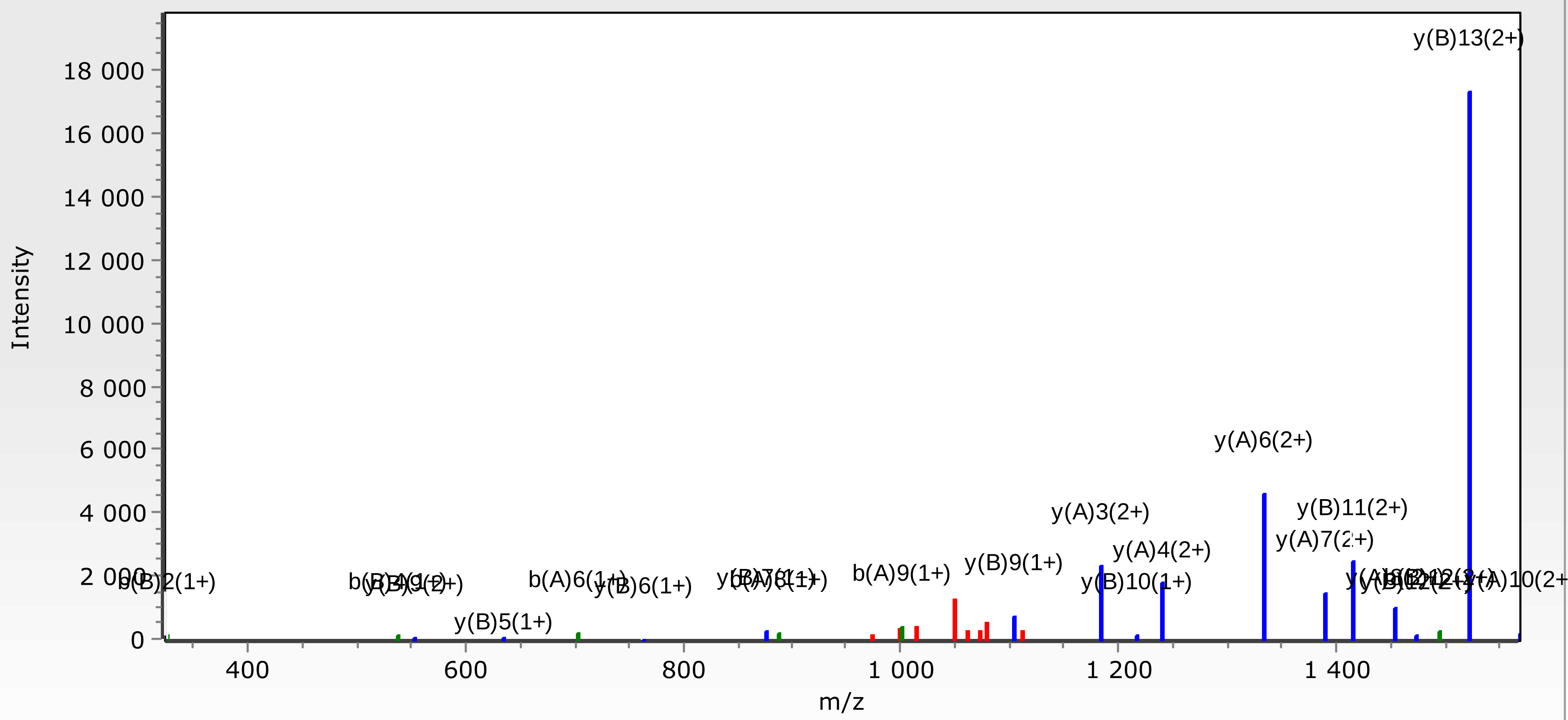

DNAJB6 K225-K34, VEVEEDGQLKSL + WHPDKNPENKEEAER

Fig. S6 MSMS-spectra for Table 2

Dataset #2
Duplicate samples with crosslinked 1:1 mixture of DNAJB6 oligomers and Aβ peptide

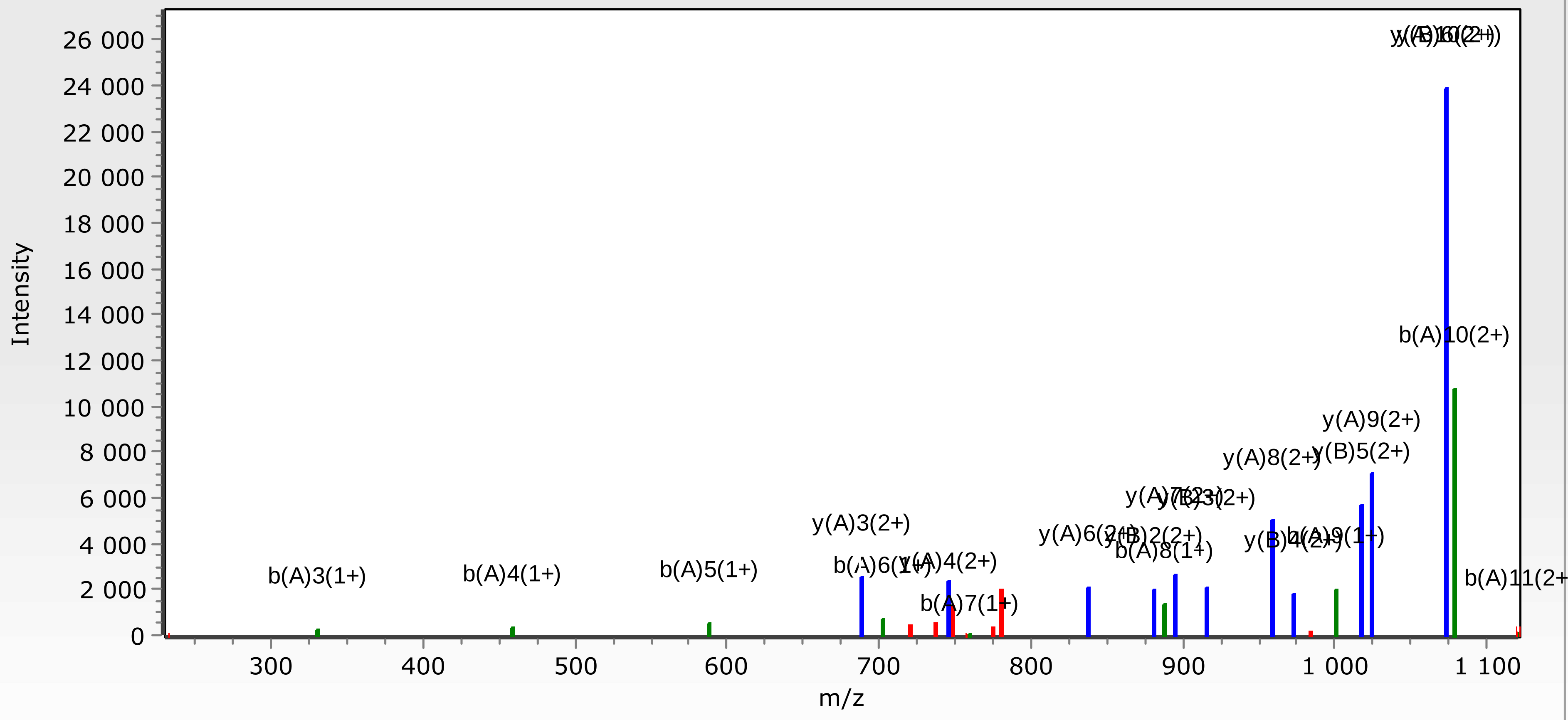

DNAJB6 K225-K60, VEVEEDGQLKSL + EVLSDAKK

Fig. S6 MSMS-spectra for Table 2

Dataset #2
Duplicate samples with crosslinked 1:1 mixture of DNAJB6 oligomers and Aβ peptide

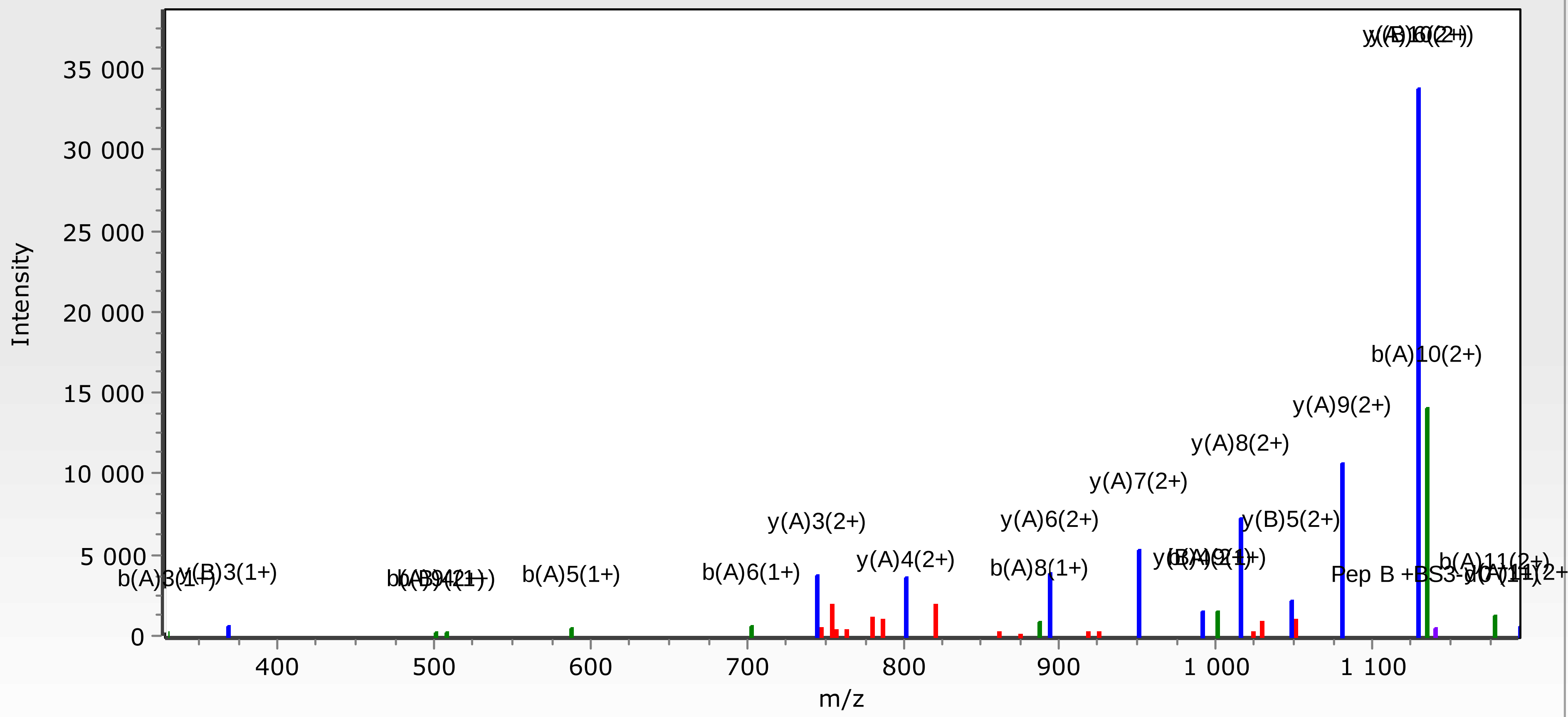

DNAJB6 K225-K67, VEVEEDGQLKSL + DIYDGYGK

Fig. S6 MSMS-spectra for Table 2

Dataset #2
Duplicate samples with crosslinked 1:1 mixture of DNAJB6 oligomers and Aβ peptide

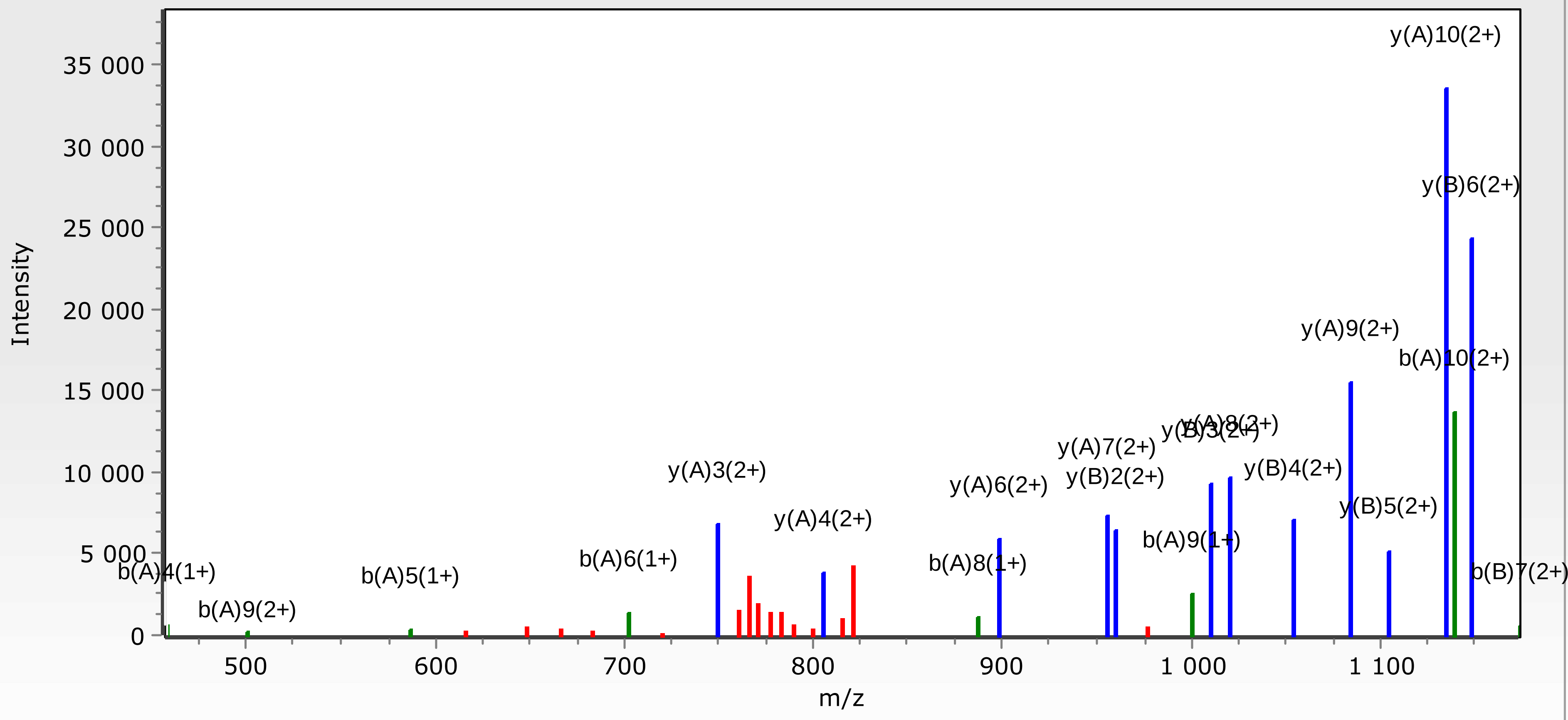

DNAJB6 K225-K196, VEVEEDGQLKSL + SISTSTKM

Fig. S6 MSMS-spectra for Table 2

Dataset #2
Duplicate samples with crosslinked 1:1 mixture of DNAJB6 oligomers and Aβ peptide

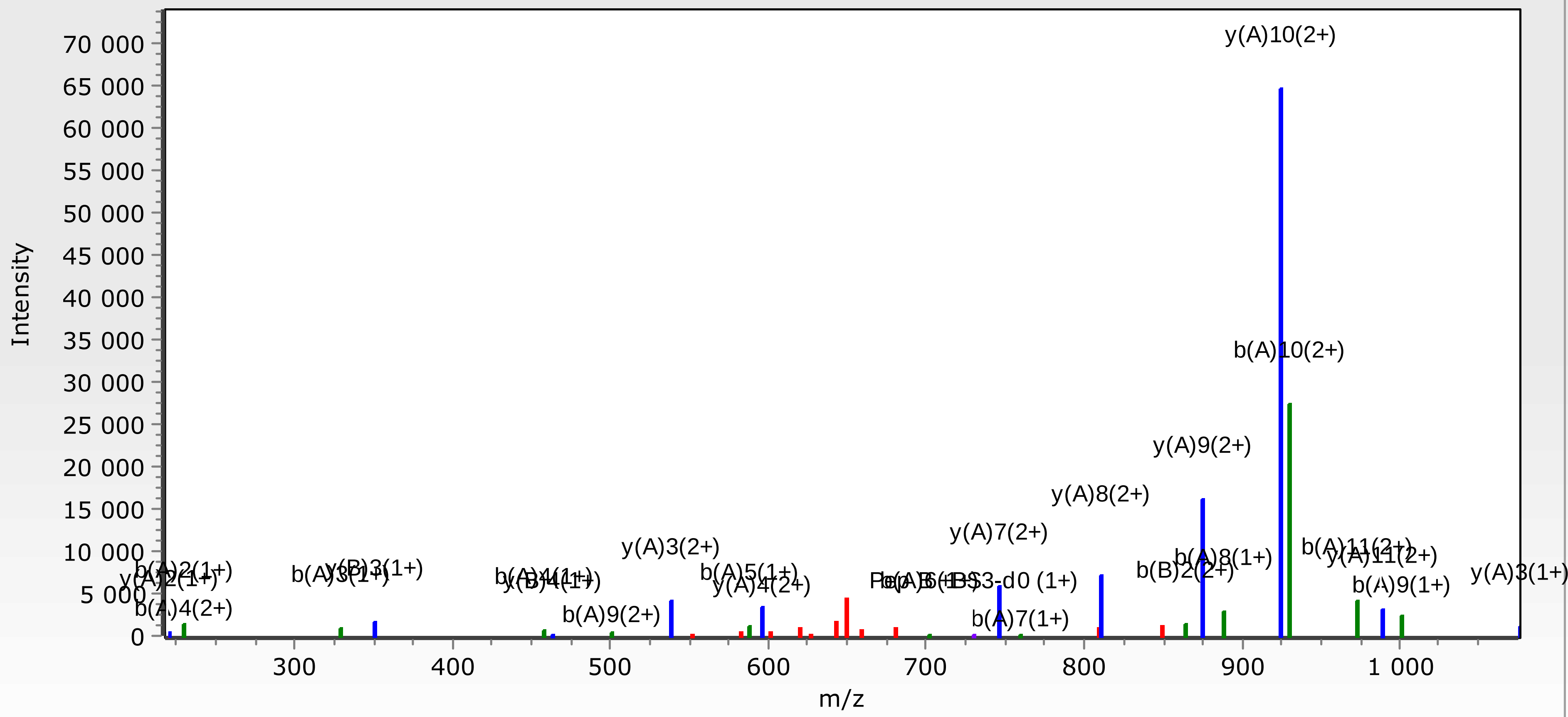

DNAJB6 K225-K202, VEVEEDGQLKSL + KITTK

Fig. S6 MSMS-spectra for Table 2

Dataset #2
Duplicate samples with crosslinked 1:1 mixture of DNAJB6 oligomers and Aβ peptide

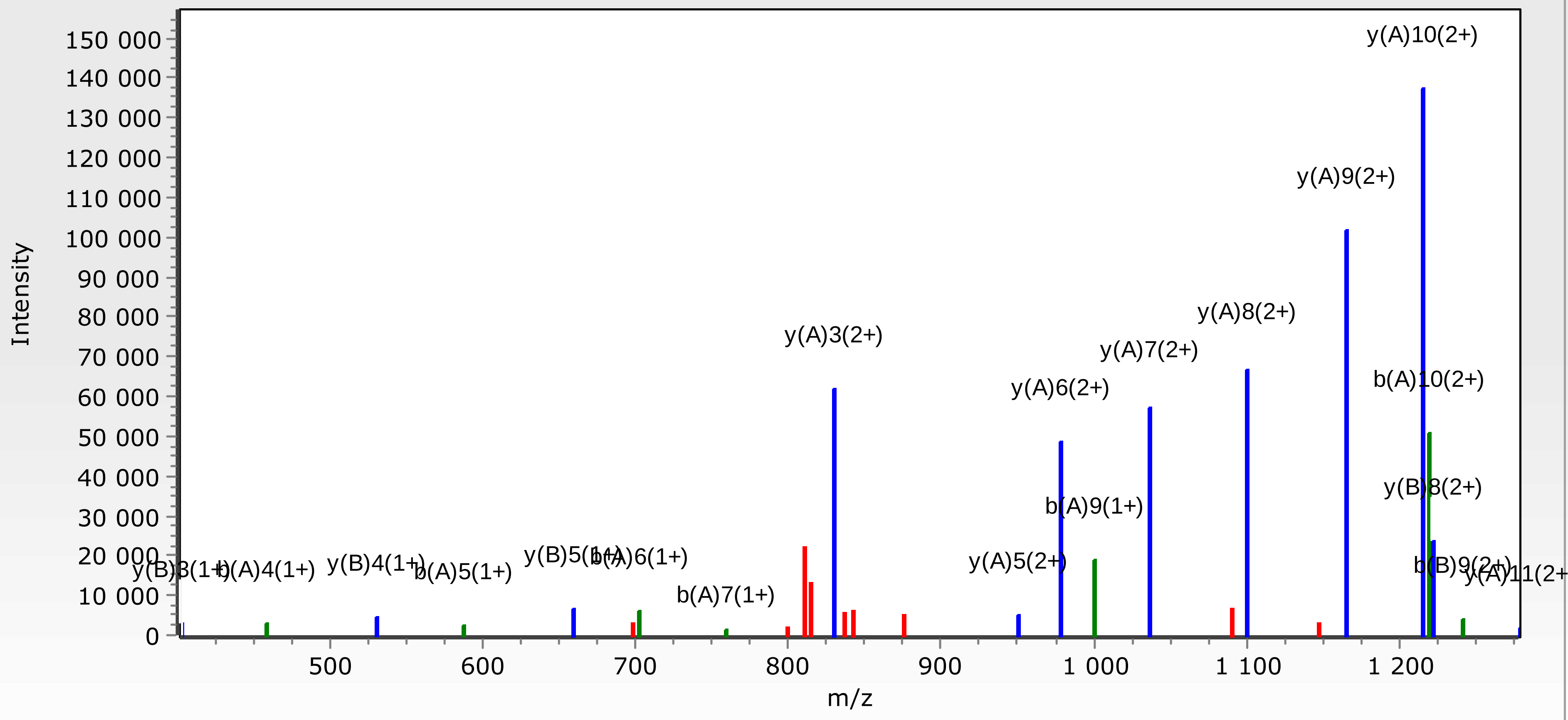

DNAJB6 K225-K232, VEVEEDGQLKSL + SLTINGKEQLL

Fig. S6 MSMS-spectra for Table 2

Dataset #2
Duplicate samples with crosslinked 1:1 mixture of DNAJB6 oligomers and Aβ peptide

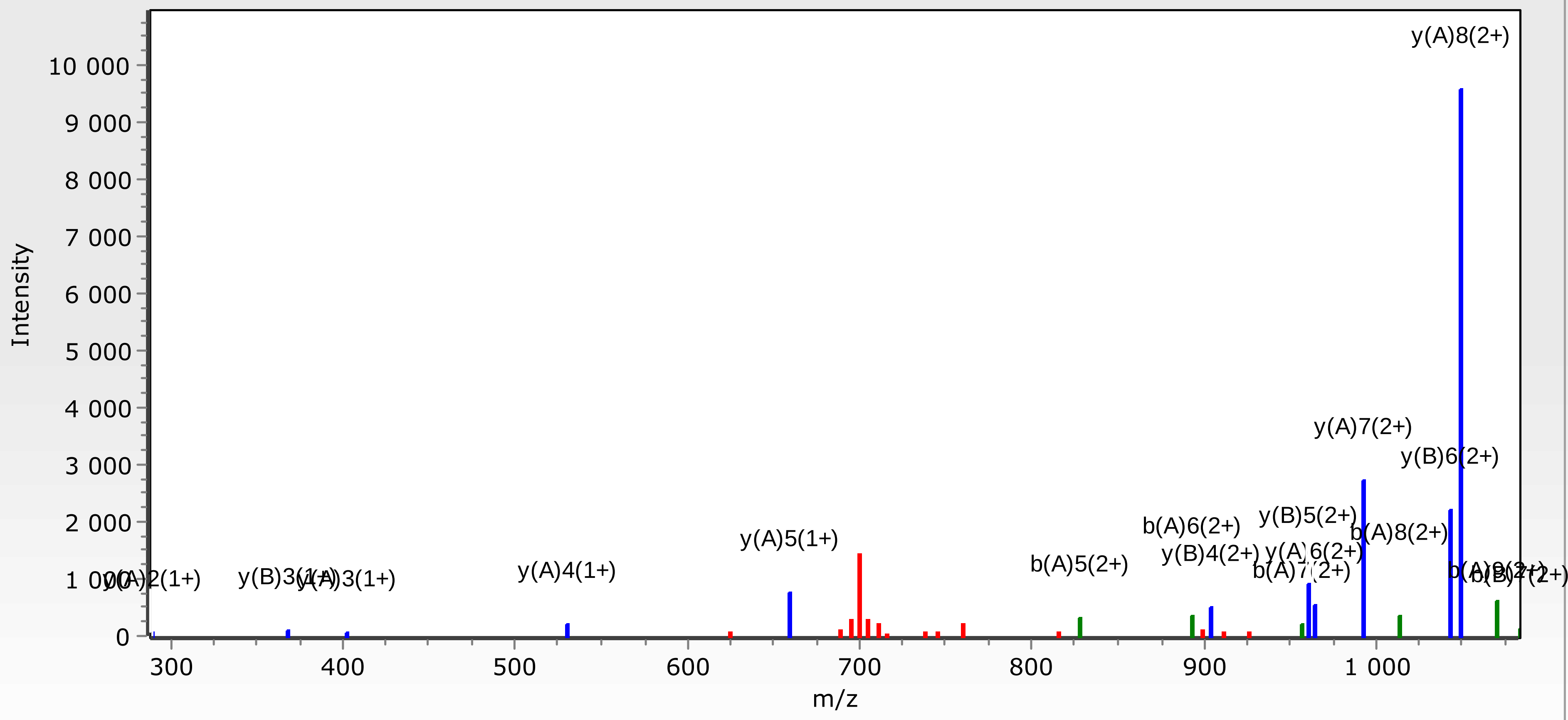

DNAJB6 K232-K67, TINGKEQLLR + DIYDKYGK

Fig. S6 MSMS-spectra for Table 2

Dataset #2
Duplicate samples with crosslinked 1:1 mixture of DNAJB6 oligomers and Aβ peptide

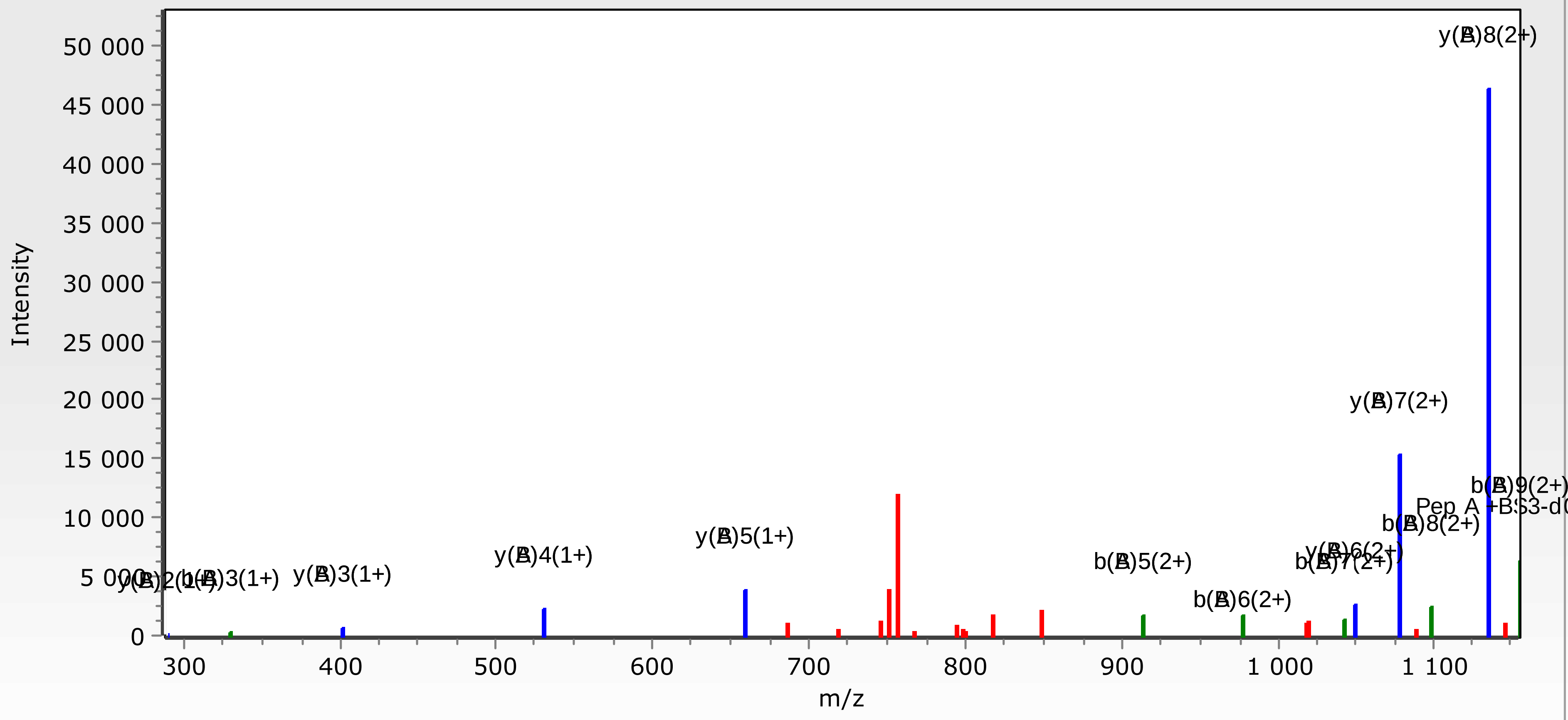

DNAJB6 K232-K232, SLTINGKEQLL + TINGKEQLLR

Fig. S6 MSMS-spectra for Table 2

Dataset #2
Duplicate samples with crosslinked 1:1 mixture of DNAJB6 oligomers and Aβ peptide

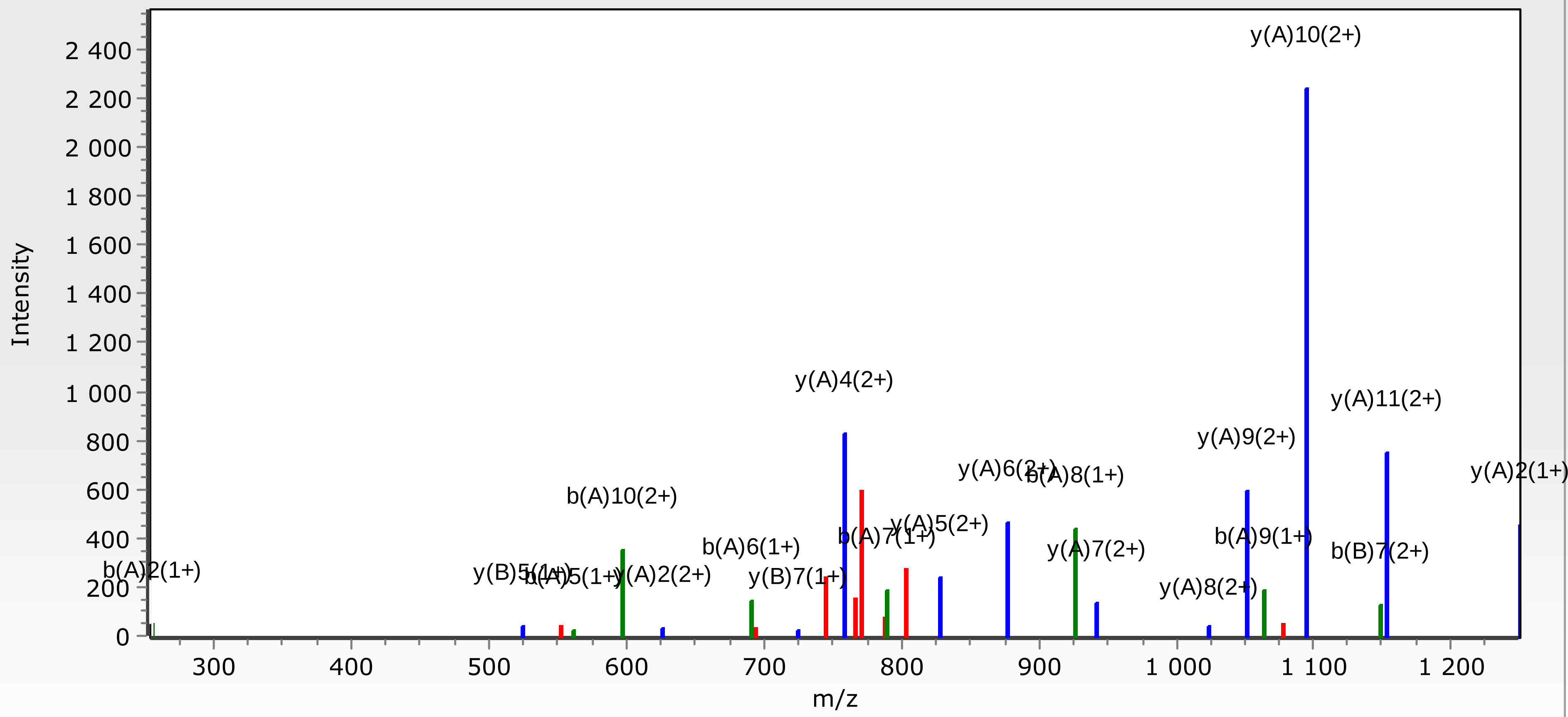

Aβ42 K16 to DNAJB6 K189, HDSGYEVHHQKL + KSISTKSTK

Fig. S6 MSMS-spectra for Table 2

Dataset #2
Duplicate samples with crosslinked 1:1 mixture of DNAJB6 oligomers and Aβ peptide

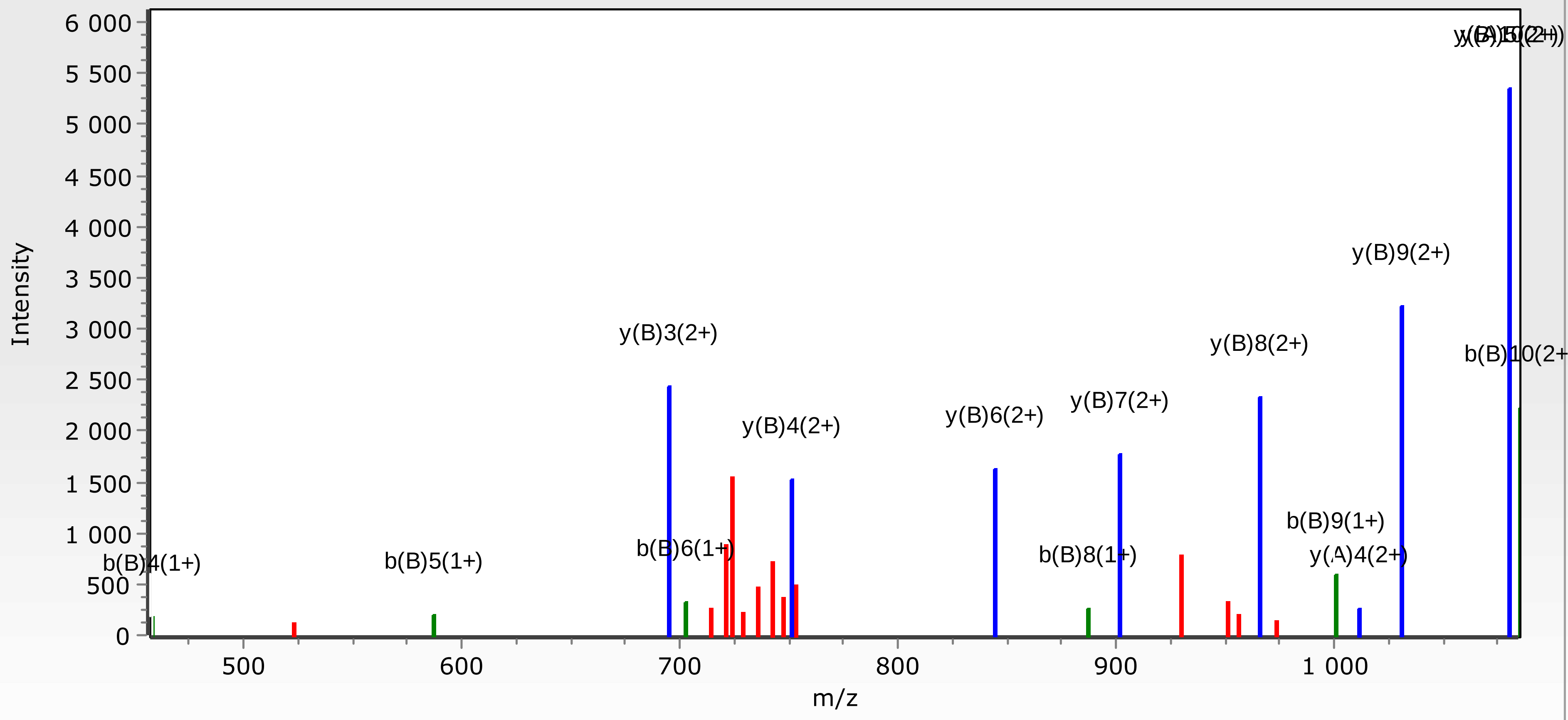

Aβ42 K16 to DNAJB6 K225, EVHHQKL + VEVEEDGQLKSL

Fig. S6 MSMS-spectra for Table 2

Dataset #2
Duplicate samples with crosslinked 1:1 mixture of DNAJB6 oligomers and Aβ peptide

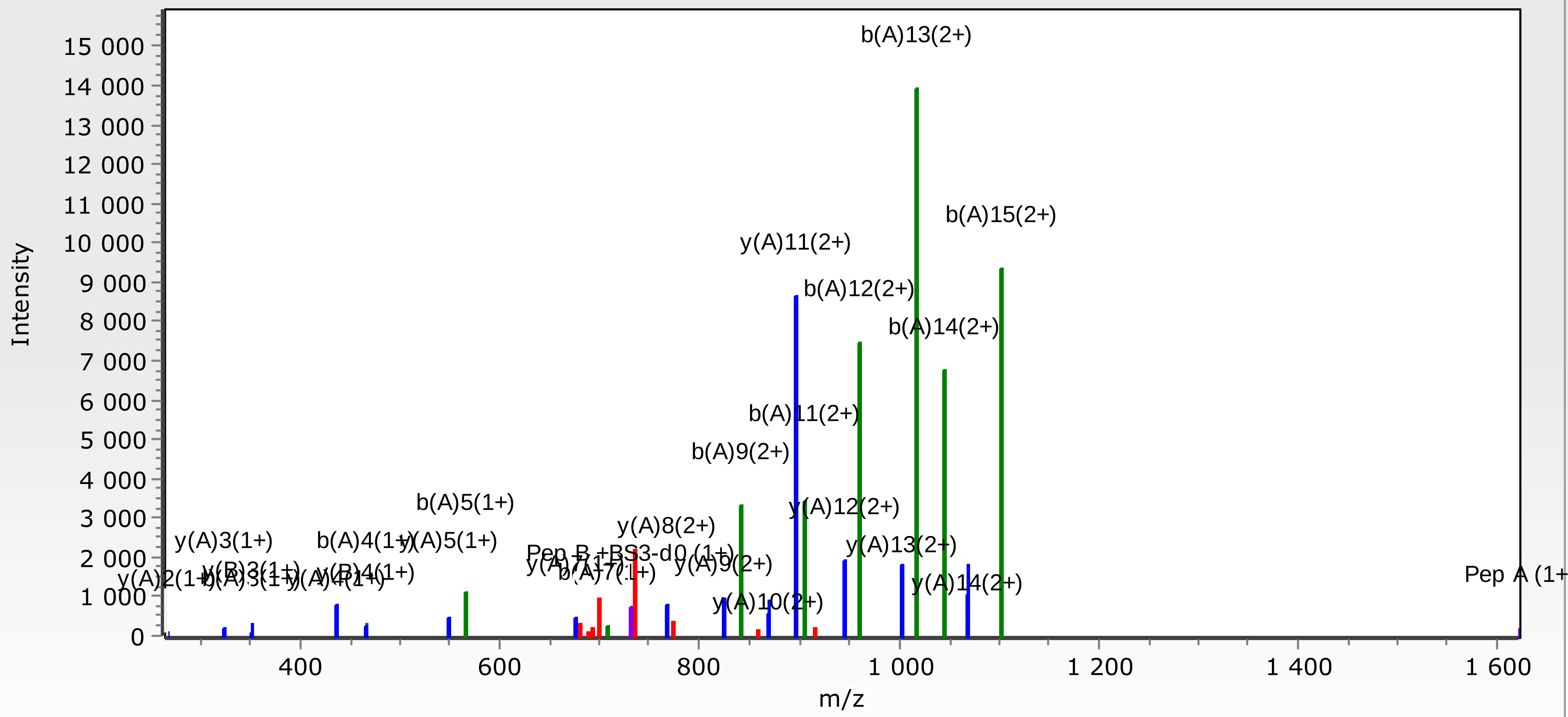

Aβ42 K28 to DNAJB6 K202, FAEDVGSNKGAIIGLM + KITTK

Fig. S6 MSMS-spectra for Table 2

Dataset #2
Duplicate samples with crosslinked 1:1 mixture of DNAJB6 oligomers and Aβ peptide

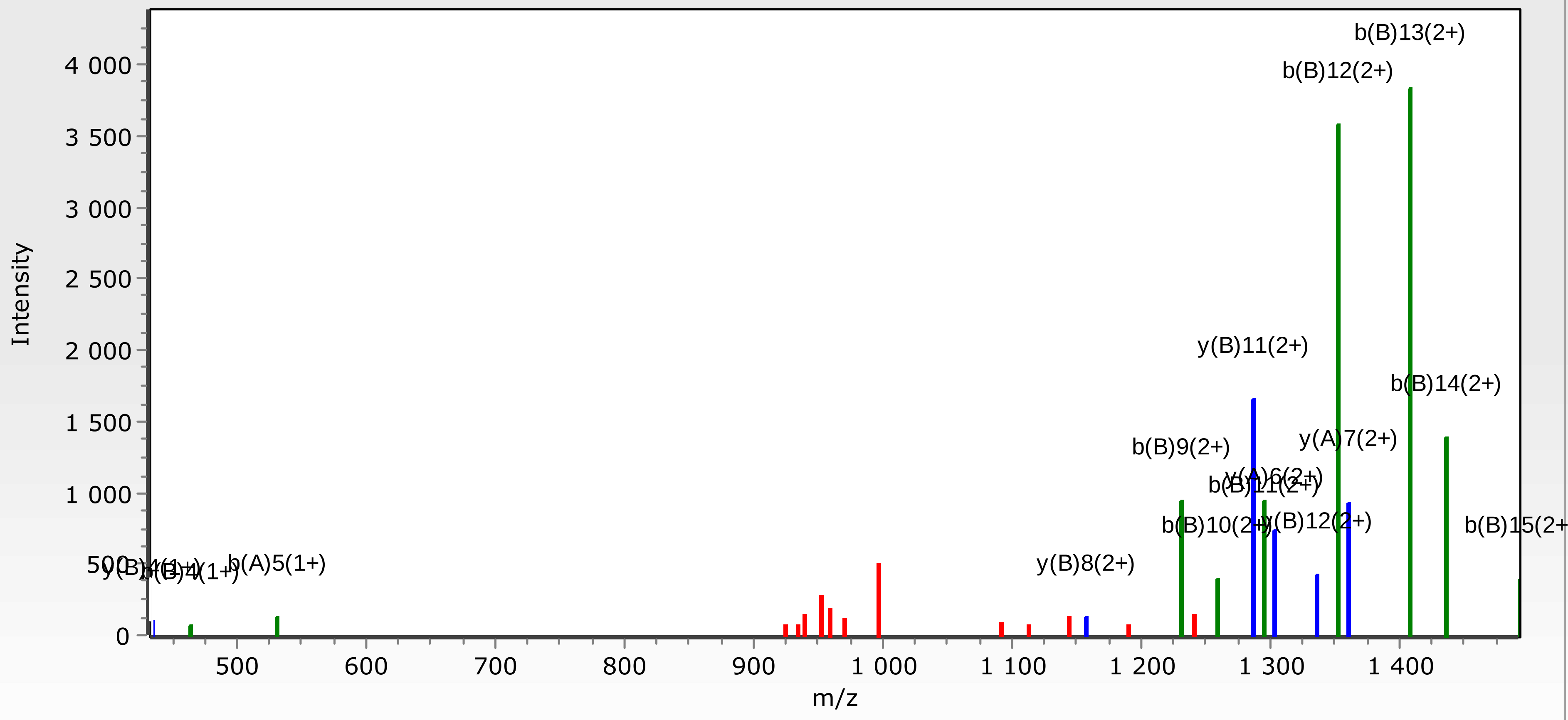

Aβ42 K28 to DNAJB6 K232, FAEDVGSNKGAIIGLM + SLTINGKEQLL

Fig. S6 MSMS-spectra for Table 2

Dataset #2
Duplicate samples with crosslinked 1:1 mixture of DNAJB6 oligomers and Aβ peptide

Fig. S7 MSMS-spectra for crosslinks for 1:1 mix of DNAJB6 that is unlabeled (^{14}N) or labelled (^{15}N). Related to Table 2.

Color coding in MSMS spectra:
y-ions (blue), b-ions (green), peptide A/B ions (purple), unexplained (red).

Peptide with longest sequence is referred to as A peptide.

170625 Searches by CE on DNAJB6 for subunit exchange, mgf-files used:

Samples are insoldig (run in Lund 160627 by KB, processed by CE with Sept-version of crosslinked Cottrell .opt-file)

MassAI-search made with MassAI version Feb-17, and these settings:
mc 2, T and CT, tolerance 10 ppm MS, 0.1 Da for crosslinked peptides, tolerance 0.2 m/z in MSMS
No fixed modification: ~~IAA~~ Variable modification: BS4 as deadend and internal crosslink, 3 modifications, BS4 as crosslinks, Also Xlink modified peptides
XODYS
The mgf-files were passed through the 125-p filter

mgf-files: DNAJB6_5_1 and DNAJB6_5_2
in DNAJB6 sequence (A and B datasets = 14N and 15N)

K189-K189

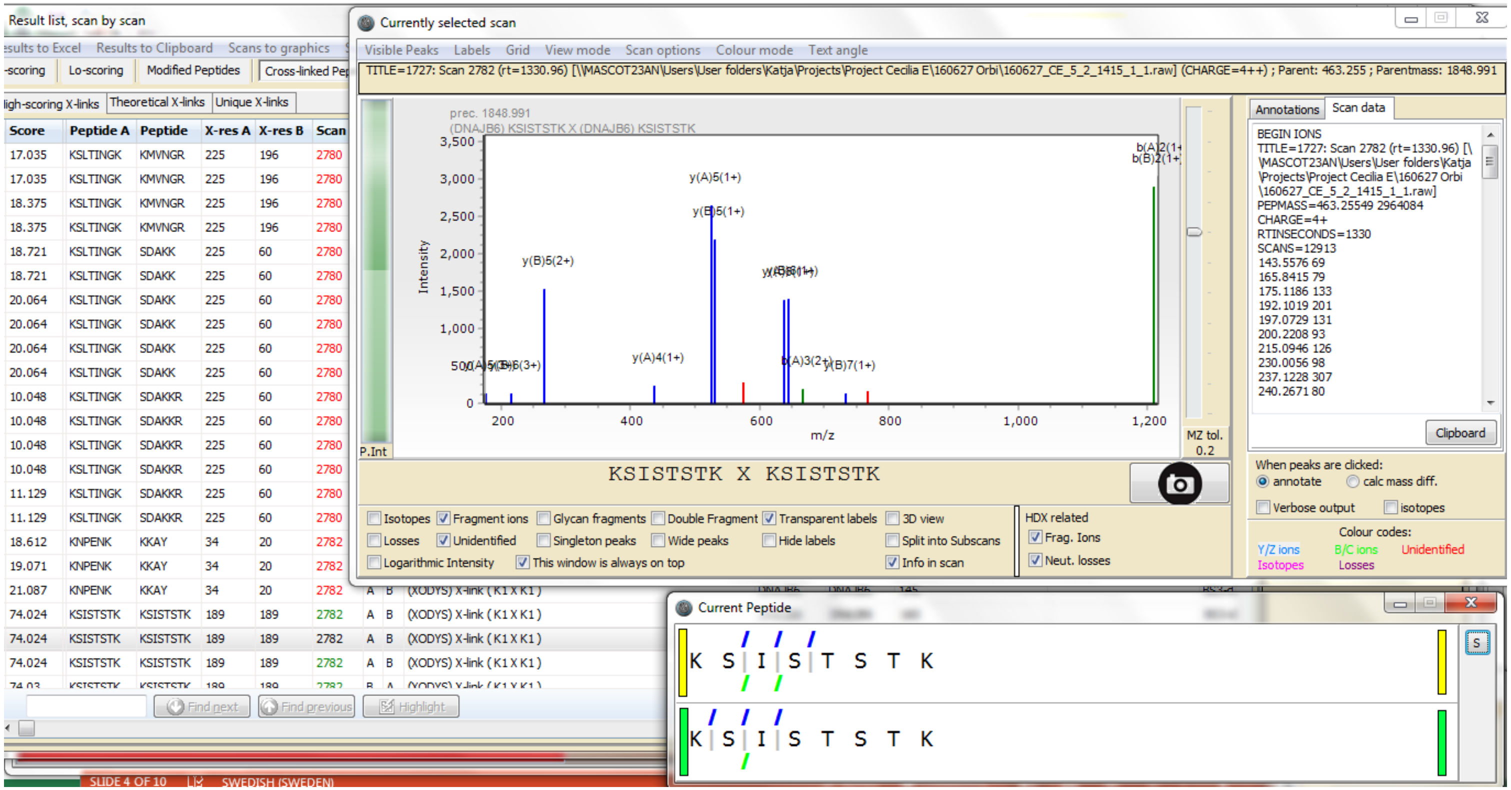

DNAJB6 K189-K189, KSISTSTK + KSISTSTK

Duplicate samples with crosslinked 1:1 mixture of unlabelled (^{14}N) and labeled (^{15}N) DNAJB6 oligomers

Fig. S7 MSMS-spectra for hybrid ^{14}N-^{15}N crosslinks as validation of K189-K189 and K232-K232 in Table 2

170625 Searches by CE on DNAJB6 for subunit exchange, mgf-files used:

Samples are insoldig (run in Lund 160627 by KB, processed by CE with Sept-version of crosslinked Cottrell .opt-file)

MassAI-search made with MassAI version Feb-17, and these settings:
mc 2, T and CT, tolerance 10 ppm MS, 0.1 Da for crosslinked peptides, tolerance 0.2 m/z in MSMS
No fixed modification: ~~IAA~~ Variable modification: BS4 as deadend and internal crosslink, 3 modifications, BS4 as crosslinks, Also Xlink modified peptides
XODYS
The mgf-files were passed through the 125-p filter

mgf-files: DNAJB6_5_1 and DNAJB6_5_2
in DNAJB6 sequence (A and B datasets = 14N and 15N)

K232-K232

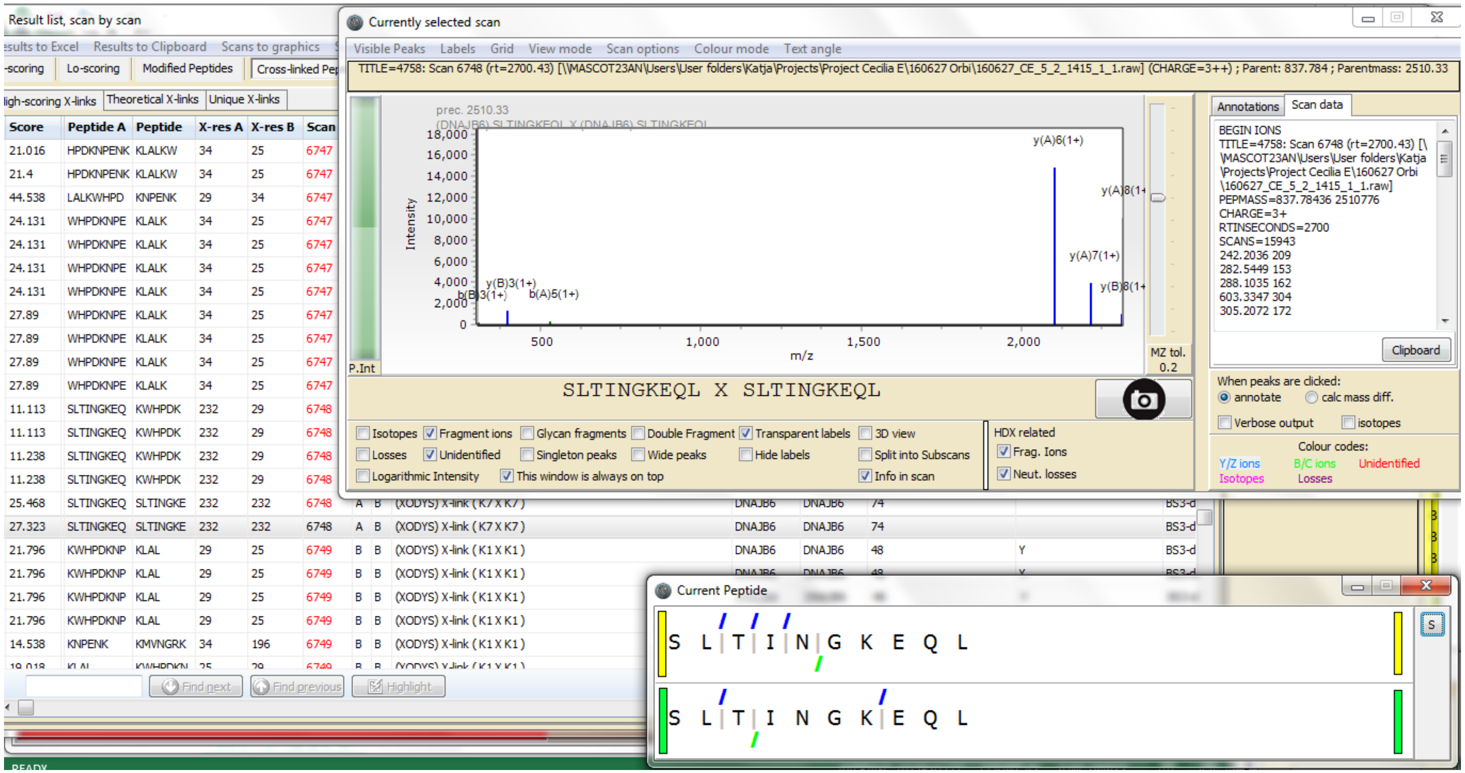

DNAJB6 K232-K232, SLTINGKEQLL + SLTINGKEQLLR

Duplicate samples with crosslinked 1:1 mixture of unlabelled (^{14}N) and labeled (^{15}N) DNAJB6 oligomers

Fig. S7 MSMS-spectra for hybrid ^{14}N-^{15}N crosslinks as validation of K189-K189 and K232-K232 in Table 2
